# 8-Oxoguanine DNA Glycosylase (OGG1) Deficiency Increases Susceptibility to Obesity and Metabolic Dysfunction

**DOI:** 10.1371/journal.pone.0051697

**Published:** 2012-12-17

**Authors:** Harini Sampath, Vladimir Vartanian, M. Rick Rollins, Kunihiko Sakumi, Yusaku Nakabeppu, R. Stephen Lloyd

**Affiliations:** 1 Department of Molecular and Medical Genetics, Center for Research on Occupational and Environmental Toxicology, Oregon Health and Science University, Portland, Oregon, United States of America; 2 Division of Neurofunctional Genomics, Department of Immunobiology and Neuroscience, Medical Institute of Bioregulation, and Research Center for Nucleotide Pool, Kyushu University, Fukuoka, Japan; University of Pecs Medical School, Hungary

## Abstract

Oxidative damage to DNA is mainly repaired via base excision repair, a pathway that is catalyzed by DNA glycosylases such as 8-oxoguanine DNA glycosylase (OGG1). While OGG1 has been implicated in maintaining genomic integrity and preventing tumorigenesis, we report a novel role for OGG1 in altering cellular and whole body energy homeostasis. OGG1-deficient (*Ogg1^−/−^*) mice have increased adiposity and hepatic steatosis following exposure to a high-fat diet (HFD), compared to wild-type (WT) animals. *Ogg1^−/−^* animals also have higher plasma insulin levels and impaired glucose tolerance upon HFD feeding, relative to WT counterparts. Analysis of energy expenditure revealed that HFD-fed *Ogg1^−/−^* mice have a higher resting VCO_2_ and consequently, an increased respiratory quotient during the resting phase, indicating a preference for carbohydrate metabolism over fat oxidation in these mice. Additionally, microarray and quantitative PCR analyses revealed that key genes of fatty acid oxidation, including carnitine palmitoyl transferase-1, and the integral transcriptional co-activator *Pgc-1α* were significantly downregulated in *Ogg1^−/−^* livers. Multiple genes involved in TCA cycle metabolism were also significantly reduced in livers of *Ogg1^−/−^* mice. Furthermore, hepatic glycogen stores were diminished, and fasting plasma ketones were significantly reduced in *Ogg1^−/−^* mice. Collectively, these data indicate that OGG1 deficiency alters cellular substrate metabolism, favoring a fat sparing phenotype, that results in increased susceptibility to obesity and related pathologies in *Ogg1^−/−^* mice.

## Introduction

If left unrepaired, oxidative damage to DNA from exogenous oxidants, as well as endogenous metabolic byproducts, can lead to cellular transformation and ultimately to the development of tumors. Non-bulky oxidative DNA lesions are mainly repaired via the base-excision repair (BER) pathway [1]–[3], which is initiated by DNA glycosylases, such as 8-oxoguanine DNA glycosylase (OGG1), Nei endonuclease VIII-like (NEIL)1, NEIL2, NEIL3, and endonuclease III-like 1 (NTH1). These enzymes recognize and excise specific subsets of lesions, and in some cases, further process the damaged site to a single-strand break via their intrinsic apurinic/apyrimidinic lyase activity [2]–[4].

7,8-dihydro-8-oxoguanine (8-oxoG) is one of the most commonly formed oxidative lesions in the cell. Since it mispairs with A during replication, 8-oxoG is also a highly mutagenic lesion, producing G:C to T:A transversions. OGG1, a DNA repair glycosylase that localizes to both the nucleus and mitochondria, is the main enzyme responsible for identification and excision of 8-oxoG lesions [5]–[9]. OGG1 is hypothesized to play a role in several disease pathways, including various cancers [4], [10]–[16], neurological diseases such as Parkinson’s [17]–[19] and Alzheimers’ [20]–[24] disease, and aging-related pathologies [25]–[28]. A common link between these pathologies is the presence of elevated levels of oxidative stress. Another disease state that is associated with increased levels of oxidative stress is that of metabolic syndrome, which encompasses several pathologies, including increased body weight and adiposity, fatty liver, elevated triglycerides, and impaired glucose tolerance.

While it is generally postulated that oxidative stress leads to progression of disease, the molecular mechanisms that mediate this process are as yet largely unknown. Since oxidative stress can lead to damage of cellular components, including DNA, we hypothesized that the absence of a critical DNA repair protein, such as in the case of OGG1-deficient mice, would lower the cellular tolerance for oxidative stress, such as that induced by consumption of a high-fat diet. We have therefore investigated the role of OGG1 in the development of metabolic disease and report here a novel role for OGG1 in the maintenance of cellular and whole body energy balance.

## Experimental Procedures

### Animal Studies

The generation of *Ogg1^−/−^* mice backcrossed 21 times to C57BL/6J background has been previously described [15]. At OHSU, the *Ogg1^−/−^* allele was maintained through additional backcrossing to C57BL/6J and subsequent matings between *Ogg1^+/−^* mice. Age-matched male mice were exclusively used throughout this investigation. Six mice of each genotype were assigned to either chow diet or high-fat diet (HFD); this number was deemed to be the minimum required for adequate statistical power for the desired analyses based on our previous studies and similar reports in the literature. The breeding and care of animals are in accordance with the protocols approved by the Animal Care and Use Committee of Oregon Health & Science University, Portland, Oregon (Protocol Number A967). Prior to euthanasia by cervical dislocation, mice were anesthetized by CO_2_ inhalation. For *in vivo* procedures, all efforts were made to minimize discomfort and suffering, in accordance with approved animal care protocols.?For the diet studies, 12-week old mice were individually housed and given *ad libitum* access to either rodent chow or a HFD (Research Diets D12492, New Brunswick, NJ; 60% fat, 20% protein, 20% carbohydrate by calories; 5.24 kcal/g metabolizable energy) for 10 weeks. Food intake and body weights were measured weekly during this period. Body composition was measured by low-resolution NMR (Echo Medical Systems, Houston, TX) prior to and 4 weeks after the start of the feeding study.

### Energy Expenditure

Energy expenditure was measured by indirect calorimetry (Oxymax, Columbus Instruments, Columbus, OH) as previously described [29], after 4 weeks of feeding. Briefly, oxygen consumption (VO_2_) and carbon dioxide production (VCO_2_) were simultaneously determined in individually housed animals with *ad libitum* access to chow or HFD and water. Following a 48-hour acclimation period, VO_2_ and VCO_2_ were recorded for 24 hours, including a 12-hour dark and 12-hour light phase. Samples were recorded every 2 minutes with a room air reference taken every 12 minutes. Respiratory exchange ratio (RER) was calculated as the molar ratio of VCO_2_: VO_2_.

### Glucose, Insulin, and Glycogen Measurements

Glucose tolerance was assessed 7 weeks after the start of the study and was carried out as previously described [29]. Briefly, mice were fasted for 4 hours followed by i.p. injection of 10% dextrose at a rate of 1g/kg body weight. Blood was collected at the indicated intervals for assessment of plasma glucose using the glucose color reagent (Cliniqa, San Marcos, CA). Fasting plasma insulin was measured by ELISA (Millipore, St. Charles, MO). Hepatic glycogen was measured by an enzyme coupled spectrophotometric assay, as previously described [29].

### Tissue Collection and Pathology

At the end of 10 weeks of feeding, mice were euthanized by CO_2_ overdose, and tissues were collected and snap frozen in liquid nitrogen for further analyses. For histological studies, liver and adipose tissues were fixed in buffered formalin and embedded in paraffin for sectioning and H&E staining. For transmission electron microscopy analyses, fresh liver sections were fixed and submitted to the Electron Microscopy Core at OHSU for sectioning and visualization. Buffer details are presented in Supplemental Methods.

### Tissue and Plasma Lipids

Total hepatic triglycerides (TG) were analyzed using the Wako total TG kit (Wako Chemicals, Richmond, VA). For fasting plasma ketone analyses, age-matched mice were fasted overnight, and blood was collected via saphenous vein puncture. Plasma ketones were measured using the plasma β-hydroxybutyrate kit (Cayman Chemical Co., Ann Arbor, MI).

### Physical Activity

Voluntary physical activity was assessed in chow-fed 12-week old male mice, as previously described [30]. Briefly, mice were acclimated to individual housing in shoebox cages equipped with running wheels for 7 days, and voluntary wheel running was measured for 2 consecutive weeks. Activity was monitored automatically using a Mini Mitter Magnetic Switch and the VitalView Data Acquisition System (Mini Mitter, Sun River, OR).

### Mitochondrial DNA Quantitation and Gene Expression Analysis

Mitochondrial DNA content was measured as previously described [29]. DNA microarray experiments were conducted at the Affymetrix Microarray Core of the OHSU Gene Microarray Shared Resource. Gene expression was measured using the Mouse Gene ST GeneChip Array (Affymetrix, Inc., Santa Clara, Calif, www.affymetrix.com). Data visualization and exploratory analysis were conducted using GeneSifter web-based software, release 59.0 (GeoSpiza Inc., Seattle, WA). Analysis, quality control, and statistical methods are presented in Supplemental Methods. All microarray data has been deposited in GEO and may be accessed via accession number GSE35497. RNA for quantitative real-time PCR (qPCR) was isolated using Tri-reagent RT (MRC, Inc., Cincinnati, OH). 1 µg of RNA was reverse-transcribed using the Superscript III first-strand synthesis system (Invitrogen, Carlsbad, CA). qPCR was performed on a Bio-Rad iCycler qPCR instrument (Bio-Rad, Hercules, CA) using gene-specific primers. Gene expression was normalized to expression of 18S rRNA. Primer sequences are available upon request.

### Statistical Analyses

**Figure 1:**
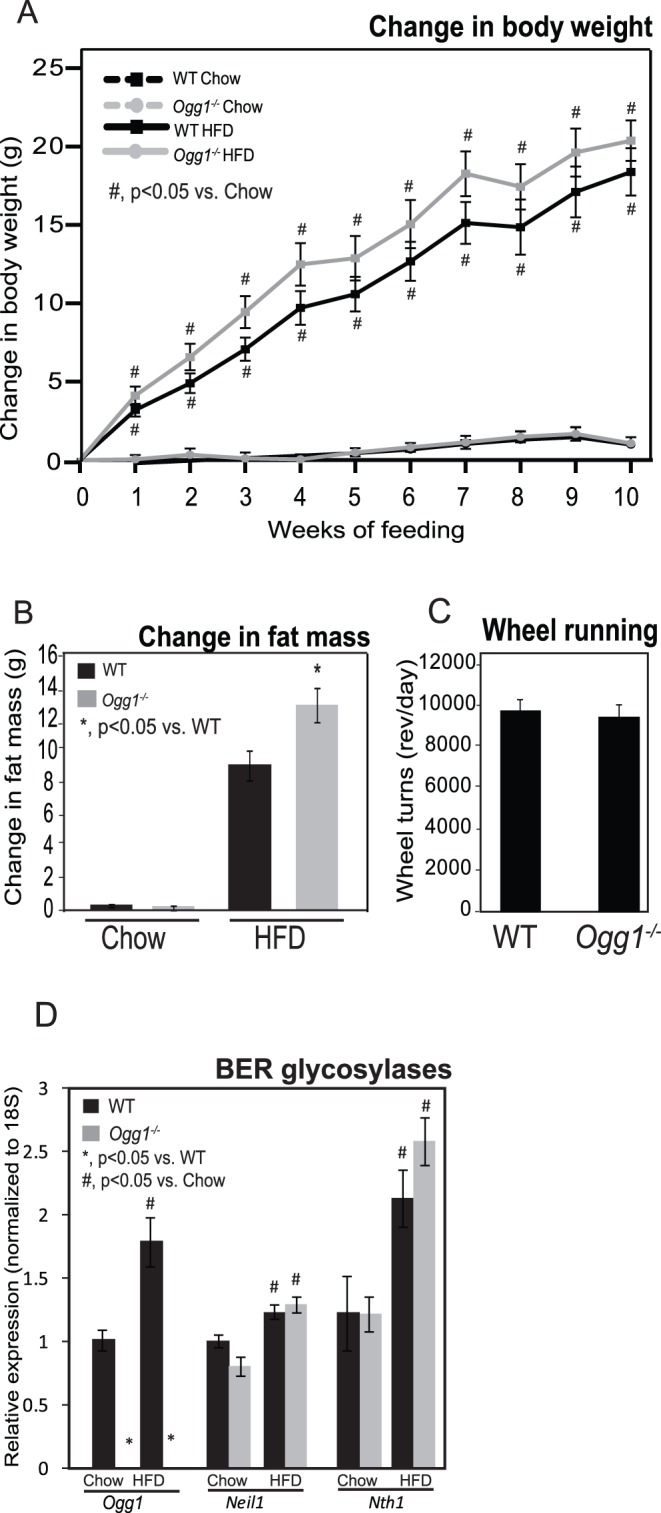
Body weight, body composition, voluntary activity, and gene expression of BER glycosylases. Body weights were measured weekly during the feeding period, and change in body weight from initial body weight was calculated (A). Body composition was measured by NMR at 0 and 4 weeks after the start of feeding, and change in fat mass was calculated (B). Voluntary physical activity was measured by recording wheel running in individually caged, chow-fed mice for 2 weeks (C). Expression of key glycosylases initiating BER of oxidized DNA was measured by qRT-PCR (D). n = 6 for each group; *, p<0.05 vs. WT, ^#^, p<0.05 vs. chow-fed mice.

Data are expressed as mean±SEM with comparisons carried out using student’s t-test for two-group comparisons (Figure 1C) or one-way ANOVA followed by post-hoc analysis using a multiple comparison procedure with Bonferroni/Dunn post-hoc comparison in Graphpad Prism. p-values <0.05 were considered significant.

## Results

### *Ogg1^−/−^* mice have an Increased Propensity to Adiposity, Especially Upon High-fat Diet Feeding

In a previous study examining potassium bromate-induced carcinogenesis in *Ogg1^−/−^* mice, data were also presented that suggested a trend towards increased body weights in *Ogg1^−/−^* mice, relative to wild-type (WT) counterparts that were maintained on a chow diet [31]. In order to determine if body weights and body composition are indeed significantly altered in *Ogg1^−/−^* animals, male mice were individually housed at 12 weeks of age and given *ad libitum* access to either chow or a hypercaloric high-fat diet (HFD). Over the duration of the 10-week study, chow-fed WT and *Ogg1^−/−^* mice displayed similar body weights (Figure 1A). HFD-feeding significantly increased weight gain in all mice, with WT mice gaining 17.5 g on average and *Ogg1^−/−^* counterparts gaining 20.4 g over 10 weeks of feeding (Figure 1A). However, total weight gain over the 10-week feeding period was not significantly different between WT and *Ogg1^−/−^* mice on either chow or HFD (Figure 1A). Although chow-fed *Ogg1^−/−^* mice were not significantly heavier at the end of the feeding study, in a separate cohort of animals allowed to age to 12–15 months on a chow diet, *Ogg1^−/−^* mice weighed 45.1±1.40 g (n = 5) on average, compared to WT counterparts that weighed 34.5±0.87 g (n = 6; p<0.05).

In addition to body weight, body composition was also measured by NMR before and 4 weeks after the start of feeding. Fat accumulation was significantly higher in HFD-fed *Ogg1^−/−^* mice, compared to WT counterparts (Figure 1B). While WT mice gained 9.14 g of fat (a 23% increase) upon HFD-feeding, *Ogg1^−/−^* mice gained 12.95 g of fat (a 28% increase) in the same period (Figure 1B). By 12–15 months of age, body fat in the aged cohort maintained on chow diet was also significantly different, with aged *Ogg1^−/−^* animals having 32.2% of their body weight as fat mass, compared to 19.5% body fat in WT counterparts (p<0.05). These data indicate that *Ogg1^−/−^* mice have an increased propensity to adiposity with age or in response to HFD-feeding, compared to WT controls.

At the end of the feeding period, visceral and subcutaneous adipose depots were collected and weighed. Both visceral (2.66±0.12 in WT vs. 2.91±0.19 g in *Ogg1^−/−^*; p>0.05) and subcutaneous (0.92±0.09 in WT vs. 1.28±0.06 g in *Ogg1^−/−^*; p<0.05 ) adipose depots tended to be larger in HFD-fed *Ogg1^−/−^* mice, relative to WT counterparts, indicating a generalized increase in fat accumulation in these mice. Voluntary activity (Figure 1C) and food intake were not significantly different between WT and *Ogg1^−/−^* mice either on chow (4.14±0.19 g in WT vs. 4.10±0.18 g in *Ogg1^−/−^*) or HFD (2.91±0.15 g in WT vs. 3.21±0.19 g in *Ogg1^−/−^*).

While a HFD is known to induce oxidative stress, the effect of extended high-fat feeding on expression of BER glycosylases has not been characterized. Therefore, we measured the expression of three key BER glycosylases, *Ogg1*, *Neil1*, and *Nth1*, in livers of chow- and HFD-fed mice (Figure 1D). HFD-feeding increased expression of all three glycosylases in WT mice, suggesting that the BER pathway is upregulated in response to HFD-feeding. *Neil1* and *Nth1* expression were increased by HFD-feeding in *Ogg1^−/−^* mice, as well. As expected, *Ogg1* expression was undetectable in *Ogg1^−/−^* animals.

### *Ogg1^−/−^* mice have Increased Hepatic Lipid Accumulation and Impaired Glucose Tolerance

**Figure 2:**
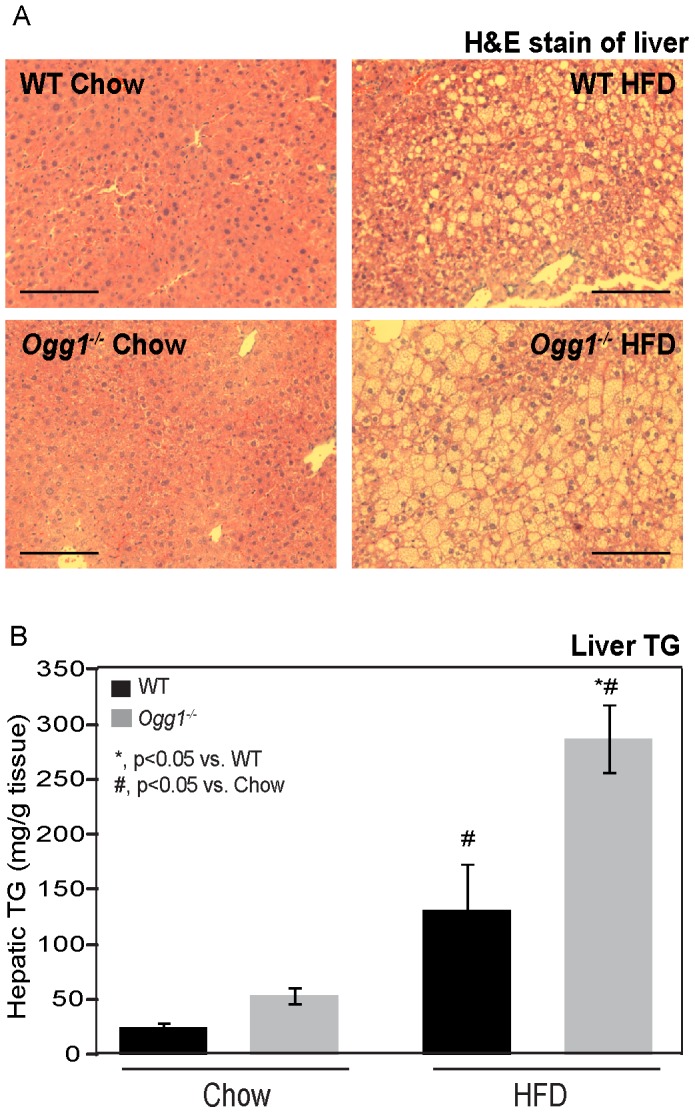
Hepatic lipid accumulation and triglyceride content. Hepatic lipids were visualized by H&E staining of formalin-fixed paraffin embedded liver tissues in chow- and HFD-fed animals (A). Images are representative of 3 animals per group. Bar represents 50 µM in all images. Hepatic triglyceride (TG) content (B) was quantified as described. n = 6 for each group; *, p<0.05 vs. WT, ^#^, p<0.05 vs. chow-fed mice.

Hepatic lipid accumulation was visualized by H&E staining, and total hepatic TG was quantified. Under chow-fed conditions, there were no significant differences in hepatic TG levels between WT and *Ogg1^−/−^* mice (Figures 2A, B). However, HFD-fed *Ogg1^−/−^* mice had a more than 2-fold higher accumulation of hepatic TG, compared to WT animals (Figures 2A, B).

**Figure 3:**
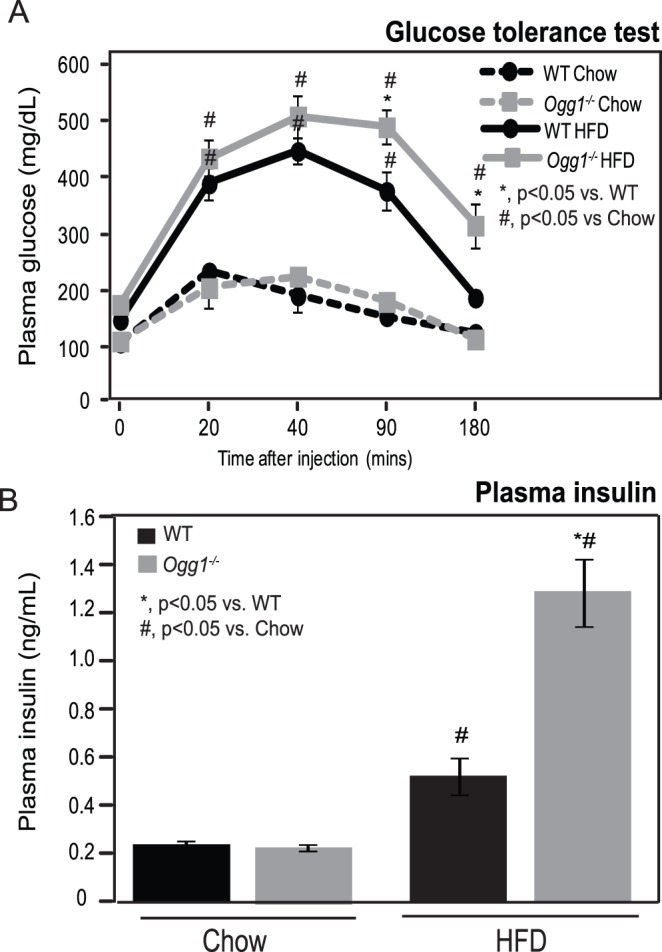
Glucose tolerance and plasma insulin. Glucose tolerance was measured by quantifying plasma glucose after an intraperitoneal injection of 1g glucose/kg BW in chow-fed and HFD-fed animals (A). Fasting plasma insulin was measured by ELISA prior to glucose injection (B). n = 6 for each group; *, p<0.05 vs. WT, ^#^, p<0.05 vs. chow-fed mice.

Since increased adiposity and hepatic lipid accumulation are risk factors for insulin resistance, glucose tolerance was assessed after 7 weeks of chow or HFD-feeding. After an intraperitoneal injection of glucose, chow-fed mice showed an increase in plasma glucose that returned to baseline levels by 180 minutes after injection (Figure 3A). As anticipated, HFD-feeding resulted in greater elevations in plasma glucose after glucose injection. While starting plasma glucose levels were comparable to WT values in HFD-fed *Ogg1^−/−^* mice, glucose clearance was significantly delayed in these animals. Plasma glucose levels at 90 and 180 minutes after glucose injection were significantly higher in HFD-fed *Ogg1^−/−^* mice, relative to WT counterparts (Figure 3A). Fasting plasma insulin levels were comparable in chow-fed WT and *Ogg1^−/−^* mice (Figure 3B). HFD-feeding increased fasting plasma insulin levels by 2.2-fold in WT mice and by 5.8-fold in *Ogg1^−/−^* animals (Figure 3B). In conjunction with the delayed glucose clearance (Figure 3A), the elevated plasma insulin levels in HFD-fed *Ogg1^−/−^* mice (Figure 3B) indicate a significant impairment in insulin sensitivity in these animals, relative to WT controls.

### Resting Respiratory Exchange Ratio, Fasting Plasma Ketones, and Hepatic Glycogen Content are Altered in *Ogg1^−/−^* mice

**Figure 4:**
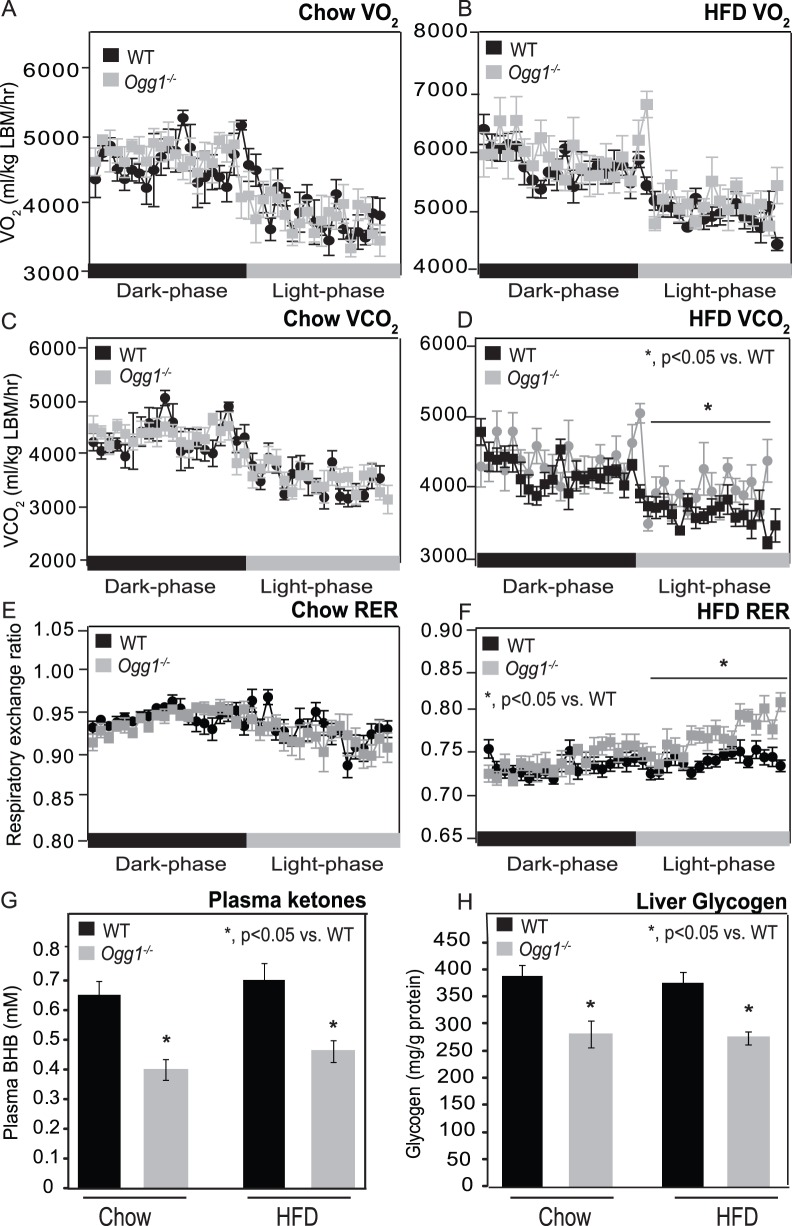
Indirect calorimetry, plasma ketones, and hepatic glycogen. O_2_ consumption (A, B), and CO_2_ production (C, D) were measured by indirect calorimetry in chow- or HFD-fed animals over consecutive dark and light cycles. Respiratory exchange ratio (RER) was calculated as VCO_2_/VO_2_ (E, F). Fasting plasma ketones were measured after an overnight fast (G). Hepatic glycogen (H) was measured in chow-fed and HFD-fed *Ogg1^−/−^* mice. n = 6 in each group; *, p<0.05 vs. WT.

In order to determine if basal metabolic rates were altered due to OGG1 deficiency, O_2_ consumption and CO_2_ production were measured in chow and HFD-fed mice. There were no significant differences in O_2_ consumption or CO_2_ production between chow-fed WT and *Ogg1^−/−^* mice (Figures 4A and 4B). After HFD-feeding, CO_2_ production during the resting phase was significantly higher in *Ogg1^−/−^* mice, relative to WT animals (Figure 4C and 4D). Consistently, the respiratory exchange ratio (RER), an indicator of substrate utilization, was also significantly increased during the resting phase in HFD-fed *Ogg1^−/−^* mice (Figure 4F), relative to WT animals, indicating a slight, but significant decrease in reliance on fatty acid oxidation for energy needs during the resting phase in these animals.

Fasting plasma ketones were measured after an overnight fast to obtain an additional measure of *in vivo* fatty acid oxidation. Interestingly, in chow-fed *Ogg1^−/−^* mice, fasting plasma ketones were reduced by 39% (p<0.05), relative to WT controls (Figure 4G). HFD-fed *Ogg1^−/−^* mice also had a similar 34% decrease (p<0.05) in fasting plasma ketones, indicating reduced rates of fatty acid oxidation in *Ogg1^−/−^* mice.

Based on the significant increase in RER (Figure 4F), we hypothesized that *Ogg1^−/−^* mice may preferentially utilize carbohydrate stores to meet energy needs. Therefore, hepatic glycogen content was measured and found to be diminished by 27% (p<0.05) in both chow-fed and HFD-fed *Ogg1^−/−^* mice, relative to WT counterparts (Figure 4H). Concomitantly, we also observed a slight, but significant increase in gene expression of two rate-limiting glycolytic enzymes, glucokinase (32%; p<0.05) and phosphofructokinase (23%; p<0.05), in livers of HFD-fed *Ogg1^−/−^* mice. Taken together with the reduced fasting plasma ketones and the significant increase in resting phase RER, these data are suggestive of a decreased reliance on fatty acids as a fuel source in *Ogg1^−/−^* mice.

Since OGG1 has both nuclear and mitochondrial localization, we sought to determine if mtDNA content or mitochondrial structure was altered in *Ogg1^−/−^* mice. mtDNA abundance was measured by PCR and was not consistently reduced in all *Ogg1^−/−^* mice (Supporting Figure S1A). TEM analysis of mitochondrial density and ultrastructure in liver did not reveal differences between WT and *Ogg1^−/−^* mice (Supporting Figure S1B).

### Hepatic Lipogenic Genes are not Upregulated in *Ogg1^−/−^* Livers

To gain further insight into the mechanistic changes underlying the metabolic phenotype of *Ogg1^−/−^* animals, hepatic gene expression was assessed through a combination of high-throughput DNA microarray and quantitative real-time PCR (qPCR). Analysis of differentially expressed probe sets (DEPs) by GeneSifter revealed 26 probesets (10 upregulated, 16 downregulated) to be altered by at least 1.5 fold in chow-fed *Ogg1^−/−^* livers, compared to WT livers (Supporting Table S1). After 10 weeks of HFD feeding, 1572 probesets were differentially expressed (132 upregulated and 1440 downregulated) in *Ogg1^−/−^* livers, compared to WT counterparts (Supporting Table S2). In addition to data analysis by GeneSifter, the Affymetrix data was simultaneously submitted to Ingenuity Systems for analysis via Ingenuity iReport for Gene Expression Analysis, the results of which were over 75% concordant with the GeneSifter analyses (Supporting Tables S3, S4). To investigate possible biological interactions and commonalities between the differentially regulated genes, DEPs identified by GeneSifter analyses were analyzed using Kyoto Encyclopedia of Genes and Genomes (KEGG) and gene ontology terms in GeneSifter, and pathways with a z-score greater than or equal to 2.0 and less than or equal to −2.0 were considered to be enriched or underrepresented, respectively (Supporting Tables S5, S6).

**Figure 5:**
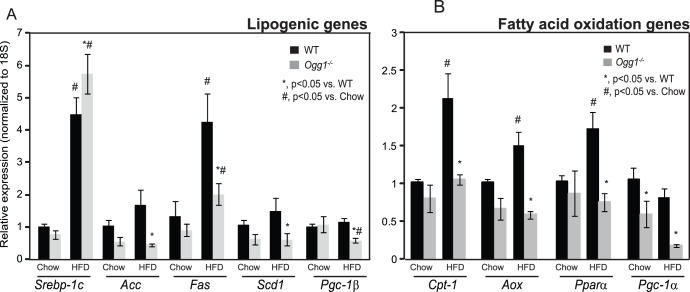
Hepatic lipogenic and fatty acid oxidation gene expression. Expression of key genes involved in hepatic lipogenesis (A) and fatty acid oxidation (B) were measured by qRT-PCR. n = 6 in each group; *, p<0.05 vs. WT, ^#^, p<0.05 vs. chow-fed mice.

Given the observations of metabolic dysfunction in *Ogg1^−/−^* mice, the microarray data were queried for potential mechanisms that may explain these phenotypes. Since increased hepatic TG can occur secondary to increased *de novo* lipogenesis, we sought to determine if hepatic lipogenic genes were differentially regulated in livers of *Ogg1^−/−^* mice. The master regulator of lipogenic genes, sterol regulatory element binding protein-1c (SREBP-1c, gene ID: *Srebf*), which also regulates its own gene expression, was increased by 1.9-fold in livers of HFD-fed *Ogg1^−/−^* mice (Supporting Tables S2, S4). Interestingly, none of the classical target genes of SREBP-1c, including acetyl-CoA carboxylase (*Acc*), fatty acid synthase (*Fas*) or stearoyl-CoA desaturase-1 (*Scd1*) were found to be upregulated by either microarray or by qPCR analyses (Figure 5A). In fact, all these genes were significantly lower in livers of HFD-fed *Ogg1^−/−^* mice, relative to WT counterparts. The expression of PPAR gamma co-activator -1β (*Pgc-1β*), a requisite co-activator of SREBP-1c, was also significantly lower in HFD-fed *Ogg1^−/−^* mice, relative to WT mice (Figure 5A). This downregulation of *Pgc1-β* may explain the lack of increase in hepatic lipogenic gene expression, despite a significant induction of *Srebp-1c*. Nevertheless, based on these results, the increase in hepatic lipids in *Ogg1^−/−^* mice (Figure 2A) does not appear to be a consequence of increased hepatic lipogenesis.

### Fatty Acid Oxidation and TCA Cycle Gene Expression is Reduced in *Ogg1^−/−^* Livers

Since resting phase RER was significantly higher and plasma ketones were reduced in HFD-fed *Ogg1^−/−^* mice, expression of genes involved in fat oxidation was examined. Consistent with a reduction in fatty acid oxidation, the microarray data revealed that several genes involved in fatty acid oxidation were significantly downregulated in HFD-fed *Ogg1^−/−^* livers, relative to WT counterparts (Table 1). Calcium-binding protein 39-like (*Cab39l*; also termed mouse protein 25 beta), electron-transferring-flavoprotein dehydrogenase (Etfdh), long-chain acyl-coenzymeA (CoA) synthetase isoforms 5 (*Acsl5*), AMP-activated protein kinase alpha subunit 2 (AMPK*α*2), PPAR-gamma coactivator -1 alpha (*Pgc-1α*), medium chain acyl-Coenzyme A dehydrogenase (*Acadm*), and peroxisomal enoyl-CoA delta isomerase (*Peci*), were all significantly downregulated by at least 1.5-fold in HFD-fed *Ogg1^−/−^* livers (Table 1). Furthermore, *Pgc-1α*, a key regulator of fatty acid oxidation gene expression in liver, was significantly lower in livers of chow-fed (Figure 5B), as well as HFD-fed (Table 1 and Figure 5B) *Ogg1^−/−^* mice. Concomitant with the reduction in *Pgc-1α*, additional key genes of fatty acid oxidation, including carnitine palmitoyl transferase-1 (*Cpt-1*), acyl CoA oxidase (*Aox*), and peroxisome proliferator-activated receptor alpha (*Pparα*)? were also decreased in HFD-fed *Ogg1^−/−^* mice, relative to WT counterparts (Figure 5B).

**Table 1:** Fatty acid oxidation genes.

Gene ID	Fold change	Direction	adjusted p-value	Fold change qRT-PCR	Direction	p-value
**Cab39l**	2.08	Down	0.00327	3.72	Down	7.73E-05
**Etfdh**	2.05	Down	0.002688	3.43	Down	0.000125
**Acsl5**	1.93	Down	0.011995	3.12	Down	0.00311
**Prkaa2**	1.88	Down	0.004445	2.98	Down	0.001322
**Ppargc1a**	1.73	Down	0.00455	4.78	Down	0.00406207
**Acadm**	1.51	Down	0.007172	2.26	Down	0.001677
**Peci**	1.51	Down	0.009513	1.91	Down	0.038155

Genes associated with fatty acid oxidation were identified from among the significantly differentially expressed probe sets between HFD-fed WT and *Ogg1^−/−^* mice, using gene ontology terms in Gene Sifter. Gene expression changes were confirmed by qRT-PCR using gene-specific primers. n = 6 in each group. *Cab39l*, calcium binding protein 39-like, *Etfdh*, electron transfer flavoprotein-ubiquinone oxidoreductase, *Acsl5*, acyl-CoA synthetase 5, *Prkaa2*, 5′-AMP-activated protein kinase catalytic subunit alpha-2, *Ppargc1a*, Peroxisome proliferator-activated receptor gamma coactivator 1-alpha, *Acadm*, medium-chain acyl-CoA dehydrogenase, *Peci*, peroxisomal 3,2-trans-enoyl-CoA isomerase.

In addition to fatty acid oxidation, PGC-1α also regulates genes involved in TCA cycle metabolism [32]. Consistent with the reduction in *Pgc-1α* levels (Table 1 and Figure 5B), several genes involved in the TCA cycle, including dihydrolipoamide dehydrogenase (*Dld*), malate dehydrogenase 1 (*Mdh1*), and fumarate hydratase (Fh) were all reduced by at least 1.5 fold in HFD-fed *Ogg1^−/−^* mice, as indicated by microarray analysis (Table 2). Additional genes involved in TCA cycle metabolism including isocitrate dehydrogenase 3 (*Idh3a*), pyruvate dehydrogenase E1 alpha 1 (*Pdha1*), succinate-Coenzyme A ligase (*Sucla2*), aconitase 1 (*Aco1*), succinate dehydrogenase complex, subunit A (*Sdha*) were all downregulated by at least 20% in HFD-fed *Ogg1^−/−^* livers (Table 2).

**Table 2:** TCA cycle metabolism genes.

Gene ID	Fold change	Direction	adjusted p-value	Fold change qRT-PCR	Direction	p-value
**Dld**	1.68	Down	0.013095	2.95	Down	0.000386
**Mdh1**	1.64	Down	0.004434	3.29	Down	0.000167
**Fh1**	1.63	Down	0.006485	3.06	Down	0.001282
**Idh3a**	1.48	Down	0.003829	2.02	Down	0.001583
**Pdha1**	**1.35**	Down	0.00929	1.86	Down	0.003487
**Sucla2**	**1.32**	Down	0.016633	2.10	Down	0.006097
**Aco1**	**1.28**	Down	0.003759	1.34	Down	0.013955
**Sdha**	**1.20**	Down	0.010029	1.56	Down	0.015946

Genes associated with TCA cycle metabolism were identified from among the significantly differentially expressed probe sets between HFD-fed WT and *Ogg1^−/−^* mice, using gene ontology terms in Gene Sifter. Gene expression changes were confirmed by qRT-PCR using gene-specific primers. n = 6 in each group. *Idh3a*, isocitrate dehydrogenase 3, *Pdha1*, pyruvate dehydrogenase E1 alpha 1, *Sucla2*, succinate-Coenzyme A ligase, *Aco1*, aconitase 1, *Sdha*, succinate dehydrogenase complex, subunit A.

## Discussion

We demonstrate here that the DNA repair glycosylase, OGG1, plays a novel role in regulating cellular energy metabolism. *Ogg1* expression is induced in response to a HFD (Figure 1D), suggesting that the repair of oxidative lesions such as 8-oxo-G by OGG1 are part of the cellular response to oxidative stress, such as that induced by a HFD. Conversely, in the absence of OGG1, a HFD led to accelerated development of several features of metabolic syndrome, including increased adiposity (Figure 1B), hepatic lipid accumulation (Figure 2), and impaired glucose tolerance upon high-fat feeding (Figure 3A). This was accompanied by increased resting RER (Figure 4F), reduced fasting plasma ketones (Figure 4G), and reduced expression of key genes of fat oxidation (Table 1 and Figure 5B), all indicating a shift in energy metabolism in *Ogg1^−/−^* mice away from fat oxidation. Taken together with the reduced hepatic glycogen content in *Ogg1^−/−^* livers (Figure 4H), these changes are indicative of a shift in cellular metabolism towards glucose utilization, over fat oxidation, in *Ogg1^−/−^* livers.

### Regulation of *Pgc-1* Transcriptional Co-activators and Fatty Acid Oxidation in Response to DNA Damage

A striking observation in *Ogg1^−/−^* mice was that of reduced levels of both *Pgc-1α* and *-1β* expression in liver. These two highly regulated transcriptional co-activators play critical roles in diverse cellular processes [33]–[35]. PGC-1β is a requisite co-activator for the lipogenic transcription factor, SREBP-1c [36]. Therefore, the reduction in *Pgc-1β* levels (Figure 5A) in *Ogg1^−/−^* livers may explain the lack of induction of *de novo* lipogenesis in *Ogg1^−/−^* mice, despite a significant increase in *Srebp-1c* message levels (Figure 5A).

PGC-1α plays tissue-specific roles in pathways ranging from mitochondrial biogenesis [35] to TCA cycle flux [32] and fatty acid oxidation [33]. Chow-fed and HFD-fed *Ogg1^−/−^* mice had significantly reduced expression of hepatic *Pgc-1α*, compared to WT counterparts. Consistently, while expression of *Cpt-1*, a *Pgc-1α* target gene, was significantly elevated in response to HFD-feeding in WT mice (Figure 5B), *Ogg1^−/−^* animals did not show a similar induction of *Cpt-1*. Furthermore, several additional genes of fatty acid oxidation were found to be reduced, especially after HFD-feeding, in *Ogg1^−/−^* mice (Table 1 and Figure 5B). In addition to these changes in gene expression, *Ogg1^−/−^* mice also had significantly reduced plasma ketone levels following an overnight fast (Figure 4G), suggesting decreased fatty acid oxidation, consistent with the observation of elevated resting phase RER (Figure 4F) in these mice.

The observation of reduced levels of *Pgc-1α* in *Ogg1^−/−^* mice after HFD is especially striking. PGC-1α has been demonstrated to be downregulated in response to DNA damage via both reduced transcription of the gene [37], as well as through increased protein degradation [3]. Furthermore, several groups have reported a decrease in *Pgc-1α* levels in breast and colon cancers [38]–[40]. Given the role of OGG1 in maintaining genomic integrity, it is therefore plausible that the reduction of *Pgc-1α* upon HFD-feeding is directly or indirectly related to the DNA repair functionality of OGG1, and that this downregulation of *Pgc-1α* mediates the metabolic phenotype observed in *Ogg1^−/−^* mice. Additional studies are underway in order to understand the precise nature of the downregulation of *Pgc-1α* in *Ogg1^−/−^* mice, including regulation of the PGC-1α protein. However, from the current study, it is clear that OGG1 deficiency alters the hepatic response to a HFD such that expression of *Pgc-1α,* as well as that of several of its key downstream target genes, is blunted in *Ogg1^−/−^* mice. To our knowledge, this is the first demonstration of a DNA repair protein being directly linked to the dietary regulation of *Pgc-1α* and as such, provides important potentially mechanistic information regarding the pathology of diet-induced metabolic disease.

### OGG1 Deficiency is Correlated with Human Metabolic Disease

In further support for a role for OGG1 in metabolic dysfunction, the Ser326Cys OGG1 polymorphism has been recently reported to be associated with Type II diabetes in both a Japanese and Mexican American population [41], [42]. While most studies investigating the consequences of human OGG1 polymorphisms have thus far been focused on the role of OGG1 in carcinogenesis, in light of the emerging evidence suggesting a role for OGG1 in maintaining energy balance, it will be interesting to determine if these common polymorphisms of human OGG1 are also associated with features of the metabolic syndrome. To our knowledge, studies investigating common gene polymorphisms and their correlations with increased body mass index have not thus far focused extensively on a role for DNA repair glycosylases such as *Ogg1* or *Neil1*. Given the emerging link between these glycosylases and metabolic syndrome, a retrospective screen of specimens collected in previous studies may help shed light on potential correlations between inactivating mutations in *Ogg1* and metabolic disease. In this regard, ongoing studies in our lab are aimed at characterizing the prevalence of common polymorphisms in the *Ogg1* gene, as well as the extent of tissue DNA damage in cohorts such as individuals undergoing bariatric surgery to determine the incidence of inactivating mutations in DNA repair genes in obese populations.

### OGG1 Acts through a Distinct Mechanism in Regulating Metabolic Parameters

We previously described a phenotype of spontaneous obesity and metabolic dysfunction in mice deficient in another BER glycosylase, NEIL1 [29], [43]. *Neil1^−/−^* mice spontaneously develop obesity on a chow diet, a phenotype that is greatly accelerated by HFD-feeding [29], [43]. This is accompanied by increased hepatic lipid accumulation and impairments in glucose tolerance in *Neil1^−/−^* mice [29], [43]. Since NEIL1 and OGG1 have very distinct substrate specificities in the repair of oxidized lesions, these similarities in phenotype with regard to the development of metabolic syndrome were completely unexpected. Indeed, a similar phenotype has not been observed in mice deficient in NEIL3 [44], despite considerable overlap in substrate specificities between NEIL1 and NEIL3. Interestingly, despite broad similarities in phenotype between *Neil1^−/−^* and *Ogg1^−/−^* mice, these two models exhibit important differences in the mechanisms underlying the observed pathologies. While overall energy expenditure was reduced in *Neil1^−/−^* mice [29], we did not observe any specific changes in RER [29] or fasting ketones in *Neil1^−/−^* animals (Sampath, Lloyd; unpublished), suggesting a generalized decrease in metabolic rate in *Neil1^−/−^* mice, rather than altered substrate preference, as observed in *Ogg1^−/−^* animals (Figures 4D,F). Furthermore, gene expression analysis of livers of *Neil1^−/−^* mice revealed a marked increase in inflammatory gene expression [29], while such a phenotype was not evident in the *Ogg1^−/−^* mice in the current study. This lack of an inflammatory response in *Ogg1^−/−^* livers, despite increased lipid accumulation, is consistent with other reports of an attenuated inflammatory response in the absence of OGG1 [45], [46], suggesting a possible role for excision of 8-oxoG and its subsequent release into the circulation in modulating the tissue inflammatory response.

**Figure 6:**
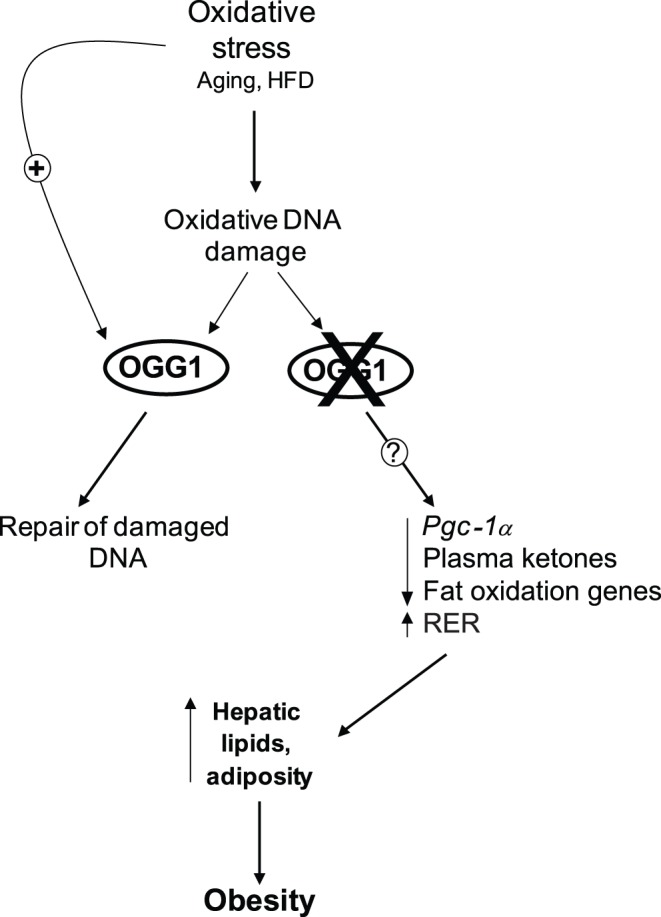
Proposed model for maintenance of metabolic health by OGG1. Oxidative stress, such as that induced by consumption of a HFD, damages cellular components, including DNA. Key BER glycosylases, such as *Ogg1*, are induced in response to this stress and help to restore genomic integrity. In contrast, in the absence of OGG1, through mechanisms that are as yet unclear, there is aberrant regulation of downstream factors such as *Pgc-1α* and fatty acid oxidation. These alterations lead to a potential fat-sparing phenotype in OGG1-deficient mice, resulting in accelerated development of hepatic steatosis and obesity upon HFD-feeding in *Ogg1^−/−^* mice.

Based on the results of the current study, we propose that oxidative stress, such as that resulting from HFD-feeding, results in DNA damage and a compensatory upregulation of the BER pathway to address this damage (Figures 1D, 6). In the absence of OGG1, a critical enzyme of the BER system, DNA damage goes unrepaired, leading to an aberrant response to a HFD in terms of regulation of *Pgc-1α* expression and fatty acid oxidation (Figures 4D, 4F, 4G, 5B, 6). Misregulation of these mediators leads to sparing of fat as a fuel and ultimately to lipid accumulation and obesity in *Ogg1^−/−^* mice (Figures 1B, 2, 6). These findings shed light on a novel pathway of metabolic regulation downstream of DNA damage and argue for an unexpected but critical role for OGG1 and the BER pathway in the maintenance of whole body energy balance.

## Supporting Information

Figure S1 **Mitochondrial DNA content and mitochondrial ultrastructure.** Mitochondrial DNA content (A, B) was measured by PCR using primers directed against two different regions of mitochondrial DNA. n = 5–6 in each group. CytoxII, cytochrome c oxidase, subunit 2; ND5, NADH dehydrogenase subunit 5. Mitochondrial density and ultrastructure in liver was also visualized by TEM (C). Images are representative of 3 animals per group. Scale bar represents 8.33 µM in all images.
(TIF)

Table S1 **Differentially expressed probesets (DEPs) identified by GeneSifter in chow-fed** ***Ogg1^−/−^*** **livers.** Pairwise analysis of chow-fed WT vs. *Ogg1^−/−^* was performed by t-test between the groups, followed by a Benjamini and Hochberg adjustment used to correct for false discovery rates using GeneSifter software. WT mice were designated as the control group, and *Ogg1^−/−^* mice were designated as the experimental group. Probesets that were differentially expressed by at least 1.5 fold and with an adjusted p<0.05 are presented. n = 6 in each group.
(DOC)

Table S2 **DEPs identified by GeneSifter in HFD-fed** ***Ogg1^−/−^*** **livers.** Pairwise analysis of HFD-fed WT vs. *Ogg1^−/−^* was performed by t-test between the groups, followed by a Benjamini and Hochberg adjustment used to correct for false discovery rates using GeneSifter software. WT mice were designated as the control group, and *Ogg1^−/−^* mice were designated as the experimental group. Probesets that were differentially expressed by at least 1.5 fold and with an adjusted p<0.05 are presented. n = 6 in each group.
(DOC)

Table S3 **DEPs identified by iReport in chow-fed** ***Ogg1^−/−^*** **livers.** Pairwise analysis of chow-fed WT vs. *Ogg1^−/−^* was conducted by iReport (Ingenuity Systems, Redwood City, CA), with WT mice were designated as the control group, and *Ogg1^−/−^* mice designated as the experimental group. Probesets that were differentially expressed by at least 1.5 fold and with an adjusted p<0.05 are presented. n = 6 in each group.
(DOC)

Table S4 **DEPs identified by iReport in HFD-fed** ***Ogg1^−/−^*** **livers.** Pairwise analysis of HFD-fed WT vs. *Ogg1^−/^*was conducted by iReport (Ingenuity Systems, Redwood City, CA), with WT mice were designated as the control group, and *Ogg1^−/−^* mice designated as the experimental group. Probesets that were differentially expressed by at least 1.5 fold and with an adjusted p<0.05 are presented. n = 6 in each group.
(DOC)

Table S5 **KEGG Pathway analysis of DEPs in chow-fed** ***Ogg1^−/−^*** **livers.** DEPs that were altered by at least 1.4 fold in chow-fed *Ogg1^−/−^* livers, relative to WT livers, were annotated using the Kyoto Encyclopedia of Genes and Genomes (KEGG) in GeneSifter. All pathways with an associated z-score >2.0 or <−2.0 are presented. n = 6 per group. n = 6 in each group.
(DOC)

Table S6 **KEGG Pathway analysis of DEPs in HFD-fed** ***Ogg1^−/−^*** **livers.** DEPs that were altered by at least 1.4 fold in HFD-fed *Ogg1^−/−^* livers, relative to WT livers, were annotated using the Kyoto Encyclopedia of Genes and Genomes (KEGG) in GeneSifter. All pathways with an associated z-score >2.0 or <−2.0 are presented. n = 6 per group.
(DOC)

Methods S1 **Supporting Methods.**
(DOC)
