# Supplementary figures and images for: 8-Oxoguanine DNA Glycosylase (OGG1) Deficiency Increases Susceptibility to Obesity and Metabolic Dysfunction
Source: PLoS One. 2012 Dec 17;7(12):e51697. doi: 10.1371/journal.pone.0051697 (PMC3524114; doi:10.1371/journal.pone.0051697)

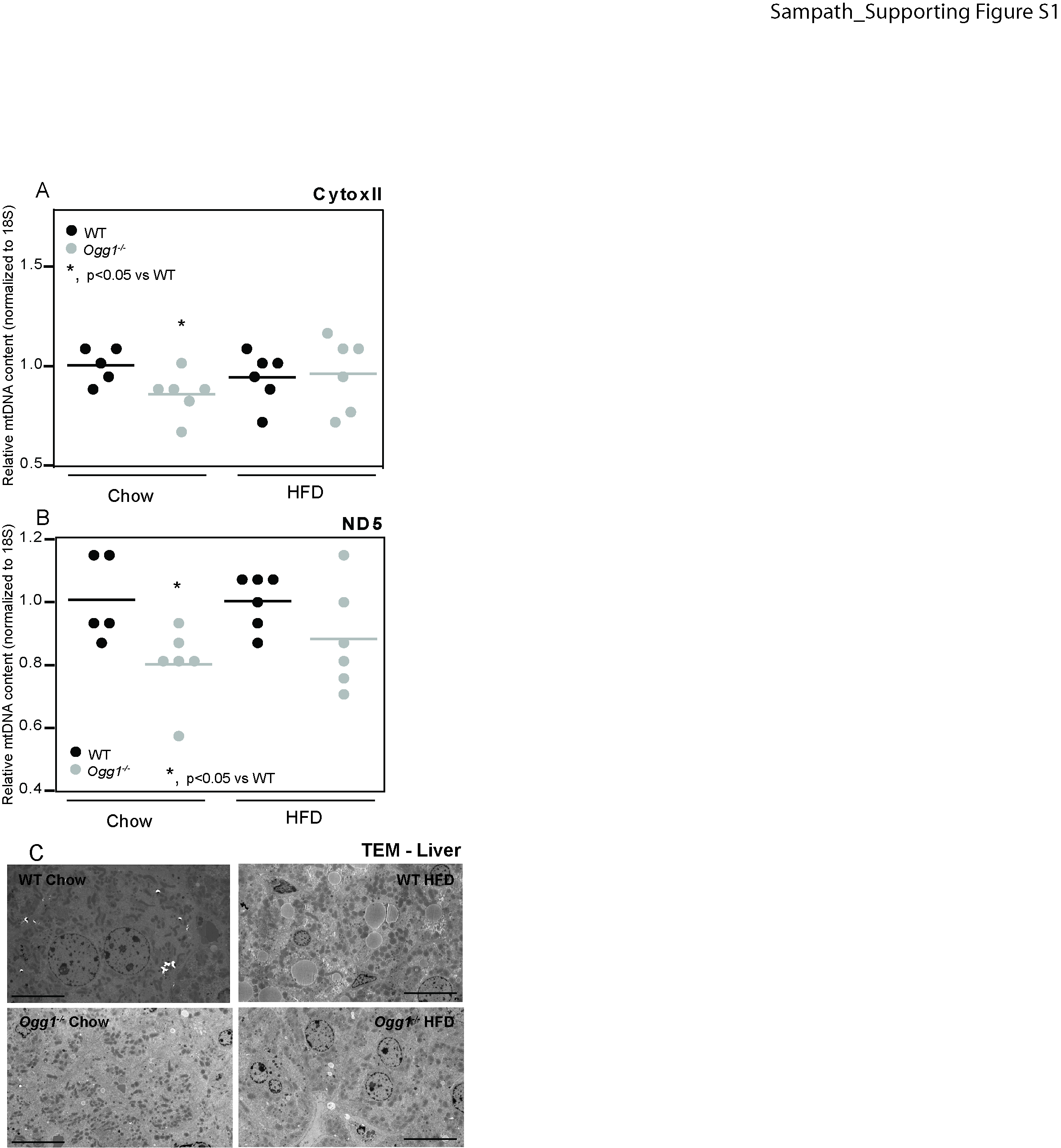

Supplement: Figure S1 — Mitochondrial DNA content and mitochondrial ultrastructure. Mitochondrial DNA content (A, B) was measured by PCR using primers directed against two different regions of mitochondrial DNA. n = 5–6 in each group. CytoxII, cytochrome c oxidase, subunit 2; ND5, NADH dehydrogenase subunit 5. Mitochondrial density and ultrastructure in liver was also visualized by TEM (C). Images are representative of 3 animals per group. Scale bar represents 8.33 µM in all images. (TIF) [file pone.0051697.s001.tif]
