# Supplementary material for: 8-Oxoguanine DNA Glycosylase (OGG1) Deficiency Increases Susceptibility to Obesity and Metabolic Dysfunction
Source: PLoS One. 2012 Dec 17;7(12):e51697. doi: 10.1371/journal.pone.0051697 (PMC3524114; doi:10.1371/journal.pone.0051697)
Supplement: Table S1 — Differentially expressed probesets (DEPs) identified by GeneSifter in chow-fed Ogg1−/− livers. Pairwise analysis of chow-fed WT vs. Ogg1−/− was performed by t-test between the groups, followed by a Benjamini and Hochberg adjustment used to correct for false discovery rates using GeneSifter software. WT mice were designated as the control group, and Ogg1−/− mice were designated as the experimental group. Probesets that were differentially expressed by at least 1.5 fold and with an adjusted p<0.05 are presented. n = 6 in each group. (DOC) [file pone.0051697.s002.doc]

**Supporting Table S1**: **Differentially expressed probe sets (DEPs) in chow-fed *Ogg1^-/-^* livers, compared to chow-fed WT livers, from Gene Sifter analysis**

| **Gene ID** | **Ratio** | **Direction** | **adj. p-value** |
| --- | --- | --- | --- |
| Murinoglobulin 2 (Mug2) | 8.53 | Down | 0.00776277 |
| Murinoglobulin 2 (Mug2) | 7.11 | Down | 0.00776277 |
| Gene model 129, (NCBI) | 6.42 | Up | 0.02071599 |
| Gene model 129, (NCBI) | 5.24 | Up | 0.0106036 |
| Ubiquitin specific peptidase 2 | 4.86 | Up | 0.00067568 |
| Basic helix-loop-helix family, member e41 (Bhlhe41) | 4.52 | Up | 0.00055686 |
| D site albumin promoter binding protein | 3.09 | Up | 0.01318762 |
| Aryl hydrocarbon receptor nuclear translocator-like | 2.94 | Down | 0.01258139 |
| WEE 1 homolog 1 (S. pombe) | 2.64 | Up | 0.00067568 |
| RIKEN cDNA 4933409K07 gene | 2.64 | Down | 0.0098155 |
| RIKEN cDNA 4933409K07 gene | 2.6 | Down | 0.01162746 |
| Transmembrane protein with EGF-like and two follistatin-like domains 2 | 2.46 | Up | 0.00055686 |
| RIKEN cDNA 4933409K07 gene | 2.4 | Down | 0.03366017 |
| RIKEN cDNA 4933409K07 gene | 2.4 | Down | 0.03366017 |
| PREDICTED: Mus musculus hypothetical protein LOC100039986 (LOC100039986) | 2.32 | Down | 0.00739919 |
| PREDICTED: Mus musculus hypothetical protein LOC100039986 (LOC100039986) | 2.16 | Down | 0.00739919 |
| RIKEN cDNA 4933409K07 gene | 2.07 | Down | 0.01595073 |
| RIKEN cDNA 4933409K07 gene | 2.02 | Down | 0.01595073 |
| Choline kinase alpha | 1.94 | Down | 0.01753803 |
| Thyrotroph embryonic factor (Tef), transcript variant 2 | 1.87 | Up | 0.01162746 |
| predicted gene 10628 | 1.71 | Down | 0.03424731 |
| BH3 interacting domain death agonist | 1.68 | Down | 0.0331303 |
| Kruppel-like factor 13 (Klf13) | 1.66 | Up | 0.00739919 |
| 3-phosphoadenosine 5-phosphosulfate synthase 1 (Papss1) | 1.62 | Down | 0.04707852 |
| Suppression of tumorigenicity 5 | 1.6 | Down | 0.04015151 |
| Sulfatase modifying factor 1 (Sumf1) | 1.6 | Up | 0.00347096 |
