# Supplementary material for: 8-Oxoguanine DNA Glycosylase (OGG1) Deficiency Increases Susceptibility to Obesity and Metabolic Dysfunction
Source: PLoS One. 2012 Dec 17;7(12):e51697. doi: 10.1371/journal.pone.0051697 (PMC3524114; doi:10.1371/journal.pone.0051697)
Supplement: Table S2 — DEPs identified by GeneSifter in HFD-fed Ogg1−/− livers. Pairwise analysis of HFD-fed WT vs. Ogg1−/− was performed by t-test between the groups, followed by a Benjamini and Hochberg adjustment used to correct for false discovery rates using GeneSifter software. WT mice were designated as the control group, and Ogg1−/− mice were designated as the experimental group. Probesets that were differentially expressed by at least 1.5 fold and with an adjusted p<0.05 are presented. n = 6 in each group. (DOC) [file pone.0051697.s003.doc]

**Supporting Table S2**: **DEPs in HFD-fed *Ogg1^-/-^*livers, compared to HFD-fed WT livers, from Gene Sifter analysis**

| **Gene Name*** | **Ratio** | **Direction** | **adj. p-value** |
| --- | --- | --- | --- |
| Murinoglobulin 2 (Mug2) | 8.65 | Down | 0.000489 |
| Cytochrome P450, family 2, subfamily b, polypeptide 9 (Cyp2b9) | 8.5 | Up | 0.008493 |
| Murinoglobulin 2 (Mug2) | 7.55 | Down | 0.000182 |
| Squalene epoxidase (Sqle) | 6.15 | Down | 0.025323 |
| Gene model 129, (NCBI) | 5.77 | Up | 0.001844 |
| Isopentenyl-diphosphate delta isomerase (Idi1), transcript variant 1 | 4.75 | Down | 0.009575 |
| Isopentenyl-diphosphate delta isomerase (Idi1), transcript variant 1 | 4.54 | Down | 0.010927 |
| Gene model 129, (NCBI) | 4.44 | Up | 0.001844 |
| Ubiquitin specific peptidase 2 | 3.77 | Up | 0.003863 |
| Cytochrome P450, family 51 | 3.73 | Down | 0.017983 |
| CDNA sequence BC023105 | 3.18 | Down | 0.011832 |
| HMG-CoA reductase mRNA, 3 end | 3.16 | Down | 0.045711 |
| 3-hydroxy-3-methylglutaryl-Coenzyme A synthase 1 | 3.14 | Down | 0.031178 |
| predicted gene, ENSMUSG00000071204 | 3.01 | Down | 0.0037 |
| Transmembrane protein 184C (Tmem184c) | 2.96 | Down | 0.001631 |
| NADH dehydrogenase subunit 6 | 2.79 | Up | 0.029073 |
| Fermitin family homolog 2 (Drosophila) | 2.74 | Down | 0.001744 |
| Polynucleotide phosphorylase-like protein (PNPASE gene) | 2.74 | Down | 0.004111 |
| 10582896 | 2.72 | Up | 0.020459 |
| Acyl-CoA thioesterase 3 (Acot3) | 2.71 | Up | 0.005501 |
| period homolog 1 (Drosophila) | 2.7 | Up | 0.003919 |
| NAD(P) dependent steroid dehydrogenase-like | 2.7 | Down | 0.034453 |
| Family with sequence similarity 55, member B (Fam55b) | 2.7 | Down | 0.000805 |
| MI0000256 Mus musculus miR-122 stem-loop | 2.68 | Down | 0.008438 |
| Circadian locomoter output cycles kaput (Clock) | 2.68 | Down | 0.002724 |
| Osteopontin (OPN) | 2.64 | Down | 0.010364 |
| 10598057 | 2.63 | Up | 0.015097 |
| Predicted gene, OTTMUSG00000000997 | 2.6 | Down | 0.005175 |
| Preimplantation protein 4 ) | 2.57 | Down | 0.01393 |
| Motile sperm domain containing 2 | 2.53 | Down | 0.004327 |
| Serine (or cysteine) peptidase inhibitor, clade A (alpha-1 antiproteinase, antitrypsin), member 12 (Serpina12) | 2.52 | Down | 0.002928 |
| PLZF gene | 2.49 | Up | 0.017985 |
| ClpX protein | 2.49 | Down | 0.001631 |
| Ganglioside-induced differentiation-associated-protein 2 (Gdap2) | 2.48 | Down | 0.002902 |
| Tsukushin | 2.45 | Up | 0.026974 |
| Eukaryotic translation initiation factor 2, subunit 2 (beta) (Eif2s2) | 2.45 | Down | 0.004805 |
| Proline-serine-threonine phosphatase-interacting protein 2 | 2.44 | Down | 0.015876 |
| Apolipoprotein B mRNA editing enzyme, catalytic polypeptide 1 (Apobec1), transcript variant 1 | 2.43 | Down | 0.002199 |
| Proteasome (prosome, macropain) 26S subunit, ATPase, 6 | 2.42 | Down | 0.007412 |
| Vacuolar protein sorting 41 (yeast) | 2.41 | Down | 0.003291 |
| predicted gene 10002 | 2.4 | Down | 0.001631 |
| Calcium/calmodulin-dependent protein kinase ID | 2.4 | Down | 0.002839 |
| DnaJ (Hsp40) homolog, subfamily C, member 3 | 2.37 | Down | 0.001744 |
| DnaJ (Hsp40) homolog, subfamily C, member 3 | 2.37 | Down | 0.001744 |
| SA protein (Sah) | 2.37 | Down | 0.005597 |
| Cadherin 1 (Cdh1) | 2.36 | Down | 0.010967 |
| COP9 (constitutive photomorphogenic) homolog, subunit 2 (Arabidopsis thaliana) | 2.35 | Down | 0.006758 |
| TRNA nucleotidyl transferase, CCA-adding, 1 | 2.35 | Down | 0.004877 |
| Protein phosphatase 2, regulatory subunit B, gamma (Ppp2r3c) | 2.34 | Down | 0.004348 |
| Karyopherin (importin) alpha 3 | 2.32 | Down | 0.003708 |
| Interferon-induced protein with tetratricopeptide repeats 3 | 2.32 | Down | 0.004045 |
| PREDICTED: Mus musculus similar to LOC360919 protein | 2.32 | Down | 0.007377 |
| Cullin 2 | 2.32 | Down | 0.002577 |
| Kelch-like 24 (Drosophila) (Klhl24) | 2.31 | Down | 0.004307 |
| LIM domain containing preferred translocation partner in lipoma (Lpp), transcript variant 2 | 2.3 | Down | 0.004932 |
| Carnitine O-octanoyltransferase (Crot) | 2.28 | Down | 0.002561 |
| Coagulation factor XI (F11) | 2.27 | Down | 0.00573 |
| RIKEN cDNA 1810055E12 gene | 2.27 | Down | 0.003537 |
| 10582916 | 2.27 | Up | 0.015631 |
| cdna:known chromosome:NCBIM37:9:3001070:3002330:1 gene:ENSMUSG00000074566 | 2.27 | Up | 0.046398 |
| Membrane associated DNA binding protein | 2.26 | Down | 0.003087 |
| Rnf125 mRNA for RNF125 protein | 2.25 | Down | 0.008985 |
| cdna:known chromosome:NCBIM37:7:26735272:26735442:1 gene:ENSMUSG00000053435 | 2.25 | Up | 0.010778 |
| MKIAA0433 protein | 2.25 | Down | 0.002409 |
| TRAF family member-associated Nf-kappa B activator | 2.24 | Down | 0.002561 |
| Zinc finger protein 146 (Zfp146) | 2.24 | Down | 0.001631 |
| Origin recognition complex, subunit 3-like (S. cerevisiae) (Orc3l) | 2.23 | Down | 0.004114 |
| Polybromo 1 | 2.23 | Down | 0.003361 |
| interferon inducible GTPase 1 | 2.23 | Down | 0.004556 |
| Mesoderm induction early response 1, family member 3 | 2.22 | Down | 0.002724 |
| Acyl-CoA synthetase long-chain family member 4 | 2.22 | Down | 0.010775 |
| Importin 7 | 2.22 | Down | 0.006464 |
| Chromosome segregation 1-like | 2.22 | Down | 0.006923 |
| Olfactory receptor 1034 | 2.21 | Down | 0.019959 |
| Vacuolar protein sorting 35 | 2.21 | Down | 0.005501 |
| Complement component 6 | 2.21 | Down | 0.004992 |
| ERGIC and golgi 2 (Ergic2), transcript variant 1 | 2.2 | Down | 0.003277 |
| Iron responsive element binding protein 2 (Ireb2) | 2.19 | Down | 0.002927 |
| Cullin 1 | 2.18 | Down | 0.002902 |
| CDNA fis, clone TRACH2008583,highly similar to CYTOCHROME P450 4A8 | 2.18 | Down | 0.046017 |
| Strain C57BL/6 StAR-related lipid transfer protein 4 (Stard4) | 2.18 | Down | 0.009742 |
| Chloride channel CLIC-like 1 (Clcc1) | 2.18 | Down | 0.001877 |
| Proteasome (prosome, macropain) 26S subunit, non-ATPase, 1 | 2.17 | Down | 0.002734 |
| Heat shock protein 90, beta (Grp94), member 1 | 2.16 | Down | 0.004265 |
| Carcinoembryonic antigen-related cell adhesion molecule 1 (Ceacam1), transcript variant 3 | 2.16 | Down | 0.001631 |
| Interferon-induced protein 44 | 2.16 | Down | 0.015395 |
| ncrna:snoRNA chromosome:NCBIM37:16:23111690:23111828:1 gene:ENSMUSG00000065208 | 2.15 | Up | 0.011132 |
| Carboxypeptidase B2 (plasma) (Cpb2) | 2.15 | Down | 0.006132 |
| NEDD8 activating enzyme E1 subunit 1 | 2.15 | Down | 0.005266 |
| Coatomer protein complex, subunit beta 1 | 2.14 | Down | 0.008008 |
| Major histocompatibility complex, class I-related | 2.14 | Down | 0.00527 |
| Phosphotriesterase related | 2.14 | Down | 0.002908 |
| Ankyrin repeat domain 49 | 2.13 | Down | 0.008832 |
| Ectonucleotide pyrophosphatase/phosphodiesterase 3 | 2.13 | Down | 0.002541 |
| Cell cycle associated protein 1 (Caprin1), transcript variant 1 | 2.13 | Down | 0.001223 |
| P450 (cytochrome) oxidoreductase | 2.13 | Up | 0.000451 |
| Integrator complex subunit 12 | 2.12 | Down | 0.002772 |
| Aftiphilin | 2.12 | Down | 0.001631 |
| Family with sequence similarity 175, member B | 2.11 | Down | 0.001744 |
| Insulin degrading enzyme (Ide) | 2.11 | Down | 0.010379 |
| RIKEN cDNA C730027P07 gene | 2.11 | Down | 0.002902 |
| COP9 (constitutive photomorphogenic) homolog, subunit 4 (Arabidopsis thaliana) (Cops4) | 2.1 | Down | 0.004027 |
| Cysteine conjugate-beta lyase 2 | 2.1 | Down | 0.002902 |
| TAB2 | 2.1 | Down | 0.001488 |
| Dyskeratosis congenita 1, dyskerin homolog (human) (Dkc1) | 2.1 | Down | 0.002249 |
| Ribosomal protein L7 (Rpl7) | 2.1 | Down | 0.006452 |
| UDP glucuronosyltransferase 2 family, polypeptide B34 (Ugt2b34) | 2.1 | Down | 0.002724 |
| Cytochrome P450, family 2, subfamily j, polypeptide 6 (Cyp2j6) | 2.09 | Down | 0.002409 |
| Sr528 protein | 2.09 | Down | 0.003092 |
| ZW10 interactor | 2.09 | Down | 0.004338 |
| SH3-binding domain glutamic acid-rich protein like | 2.09 | Down | 0.006166 |
| RIKEN cDNA C430048L16 gene | 2.09 | Down | 0.005728 |
| Mki67 (FHA domain) interacting nucleolar phosphoprotein (Mki67ip) | 2.09 | Down | 0.004245 |
| Mediator complex subunit 13 (Med13) | 2.09 | Down | 0.001632 |
| RNA and export factor binding protein 2 (Refbp2) | 2.08 | Up | 0.02042 |
| Calcium binding protein 39-like | 2.08 | Down | 0.00327 |
| IPLA2-2 | 2.07 | Down | 0.005254 |
| WW domain containing E3 ubiquitin protein ligase 1 | 2.07 | Down | 0.003967 |
| Phosphatidylinositol 3-kinase, catalytic, alpha polypeptide (Pik3ca) | 2.07 | Down | 0.002863 |
| Heat shock protein 90, alpha (cytosolic), class A member 1 (Hsp90aa1) | 2.06 | Down | 0.005035 |
| Sorting nexin 2 | 2.06 | Down | 0.002928 |
| Cytochrome P450, family 4, subfamily a, polypeptide 12a | 2.06 | Down | 0.041919 |
| murinoglobulin, pseudogene 1 | 2.06 | Down | 0.002561 |
| NCK-associated protein 1 | 2.06 | Down | 0.005254 |
| Mszf57 | 2.06 | Down | 0.002561 |
| PREDICTED: Mus musculus tankyrase, TRF1-interacting ankyrin-related ADP-ribose polymerase 2 (Tnks2) | 2.05 | Down | 0.001611 |
| D site albumin promoter binding protein | 2.05 | Up | 0.001921 |
| Coagulation factor IX mRNA, 3 end | 2.05 | Down | 0.008269 |
| Electron transferring flavoprotein, dehydrogenase (Etfdh) | 2.05 | Down | 0.002688 |
| Sr528 protein | 2.04 | Down | 0.004501 |
| Complement component 8, beta polypeptide | 2.04 | Down | 0.047078 |
| ncrna:snRNA chromosome:NCBIM37:6:149022525:149022628:-1 gene:ENSMUSG00000065198 | 2.04 | Down | 0.00582 |
| Ubiquitin specific peptidase 15 (Usp15) | 2.04 | Down | 0.003276 |
| Heat shock protein 90, alpha (cytosolic), class A member 1 (Hsp90aa1) | 2.03 | Down | 0.005259 |
| EFR3 homolog A (S. cerevisiae) (Efr3a) | 2.03 | Down | 0.002788 |
| Ectonucleotide pyrophosphatase/phosphodiesterase 2 | 2.03 | Down | 0.003328 |
| Dipeptidylpeptidase 4 | 2.03 | Down | 0.004854 |
| Predicted gene, EG245174 | 2.03 | Down | 0.011868 |
| heat shock protein 70 family, member 13 | 2.03 | Down | 0.004048 |
| Epidermal growth factor receptor pathway substrate 15 (Eps15) | 2.03 | Down | 0.003009 |
| RIKEN cDNA C530030P08 gene | 2.03 | Down | 0.003383 |
| DEAD (Asp-Glu-Ala-Asp) box polypeptide 1 (Ddx1) | 2.03 | Down | 0.002974 |
| RIKEN cDNA 2310001H12 gene | 2.03 | Down | 0.007491 |
| WD repeat domain 67 (Wdr67) | 2.03 | Down | 0.025009 |
| BH3 interacting domain death agonist | 2.03 | Down | 0.000592 |
| Coiled-coil domain containing 50 (Ccdc50), transcript variant 1 | 2.03 | Down | 0.002402 |
| Spermatid perinuclear RNA binding protein | 2.02 | Down | 0.004888 |
| Exocyst complex component 6 | 2.02 | Down | 0.008532 |
| CDNA clone IMAGE:9054298 | 2.02 | Down | 0.00273 |
| N-terminal EF-hand calcium binding protein 1 | 2.02 | Down | 0.002561 |
| taurine upregulated gene 1 | 2.02 | Down | 0.003977 |
| Gag protein | 2.02 | Up | 0.022697 |
| Granule cell antiserum positive 14 (Gcap14), transcript variant 2 | 2.01 | Down | 0.002598 |
| Pumilio 2 (Drosophila) | 2.01 | Down | 0.00691 |
| Cullin 4B | 2.01 | Down | 0.004908 |
| Tetraspanin 4 (Tspan4) | 2.01 | Up | 0.000561 |
| GC-rich promoter binding protein 1 | 2.01 | Down | 0.003252 |
| Amyloid beta precursor protein (cytoplasmic tail) binding protein 2 (Appbp2) | 2.01 | Down | 0.003229 |
| Signal peptidase complex subunit 3 homolog (S. cerevisiae) (Spcs3) | 2.01 | Down | 0.002577 |
| Glutamine and serine rich 1 | 2 | Down | 0.005354 |
| Peroxisomal biogenesis factor 3 | 2 | Down | 0.003405 |
| Predicted gene, EG240327 | 2 | Down | 0.003638 |
| Proteasome (prosome, macropain) activator subunit 4 | 2 | Down | 0.003085 |
| Complement component 4 binding protein | 2 | Down | 0.002254 |
| predicted gene, EG546714 | 2 | Down | 0.003863 |
| RIKEN cDNA C530030P08 gene | 1.99 | Down | 0.003462 |
| PHD finger protein 20-like 1 | 1.99 | Down | 0.003316 |
| Conserved helix-loop-helix ubiquitous kinase | 1.99 | Down | 0.003233 |
| Leucine zipper transcription factor-like 1 | 1.99 | Down | 0.039392 |
| Zinc finger protein 187 (Zfp187) | 1.98 | Down | 0.005102 |
| RIKEN cDNA E430025E21 gene | 1.98 | Down | 0.00446 |
| Leucyl/cystinyl aminopeptidase | 1.98 | Down | 0.008008 |
| Nuclear receptor coactivator 4 | 1.98 | Down | 0.003967 |
| Sarcolemma associated protein | 1.98 | Down | 0.003517 |
| Heat shock protein 90, alpha (cytosolic), class A member 1 (Hsp90aa1) | 1.98 | Down | 0.004734 |
| Signal recognition particle 72 (Srp72) | 1.98 | Down | 0.002254 |
| Acyl-CoA thioesterase 12 | 1.98 | Down | 0.002049 |
| DEAD (Asp-Glu-Ala-Asp) box polypeptide 58 | 1.98 | Down | 0.002908 |
| Zinc finger, MYM-type 2 | 1.98 | Down | 0.003361 |
| Protein kinase D3 | 1.98 | Down | 0.002249 |
| Strain ILS KIAA0103-like protein mRNA, partial sequence sequence | 1.97 | Down | 0.005569 |
| Cadherin 11 (Cdh11) | 1.97 | Down | 0.003674 |
| DEAH (Asp-Glu-Ala-His) box polypeptide 36 | 1.97 | Down | 0.008223 |
| MKIAA1230 protein | 1.97 | Down | 0.002561 |
| ATP-binding cassette, sub-family B (MDR/TAP), member 11 (Abcb11) | 1.97 | Down | 0.001611 |
| predicted gene, OTTMUSG00000007822 | 1.97 | Up | 0.046332 |
| Tetratricopeptide repeat domain 39B | 1.97 | Down | 0.004209 |
| Acyl-Coenzyme A binding domain containing 5 | 1.97 | Down | 0.004216 |
| Poly (A) polymerase alpha (Papola) | 1.97 | Down | 0.001614 |
| Protein kinase N2 (Pkn2) | 1.97 | Down | 0.004212 |
| Family with sequence similarity 126, member B | 1.97 | Down | 0.01235 |
| CDC14 cell division cycle 14 homolog B (S. cerevisiae) (Cdc14b), transcript variant 2 | 1.96 | Down | 0.005305 |
| High mobility group box transcription factor 1 (Hbp1), transcript variant 1 | 1.96 | Down | 0.004899 |
| SMEK homolog 2, suppressor of mek1 (Dictyostelium) (Smek2) | 1.96 | Down | 0.006125 |
| Ribosomal L1 domain containing 1 | 1.96 | Down | 0.004111 |
| Histone cluster 1, H2ab | 1.96 | Up | 0.00353 |
| Jumonji C domain-containing histone demethylase 1 homolog D (S. cerevisiae) | 1.95 | Down | 0.005462 |
| Transmembrane emp24 protein transport domain containing 5 (Tmed5) | 1.95 | Down | 0.005655 |
| Ribosomal protein L7 (Rpl7) | 1.95 | Down | 0.007901 |
| Interferon-induced protein with tetratricopeptide repeats 1 | 1.95 | Down | 0.0155 |
| Lecithin-retinol acyltransferase (phosphatidylcholine-retinol-O-acyltransferase) (Lrat) | 1.95 | Down | 0.013203 |
| Ring finger protein 160 | 1.94 | Down | 0.005367 |
| Superoxide-generating NADPH oxidase 4 (Nox4) | 1.94 | Down | 0.006729 |
| Expressed sequence AI451617 | 1.94 | Down | 0.007056 |
| Expressed sequence AI931714 | 1.94 | Down | 0.00195 |
| Dynamin 1-like (Dnm1l), transcript variant 1 | 1.94 | Down | 0.004898 |
| Signal transducing adaptor molecule (SH3 domain and ITAM motif) 2 | 1.94 | Down | 0.001488 |
| Praja 2, RING-H2 motif containing (Pja2), transcript variant 2 | 1.94 | Down | 0.010637 |
| Tripartite motif-containing 12 (Trim12) | 1.94 | Down | 0.005511 |
| APOBEC1 complementation factor (A1cf) | 1.94 | Down | 0.005662 |
| WEE 1 homolog 1 (S. pombe) | 1.93 | Up | 0.002902 |
| Polyhomeotic-like 3 (Drosophila) (Phc3) | 1.93 | Down | 0.015272 |
| RIKEN cDNA 1700112E06 gene | 1.93 | Down | 0.000782 |
| Acyl-CoA synthetase long-chain family member 5 | 1.93 | Down | 0.011995 |
| ATP-binding cassette, sub-family E (OABP), member 1 | 1.93 | Down | 0.002037 |
| Proline-rich nuclear receptor coactivator 2 | 1.93 | Down | 0.002927 |
| RIKEN cDNA 9830124H08 gene | 1.93 | Down | 0.005499 |
| CMAH mRNA for CMP-NeuAc hydroxylase, complete cds, major form of alternative splicing | 1.93 | Down | 0.005767 |
| Solute carrier family 10, member 2 (Slc10a2) | 1.92 | Down | 0.019628 |
| Myosin IB (Myo1b) | 1.92 | Down | 0.002639 |
| Magnesium transporter 1 (Magt1) | 1.92 | Down | 0.004268 |
| Ubiquitin-like domain containing CTD phosphatase 1 (Ublcp1) | 1.92 | Down | 0.008269 |
| FAST kinase domains 2 | 1.92 | Down | 0.003337 |
| Nardilysin, N-arginine dibasic convertase, NRD convertase 1 | 1.92 | Down | 0.003274 |
| Ribosomal protein L7 (Rpl7) | 1.92 | Down | 0.012885 |
| Janus kinase 1 | 1.92 | Down | 0.00331 |
| RMI1, RecQ mediated genome instability 1, homolog (S. cerevisiae) | 1.92 | Down | 0.003149 |
| Interleukin 1 alpha | 1.92 | Down | 0.004111 |
| RIKEN cDNA 4933409K07 gene | 1.92 | Down | 0.001838 |
| AF4/FMR2 family, member 4 | 1.92 | Down | 0.002927 |
| RIKEN cDNA 9030420J04 gene | 1.92 | Down | 0.002107 |
| Ribosomal protein L7 (Rpl7) | 1.92 | Down | 0.010507 |
| Nuclear receptor coactivator 4 | 1.92 | Down | 0.004081 |
| Transcribed locus, weakly similar to XP_001620419.1 hypothetical protein NEMVEDRAFT_v1g148203 [Nematostella vectensis] | 1.91 | Down | 0.037953 |
| predicted gene, EG621983 | 1.91 | Down | 0.00869 |
| Rho GTPase activating protein 29 | 1.91 | Down | 0.011118 |
| ncrna:snRNA chromosome:NCBIM37:1:72272814:72273004:1 gene:ENSMUSG00000075752 | 1.9 | Up | 0.019228 |
| metastasis associated lung adenocarcinoma transcript 1 (non-coding RNA) | 1.9 | Down | 0.022246 |
| Aldo-keto reductase family 1, member C14 | 1.9 | Down | 0.006771 |
| BetaKlotho protein putative polymorphic isoform | 1.9 | Down | 0.004204 |
| Clathrin, heavy polypeptide (Hc) | 1.9 | Down | 0.008051 |
| Transducin (beta)-like 1X-linked receptor 1 | 1.9 | Down | 0.005886 |
| Nudix (nucleoside diphosphate linked moiety X)-type motif 12 (Nudt12) | 1.9 | Down | 0.0037 |
| Ubiquitination factor E4A, UFD2 homolog (S. cerevisiae) (Ube4a) | 1.9 | Down | 0.003047 |
| Adaptor protein, phosphotyrosine interaction, PH domain and leucine zipper containing 1 (Appl1) | 1.9 | Down | 0.006734 |
| DEAD (Asp-Glu-Ala-Asp) box polypeptide 3, Y-linked | 1.9 | Down | 0.007491 |
| Sterol regulatory element binding protein 1 (Srebp1) | 1.9 | Up | 0.002928 |
| MARVEL (membrane-associating) domain containing 1 | 1.9 | Up | 0.001631 |
| RasGRP3 | 1.9 | Down | 0.002543 |
| Dihydrolipoamide branched chain transacylase E2 (Dbt), nuclear gene encoding mitochondrial protein | 1.89 | Down | 0.004932 |
| Hepcidin antimicrobial peptide 2 | 1.89 | Up | 0.004112 |
| Multiple C2 domains, transmembrane 2 (Mctp2) | 1.89 | Down | 0.003677 |
| DEAD (Asp-Glu-Ala-Asp) box polypeptide 52 | 1.89 | Down | 0.006222 |
| RIKEN cDNA 4930518I15 gene | 1.89 | Down | 0.003529 |
| Serine (or cysteine) peptidase inhibitor, clade B, member 9 | 1.89 | Down | 0.003229 |
| Secernin 3 | 1.89 | Down | 0.003928 |
| Smu-1 suppressor of mec-8 and unc-52 homolog (C. elegans) (Smu1) | 1.89 | Down | 0.00396 |
| RNA binding motif protein 39 | 1.88 | Down | 0.003689 |
| Leucine zipper transcription factor-like 1 | 1.88 | Down | 0.002863 |
| RIKEN cDNA 4932438A13 gene | 1.88 | Down | 0.010521 |
| Splicing factor 3b, subunit 1 (Sf3b1) | 1.88 | Down | 0.004327 |
| MI0005517 Mus musculus miR-568 stem-loop | 1.88 | Down | 0.011602 |
| DEK oncogene (DNA binding) | 1.88 | Down | 0.004521 |
| Protein kinase, AMP-activated, alpha 2 catalytic subunit | 1.88 | Down | 0.004445 |
| Nuclear receptor subfamily 3, group C, member 1 (Nr3c1) | 1.88 | Down | 0.002204 |
| RIKEN cDNA 5730601F06 gene (5730601F06Rik), transcript variant 1 | 1.88 | Down | 0.006431 |
| Trans-golgi network protein | 1.88 | Down | 0.003552 |
| CDNA clone MGC:198792 IMAGE:9054373 | 1.87 | Down | 0.009401 |
| Complement component 8, alpha polypeptide (C8a) | 1.87 | Down | 0.009606 |
| Mannosidase, endo-alpha | 1.87 | Down | 0.006135 |
| Ceruloplasmin (Cp), transcript variant 2 | 1.87 | Down | 0.004939 |
| CAMP-GEFII | 1.87 | Down | 0.004197 |
| Destrin (Dstn) | 1.87 | Down | 0.002928 |
| RAR-related orphan receptor alpha | 1.87 | Down | 0.005053 |
| Elongation of very long chain fatty acids (FEN1/Elo2, SUR4/Elo3, yeast)-like 2 (Elovl2) | 1.87 | Down | 0.002908 |
| Endomucin | 1.87 | Down | 0.009105 |
| Cullin 3 | 1.87 | Down | 0.002133 |
| Arginyl-tRNA synthetase (Rars) | 1.87 | Down | 0.004076 |
| Sorting nexin 13 (Snx13) | 1.87 | Down | 0.004045 |
| Guanine monphosphate synthetase | 1.86 | Down | 0.005254 |
| Ring finger protein 141 | 1.86 | Down | 0.003695 |
| WD repeat domain 43 | 1.86 | Down | 0.001921 |
| Suppressor of Ty 16 homolog (S. cerevisiae) | 1.86 | Down | 0.002254 |
| Guanylate-binding protein 10 (Gbp10) | 1.86 | Down | 0.015439 |
| MAK10 homolog, amino-acid N-acetyltransferase subunit, (S. cerevisiae) (Mak10) | 1.86 | Down | 0.003682 |
| Carboxypeptidase D (Cpd) | 1.86 | Down | 0.004505 |
| Ubiquitin protein ligase E3 component n-recognin 3 | 1.86 | Down | 0.005812 |
| RIKEN cDNA 4933409K07 gene | 1.86 | Down | 0.00195 |
| YME1-like 1 (S. cerevisiae) | 1.86 | Down | 0.007368 |
| Ankyrin repeat domain 28 (Ankrd28) | 1.86 | Down | 0.005013 |
| Muskelin 1, intracellular mediator containing kelch motifs | 1.86 | Down | 0.004695 |
| Immunity-related GTPase family M member 1 (Irgm1) | 1.86 | Down | 0.004185 |
| Growth arrest specific 2 | 1.85 | Down | 0.013901 |
| ATP-binding cassette, sub-family B (MDR/TAP), member 7 | 1.85 | Down | 0.008765 |
| Ring finger protein 141 | 1.85 | Down | 0.001921 |
| Sideroflexin 1 (Sfxn1) | 1.85 | Down | 0.003312 |
| PREDICTED: Mus musculus RIKEN cDNA 6820431F20 gene | 1.85 | Down | 0.004111 |
| RIKEN cDNA A430107O13 gene | 1.85 | Down | 0.003405 |
| Retinoic acid receptor, beta | 1.85 | Down | 0.018059 |
| Thyrotroph embryonic factor (Tef), transcript variant 2 | 1.85 | Up | 0.001744 |
| gi|34538597|ref|NC_005089.1|:c6938-6870, tRNA-Ser | 1.85 | Up | 0.018424 |
| Vanin 1 | 1.85 | Down | 0.007545 |
| Ubiquitin-specific processing protease (Usp25) | 1.85 | Down | 0.002147 |
| Ring finger protein 160 | 1.85 | Down | 0.004868 |
| Fas-interacting serine/threonine kinase 3 (Fist3) | 1.85 | Down | 0.002254 |
| Butyrylcholinesterase (Bche) | 1.85 | Down | 0.043274 |
| CDK5 regulatory subunit associated protein 1-like 1 (Cdkal1) | 1.85 | Down | 0.00577 |
| Sorting nexin 6 (Snx6) | 1.85 | Down | 0.005594 |
| DEAD/H (Asp-Glu-Ala-Asp/His) box polypeptide 3, X-linked (Ddx3x) | 1.85 | Down | 0.002409 |
| Elongation of very long chain fatty acids (FEN1/Elo2, SUR4/Elo3, yeast)-like 3 | 1.85 | Down | 0.00593 |
| ncrna:snoRNA chromosome:NCBIM37:3:123210855:123210984:-1 gene:ENSMUSG00000065113 | 1.85 | Up | 0.004633 |
| Neural precursor cell expressed, developmentally down-regulated 4 (Nedd4) | 1.84 | Down | 0.002808 |
| Inner membrane protein, mitochondrial (Immt), nuclear gene encoding mitochondrial protein | 1.84 | Down | 0.002908 |
| Signal recognition particle 54a | 1.84 | Down | 0.003667 |
| Protein-L-isoaspartate (D-aspartate) O-methyltransferase domain containing 1 | 1.84 | Down | 0.003382 |
| Microsomal triglyceride transfer protein | 1.84 | Down | 0.002653 |
|  | 1.84 | Down | 0.004655 |
| UDP-Gal:betaGlcNAc beta 1,4-galactosyltransferase, polypeptide 5 (B4galt5) | 1.84 | Up | 0.004433 |
| ncrna:rRNA chromosome:NCBIM37:11:74133294:74133412:1 gene:ENSMUSG00000070178 | 1.84 | Up | 0.000592 |
| K(lysine) acetyltransferase 2B (Kat2b) | 1.84 | Down | 0.003863 |
| GRAM domain containing 3 (Gramd3) | 1.84 | Down | 0.004172 |
| Bone morphogenetic protein receptor, type 1A (Bmpr1a) | 1.84 | Down | 0.004939 |
| RIKEN cDNA 2610301B20 gene (2610301B20Rik) | 1.84 | Down | 0.002349 |
| Adaptor protein complex AP-1, gamma 1 subunit (Ap1g1) | 1.84 | Down | 0.001844 |
| Zinc finger and BTB domain containing 33 (Zbtb33), transcript variant 1 | 1.84 | Down | 0.003229 |
| Neurobeachin like 1 | 1.84 | Down | 0.009575 |
| ncrna:rRNA chromosome:NCBIM37:9:102030218:102030335:1 gene:ENSMUSG00000065107 | 1.83 | Up | 0.006932 |
| Chaperonin containing Tcp1, subunit 4 (delta) (Cct4) | 1.83 | Down | 0.003119 |
| CCR4-NOT transcription complex, subunit 1 (Cnot1), transcript variant 1 | 1.83 | Down | 0.006208 |
| Fragile X mental retardation gene 1, autosomal homolog (Fxr1), transcript variant 3 | 1.83 | Down | 0.005603 |
| Predicted gene, EG624219 | 1.83 | Up | 0.01362 |
| RIKEN cDNA 2810482G21 gene | 1.83 | Down | 0.004779 |
|  | 1.83 | Up | 0.013901 |
| Mindbomb homolog 1 (Drosophila) (Mib1) | 1.83 | Down | 0.002541 |
| Nardilysin, N-arginine dibasic convertase, NRD convertase 1 | 1.83 | Down | 0.003095 |
| MKIAA1726 protein | 1.83 | Down | 0.002142 |
| cdna:Genscan chromosome:NCBIM37:10:75226549:75230103:1 | 1.83 | Up | 0.023294 |
| Basic helix-loop-helix family, member e41 (Bhlhe41) | 1.83 | Up | 0.045702 |
| Tubulin-specific chaperone E (Tbce) | 1.83 | Down | 0.00359 |
| Cold shock domain containing E1, RNA binding | 1.83 | Down | 0.003928 |
| RIKEN cDNA 4932438A13 gene | 1.83 | Down | 0.002601 |
| Ring finger protein 160 | 1.83 | Down | 0.004399 |
| splicing factor, arginine/serine-rich 18 | 1.83 | Down | 0.003387 |
| SEC63-like (S. cerevisiae) (Sec63) | 1.83 | Down | 0.002902 |
| Afamin (Afm) | 1.83 | Down | 0.004805 |
| TRNA splicing endonuclease 15 homolog (S. cerevisiae) | 1.83 | Up | 0.027954 |
| Attractin (Mgca) | 1.83 | Down | 0.001744 |
| Mus musculus, exportin 1, CRM1 homolog (yeast), clone IMAGE:5355327 | 1.82 | Down | 0.006125 |
| ATP-binding cassette, sub-family A (ABC1), member 6 (Abca6) | 1.82 | Down | 0.00828 |
| Zinc finger protein 317 | 1.82 | Down | 0.003875 |
| TAF2 RNA polymerase II, TATA box binding protein (TBP)-associated factor | 1.82 | Down | 0.003182 |
| DnaJ (Hsp40) homolog, subfamily C, member 10 | 1.82 | Down | 0.003595 |
| Serine dehydratase (Sds) | 1.82 | Up | 0.012594 |
| Tumor necrosis factor (ligand) superfamily, member 10 (Tnfsf10) | 1.82 | Down | 0.003928 |
| Tax1 (human T-cell leukemia virus type I) binding protein 1 | 1.82 | Down | 0.018787 |
| RIKEN cDNA 2310037I24 gene | 1.82 | Up | 0.005531 |
| Mitogen-activated protein kinase kinase kinase 7 | 1.82 | Down | 0.002012 |
| Coagulation factor XIII, beta subunit | 1.82 | Down | 0.004111 |
| Arylacetamide deacetylase-like 1 | 1.82 | Down | 0.004619 |
| Purine rich element binding protein B (Purb) | 1.82 | Down | 0.003552 |
| Transportin 3 | 1.82 | Down | 0.002046 |
| Defensin beta 1 | 1.82 | Down | 0.042687 |
| vomeronasal 2, receptor, pseudogene 14 | 1.82 | Down | 0.004045 |
| ATPase, class I, type 8B, member 1 | 1.82 | Down | 0.002598 |
| Hook homolog 3 (Drosophila) | 1.82 | Down | 0.015349 |
| Coatomer protein complex, subunit gamma 2 | 1.82 | Down | 0.002724 |
| Ubiquitin specific peptidase 16 | 1.82 | Down | 0.007515 |
| Pleiotropic regulator 1, PRL1 homolog (Arabidopsis) | 1.82 | Down | 0.003228 |
| Chemokine (C-X-C motif) ligand 9 | 1.81 | Down | 0.04562 |
| DNA segment, Chr 15, ERATO Doi 621, expressed (D15Ertd621e) | 1.81 | Down | 0.003447 |
| RING CCCH (C3H) domains 1 | 1.81 | Down | 0.002161 |
| Inhibitor of Bruton agammaglobulinemia tyrosine kinase | 1.81 | Down | 0.004323 |
| 5 nucleotidase, ecto (Nt5e) | 1.81 | Down | 0.026368 |
| Met proto-oncogene (Met) | 1.81 | Down | 0.004718 |
| Prolyl endopeptidase-like (Prepl) | 1.81 | Down | 0.002724 |
| ncrna:snoRNA chromosome:NCBIM37:18:75161109:75161173:1 gene:ENSMUSG00000064647 | 1.81 | Up | 0.025449 |
| Zinc finger CCCH type containing 14 | 1.81 | Down | 0.002249 |
| PREDICTED: Mus musculus A kinase (PRKA) anchor protein 11 (Akap11) | 1.81 | Down | 0.021145 |
| Family with sequence similarity 114, member A1 | 1.81 | Down | 0.002102 |
| Missing oocyte, meiosis regulator, homolog (Drosophila) (Mios) | 1.81 | Down | 0.003759 |
| NMD3 homolog (S. cerevisiae) (Nmd3) | 1.81 | Down | 0.004695 |
| Tripartite motif-containing 30 | 1.81 | Down | 0.003736 |
| CDNA fis, clone TRACH2017498 | 1.81 | Down | 0.00577 |
| Methylmalonyl-Coenzyme A mutase | 1.81 | Down | 0.008982 |
| Sirtuin 7 (silent mating type information regulation 2, homolog) 7 (S. cerevisiae) (Sirt7) | 1.81 | Up | 0.00609 |
| Cyclin D binding myb-like transcription factor 1 | 1.81 | Down | 0.005861 |
| cdna:known chromosome:NCBIM37:9:3037111:3038316:1 gene:ENSMUSG00000074558 | 1.81 | Up | 0.005713 |
| GTP binding protein 4 | 1.81 | Down | 0.006663 |
| Caspase 8 | 1.81 | Down | 0.00577 |
| cdna:pseudogene chromosome:NCBIM37:6:47753957:47754114:-1 gene:ENSMUSG00000045359 | 1.8 | Down | 0.001584 |
| Decorin (Dcn) | 1.8 | Down | 0.004695 |
| Hect domain and RLD 4 (Herc4) | 1.8 | Down | 0.003924 |
| RIKEN cDNA D030074E01 gene (D030074E01Rik) | 1.8 | Down | 0.009401 |
| NAD(P)H dehydrogenase, quinone 2 | 1.8 | Down | 0.007796 |
| RIKEN cDNA 4932438A13 gene | 1.8 | Down | 0.008424 |
| S-adenosylmethionine decarboxylase 1 | 1.8 | Down | 0.010734 |
| predicted gene, EG433224 | 1.8 | Down | 0.002598 |
| Carbonic anhydrase 1 | 1.8 | Down | 0.018354 |
| Hypoxia inducible factor 1, alpha subunit | 1.8 | Down | 0.007785 |
| Mus musculus similar to interferon-inducible GTPase (LOC435565) | 1.8 | Down | 0.002577 |
| Ring finger protein 160 | 1.8 | Down | 0.015431 |
| V-set and immunoglobulin domain containing 4 | 1.8 | Down | 0.004219 |
| Required for meiotic nuclear division 1 homolog (S. cerevisiae) | 1.8 | Down | 0.003672 |
| Coiled-coil domain containing 25 (Ccdc25) | 1.8 | Down | 0.005397 |
| Mus musculus RIKEN cDNA 2810487A22 gene | 1.8 | Down | 0.008663 |
| PDS5, regulator of cohesion maintenance, homolog A (S. cerevisiae) | 1.8 | Down | 0.003405 |
| Muscleblind-like 1 (Drosophila) | 1.8 | Down | 0.004323 |
| 10571599 | 1.8 | Down | 0.002086 |
| Basic leucine zipper and W2 domains 1 | 1.79 | Down | 0.005842 |
| MRNA of mkr3 gene encoding zinc finger protein | 1.79 | Down | 0.014897 |
| Mus musculus, clone IMAGE:5322051 | 1.79 | Down | 0.003429 |
| Calpain 7 (Capn7) | 1.79 | Down | 0.006431 |
| Sprouty 4 (Spry4) | 1.79 | Up | 0.030672 |
| ncrna:snoRNA chromosome:NCBIM37:9:15119633:15119765:1 gene:ENSMUSG00000064634 | 1.79 | Down | 0.01278 |
| Strain ILS cAMP response element binding protein 1 | 1.79 | Down | 0.004268 |
| Membrane protein, palmitoylated 5 (MAGUK p55 subfamily member 5) (Mpp5) | 1.79 | Down | 0.008414 |
| RIKEN cDNA 4833442J19 gene | 1.79 | Up | 0.0106 |
| Apoptosis inhibitor 5 | 1.79 | Down | 0.002561 |
| Inositol (myo)-1(or 4)-monophosphatase 1 (Impa1) | 1.79 | Down | 0.002402 |
| CDNA clone MGC:198792 IMAGE:9054373 | 1.79 | Down | 0.015169 |
| DEP domain containing 6 (Depdc6), transcript variant 1 | 1.79 | Down | 0.003401 |
| NudC domain containing 1 | 1.79 | Down | 0.002254 |
| Hypothetical protein (ORF1), clone Telethon(Italy_B41)_Strait00295_FL661-C7 | 1.78 | Down | 0.004774 |
| CCR4-NOT transcription complex, subunit 6 | 1.78 | Down | 0.004595 |
| Coenzyme Q10 homolog B (S. cerevisiae) (Coq10b), transcript variant 2 | 1.78 | Up | 0.009953 |
| Exocyst complex component 2 (Exoc2) | 1.78 | Down | 0.002457 |
| Zinc finger protein 719 | 1.78 | Down | 0.003405 |
| LysM, putative peptidoglycan-binding, domain containing 3 | 1.78 | Down | 0.002671 |
| RIKEN cDNA 9930021J03 gene | 1.78 | Down | 0.006268 |
| CDC-like kinase 1 (Clk1), transcript variant 2 | 1.78 | Down | 0.013628 |
| Zinc finger protein 654 | 1.78 | Down | 0.005102 |
| Acyl-coenzyme A amino acid N-acyltransferase 2 (Acnat2) | 1.78 | Up | 0.043904 |
| Transmembrane protein 69 | 1.78 | Up | 0.004283 |
| ADP-ribosylation factor guanine nucleotide-exchange factor 1(brefeldin A-inhibited) | 1.78 | Down | 0.015225 |
| CDNA clone IMAGE:3598196 | 1.78 | Down | 0.00327 |
| Pwcr1 mRNA, complete sequence | 1.78 | Up | 0.00814 |
| Cullin 5 | 1.78 | Down | 0.012309 |
| Mitogen-activated protein kinase kinase 4 (Map2k4) | 1.78 | Down | 0.00396 |
| RAS p21 protein activator 1 (Rasa1) | 1.78 | Down | 0.004384 |
| Folliculin interacting protein 1 | 1.77 | Down | 0.007238 |
| CDNA clone IMAGE:4485254 | 1.77 | Down | 0.004262 |
| Dynactin 4 | 1.77 | Down | 0.002832 |
| Kinesin family member 5B (Kif5b) | 1.77 | Down | 0.006708 |
| Thiopurine methyltransferase | 1.77 | Down | 0.004185 |
| SWI/SNF related, matrix associated, actin dependent regulator of chromatin, subfamily a, member 5 (Smarca5) | 1.77 | Down | 0.008493 |
| RIKEN cDNA 1700022C21 gene | 1.77 | Down | 0.008846 |
| gi|34538597|ref|NC_005089.1|:7700-7764, tRNA-Lys | 1.77 | Up | 0.045391 |
| Leucine-rich PPR-motif containing (Lrpprc) | 1.77 | Down | 0.005603 |
| MKIAA0678 protein | 1.77 | Down | 0.001752 |
| Dipeptidylpeptidase 8 | 1.77 | Down | 0.002609 |
| Nephronophthisis 3 (adolescent) (Nphp3), transcript variant 1 | 1.77 | Down | 0.002908 |
| ncrna:snRNA chromosome:NCBIM37:11:83085000:83085190:1 gene:ENSMUSG00000064856 | 1.77 | Up | 0.021942 |
| Superkiller viralicidic activity 2-like 2 (S. cerevisiae) (Skiv2l2) | 1.77 | Down | 0.006161 |
| RIKEN cDNA 4933411K20 gene (4933411K20Rik) | 1.77 | Down | 0.005261 |
| Exosome component 9 | 1.77 | Down | 0.004868 |
| Gametogenetin binding protein 2 (Ggnbp2) | 1.77 | Down | 0.009953 |
| RIKEN cDNA B430203M17 gene | 1.77 | Down | 0.017592 |
| Baculoviral IAP repeat-containing 3 | 1.77 | Down | 0.002737 |
| Eukaryotic translation initiation factor 5 | 1.77 | Down | 0.002331 |
| Mus musculus ubiquitin-like domain containing CTD phosphatase 1 (Ublcp1) | 1.77 | Down | 0.007309 |
| Pigeon homolog (Drosophila) | 1.77 | Down | 0.006421 |
| Ubiquitin specific petidase 45 (Usp45) | 1.77 | Down | 0.002923 |
| Von Willebrand factor A domain containing 5A | 1.77 | Down | 0.008584 |
| Sel-1 suppressor of lin-12-like (C. elegans) | 1.77 | Down | 0.001877 |
| S-adenosylmethionine decarboxylase 1 | 1.77 | Down | 0.017709 |
| Sec1 family domain containing 1 | 1.76 | Down | 0.00814 |
| SH3 domain binding glutamic acid-rich protein like 2 | 1.76 | Down | 0.006434 |
| DEP domain containing 7 (Depdc7) | 1.76 | Down | 0.007782 |
| Enhancer of polycomb homolog 2 | 1.76 | Down | 0.006693 |
| 10412258 | 1.76 | Down | 0.013664 |
| CDNA clone IMAGE:40048098 | 1.76 | Down | 0.005127 |
| Exportin 7 | 1.76 | Down | 0.003096 |
| Ubiquitin specific peptidase 33 | 1.76 | Down | 0.004429 |
| CDC like kinase 4 | 1.76 | Down | 0.006188 |
| RIKEN cDNA 1110018G07 gene | 1.76 | Down | 0.001838 |
| Mus musculus, clone IMAGE:5365035 | 1.76 | Down | 0.006434 |
| Melanoma inhibitory activity protein 2 | 1.76 | Down | 0.012614 |
| Myotubularin related protein 10 | 1.76 | Down | 0.003964 |
| Membrane-associated ring finger (C3HC4) 7 | 1.76 | Down | 0.014609 |
| RIKEN cDNA 3300001P08 gene | 1.76 | Down | 0.004388 |
| CDNA sequence BC048355 | 1.76 | Up | 0.003185 |
| Mitogen-activated protein kinase 6 | 1.76 | Down | 0.002161 |
| G protein-coupled receptor 110 (Gpr110) | 1.76 | Down | 0.041531 |
| Interferon regulatory factor 6 | 1.76 | Down | 0.01039 |
| Solute carrier family 30 (zinc transporter), member 4 (Slc30a4) | 1.76 | Down | 0.002902 |
| CDNA clone MGC:198792 IMAGE:9054373 | 1.76 | Down | 0.010624 |
| WNK lysine deficient protein kinase 1 | 1.76 | Down | 0.002189 |
| Signal recognition particle 54a | 1.76 | Down | 0.005222 |
| Optic atrophy 1 homolog (human) | 1.76 | Down | 0.013442 |
| Lectin, mannose-binding, 1 (Lman1) | 1.76 | Down | 0.001861 |
| SCY1-like 2 (S. cerevisiae) | 1.76 | Down | 0.002902 |
| O-sialoglycoprotein endopeptidase-like 1 | 1.76 | Down | 0.00844 |
| RIKEN cDNA 4932438A13 gene | 1.75 | Down | 0.010946 |
| BCL2-associated transcription factor 1 (Bclaf1), transcript variant 2 | 1.75 | Down | 0.003934 |
| Basic leucine zipper nuclear factor 1 | 1.75 | Down | 0.00317 |
| Cell division cycle 37 homolog (S. cerevisiae)-like 1 | 1.75 | Down | 0.011466 |
| Protein tyrosine phosphatase, non-receptor type 12 (Ptpn12) | 1.75 | Down | 0.002107 |
| MKIAA1347 protein | 1.75 | Down | 0.003379 |
| RIKEN cDNA 4921513D23 gene | 1.75 | Down | 0.002598 |
| Thymidine kinase 1 | 1.75 | Up | 0.005842 |
| FERM domain containing 4B | 1.75 | Down | 0.027733 |
| Oxysterol binding protein-like 8 | 1.75 | Down | 0.00359 |
| S-adenosylmethionine decarboxylase 1 | 1.75 | Down | 0.013726 |
| septin 2 | 1.75 | Down | 0.009763 |
| EGF, latrophilin seven transmembrane domain containing 1 (Eltd1) | 1.75 | Down | 0.003227 |
| PRP39 pre-mRNA processing factor 39 homolog (yeast) | 1.75 | Down | 0.007005 |
| SWI/SNF related, matrix associated, actin dependent regulator of chromatin, subfamily e, member 1 (Smarce1) | 1.75 | Down | 0.002103 |
| ncrna:snoRNA chromosome:NCBIM37:2:26768368:26768435:1 gene:ENSMUSG00000065146 | 1.75 | Up | 0.023499 |
| Mitochondrial translational initiation factor 2 (Mtif2), nuclear gene encoding mitochondrial protein | 1.75 | Down | 0.01099 |
| TBC1 domain family, member 8B | 1.75 | Down | 0.008029 |
| Serine (or cysteine) peptdiase inhibitor, clade B, member 8 | 1.75 | Down | 0.015416 |
| Isoleucine-tRNA synthetase | 1.75 | Down | 0.002653 |
| Aldehyde dehydrogenase 8 family, member A1 | 1.74 | Down | 0.001488 |
| Vacuolar protein sorting 39 (yeast) (Vps39), transcript variant 1 | 1.74 | Down | 0.000823 |
| Discs, large homolog 1 (Drosophila) (Dlg1) | 1.74 | Down | 0.002403 |
| Radixin | 1.74 | Down | 0.007431 |
| OTU domain containing 4 | 1.74 | Down | 0.001938 |
| T-complex protein 1 | 1.74 | Down | 0.006855 |
| Cleavage and polyadenylation factor subunit homolog (S. cerevisiae) ( | 1.74 | Down | 0.003695 |
| DCN1, defective in cullin neddylation 1, domain containing 4 (S. cerevisiae) | 1.74 | Down | 0.001031 |
| predicted gene, EG215974 | 1.74 | Up | 0.013315 |
| Mus musculus serine (or cysteine) peptidase inhibitor, clade A, member 3K (Serpina3k) | 1.74 | Down | 0.002961 |
| Mitogen-activated protein kinase kinase kinase kinase 3 | 1.74 | Down | 0.004399 |
| Cell division cycle 40 homolog (yeast) | 1.74 | Down | 0.003695 |
| Complement factor D (adipsin) (Cfd) | 1.74 | Down | 0.023153 |
| AN1, ubiquitin-like, homolog (Xenopus laevis) (Anubl1) | 1.74 | Down | 0.040857 |
| Kruppel-like factor 13 (Klf13) | 1.74 | Up | 0.00341 |
| RIKEN cDNA 1200011I18 gene | 1.74 | Down | 0.001778 |
| Mesoderm induction early response 1 homolog (Xenopus laevis | 1.73 | Down | 0.006383 |
| Cytidine and dCMP deaminase domain containing 1 | 1.73 | Down | 0.002378 |
| Transportin 1 | 1.73 | Down | 0.011124 |
| Ubiquitin specific peptidase 7 | 1.73 | Down | 0.002541 |
| Camello-like 4 | 1.73 | Up | 0.003879 |
| predicted gene, OTTMUSG00000008364 | 1.73 | Up | 0.006917 |
| Geranylgeranyl diphosphate synthase 1 | 1.73 | Down | 0.00302 |
| Imprinted and ancient | 1.73 | Down | 0.007214 |
| MKIAA4020 protein | 1.73 | Down | 0.016363 |
| RIKEN cDNA 3110001I20 gene | 1.73 | Down | 0.003545 |
| Tubulin, gamma complex associated protein 4 (Tubgcp4) | 1.73 | Down | 0.001744 |
| Zinc finger, CCHC domain containing 6 (Zcchc6) | 1.73 | Down | 0.003875 |
| Family with sequence similarity 69, member A (Fam69a) | 1.73 | Down | 0.001631 |
| Nitric oxide synthase trafficker | 1.73 | Down | 0.014111 |
| Ubiquitin specific peptidase like 1 (Uspl1), transcript variant 1 | 1.73 | Down | 0.003732 |
| Eukaryotic translation termination factor 1 (Etf1) | 1.73 | Down | 0.003695 |
| Zinc finger and BTB domain containing 20 | 1.73 | Down | 0.00607 |
| Trinucleotide repeat containing 6a (Tnrc6a) | 1.73 | Down | 0.002738 |
| BRCA1/BRCA2-containing complex, subunit 3 (Brcc3) | 1.73 | Down | 0.007128 |
| Peroxisome proliferative activated receptor, gamma, coactivator 1 alpha (Ppargc1a) | 1.73 | Down | 0.00455 |
| TBC1 domain family, member 15 (Tbc1d15) | 1.73 | Down | 0.008415 |
| RIKEN cDNA 1810074P20 gene | 1.73 | Down | 0.019715 |
| Nuclear antigen Sp100 (Sp100) | 1.73 | Down | 0.002927 |
| Sjogren syndrome antigen B | 1.73 | Down | 0.015194 |
| Complement component factor i (Cfi) | 1.73 | Down | 0.004912 |
| Eukaryotic translation initiation factor 2, subunit 2 (beta) (Eif2s2) | 1.73 | Down | 0.006352 |
| Optineurin (Optn) | 1.73 | Down | 0.002902 |
| Phosphodiesterase 9A (Pde9a) | 1.73 | Down | 0.000976 |
| ATPase, class VI, type 11B (Atp11b) | 1.72 | Down | 0.005624 |
| Solute carrier family 10 (sodium/bile acid cotransporter family), member 5 | 1.72 | Down | 0.006434 |
| UBX domain protein 4 (Ubxn4) | 1.72 | Down | 0.001853 |
|  | 1.72 | Down | 0.021107 |
| Aryl hydrocarbon receptor nuclear translocator-like | 1.72 | Down | 0.010895 |
| Myoneurin (Mynn) | 1.72 | Down | 0.005728 |
| Tripeptidyl peptidase II | 1.72 | Down | 0.00632 |
| Plastin 3 (T-isoform) (Pls3) | 1.72 | Down | 0.007403 |
| Peptidylprolyl isomerase domain and WD repeat containing 1 | 1.72 | Down | 0.025973 |
| SAC1 (suppressor of actin mutations 1, homolog)-like (S. cerevisiae) | 1.72 | Down | 0.004763 |
| Pyridoxal-dependent decarboxylase domain containing 1 (Pdxdc1), transcript variant 1 | 1.72 | Down | 0.002254 |
| Dmx-like 1 | 1.72 | Down | 0.006382 |
| Rho GTPase activating protein 18 | 1.72 | Down | 0.010507 |
| La ribonucleoprotein domain family, member 4 (Larp4), transcript variant 2 | 1.72 | Down | 0.014107 |
| Serine (or cysteine) peptidase inhibitor, clade A, member 3K | 1.72 | Down | 0.003025 |
| Solute carrier family 17 (sodium phosphate), member 4 | 1.72 | Down | 0.015904 |
| NOL1/NOP2/Sun domain family member 3 | 1.72 | Down | 0.004868 |
| Acyl-CoA thioesterase 4 (Acot4) | 1.72 | Up | 0.028528 |
| Serine/threonine kinase 4 (Stk4) | 1.72 | Down | 0.000805 |
| PREDICTED: Mus musculus hypothetical protein LOC100039986 (LOC100039986) | 1.72 | Down | 0.002192 |
| ncrna:snRNA chromosome:NCBIM37:11:83085000:83085190:1 gene:ENSMUSG00000064856 | 1.72 | Up | 0.022878 |
| ncrna:snRNA chromosome:NCBIM37:11:83085000:83085190:1 gene:ENSMUSG00000064856 | 1.72 | Up | 0.022878 |
| Schlafen 3 (Slfn3) | 1.72 | Up | 0.022878 |
| LIM and senescent cell antigen-like domains 1 (Lims1) | 1.72 | Down | 0.005447 |
| S-adenosylmethionine decarboxylase 1 | 1.72 | Down | 0.020108 |
| Protein disulfide isomerase associated 4 (Pdia4) | 1.72 | Down | 0.001838 |
| Caldesmon 1 (Cald1) | 1.72 | Down | 0.001631 |
| Cytoplasmic polyadenylation element binding protein 4 (Cpeb4) | 1.72 | Down | 0.005982 |
| Aldehyde oxidase 3 (Aox3) | 1.72 | Down | 0.008352 |
| Sphingosine-1-phosphate receptor 5 (S1pr5) | 1.72 | Up | 0.011294 |
| Family with sequence similarity 135, member A (Fam135a) | 1.71 | Down | 0.003462 |
| MU-2/AP1M2 domain containing, death-inducing (Mudeng) | 1.71 | Down | 0.012901 |
| DIS3 mitotic control homolog (S. cerevisiae) | 1.71 | Down | 0.005538 |
| Large subunit GTPase 1 homolog (S. cerevisiae) | 1.71 | Down | 0.002577 |
| Nudix (nucleoside diphosphate linked moiety X)-type motif 19 (Nudt19) | 1.71 | Up | 0.002928 |
| Serine racemase | 1.71 | Down | 0.002086 |
| cdna:pseudogene chromosome:NCBIM37:13:21472560:21472999:-1 gene:ENSMUSG00000081191 | 1.71 | Up | 0.034505 |
| PREDICTED: Mus musculus RIKEN cDNA 1700007B13 gene (1700007B13Rik) | 1.71 | Down | 0.017505 |
| Zinc finger protein 72 (Zfp72) | 1.71 | Down | 0.009743 |
| Hbs1-like (S. cerevisiae) | 1.71 | Down | 0.002037 |
| LMBR1 domain containing 2 | 1.71 | Down | 0.005051 |
| Mitochondrial ribosomal protein S36 | 1.71 | Up | 0.007355 |
| Proteasome (prosome, macropain) 26S subunit, non-ATPase, 14 | 1.71 | Down | 0.008788 |
| Importin 8 | 1.71 | Down | 0.002808 |
| RIKEN cDNA 2810007J24 gene | 1.71 | Down | 0.02456 |
| Cell division cycle 27 homolog (S. cerevisiae) | 1.71 | Down | 0.005501 |
| Coatomer protein complex, subunit beta 2 (beta prime) | 1.71 | Down | 0.003286 |
| N-myristoyltransferase 2 | 1.71 | Down | 0.007762 |
| Eukaryotic translation initiation factor 3, subunit J (Eif3j) | 1.71 | Down | 0.005501 |
| Heat shock 70kD protein 5 (glucose-regulated protein) | 1.71 | Down | 0.002109 |
| Transformed mouse 3T3 cell double minute 2 (Mdm2) | 1.71 | Down | 0.006431 |
| Syntrophin, basic 1 | 1.71 | Down | 0.006066 |
| RAB11 family interacting protein 2 (class I) | 1.71 | Down | 0.017609 |
| Purinergic receptor P2Y, G-protein coupled, 5 | 1.71 | Down | 0.023275 |
| RIKEN cDNA 4932441K18 gene | 1.71 | Down | 0.017225 |
| Wings apart-like homolog (Drosophila) | 1.71 | Down | 0.009293 |
| Grancalcin | 1.71 | Down | 0.0159 |
| X-ray repair complementing defective repair in Chinese hamster cells 5 | 1.71 | Down | 0.005692 |
| Dpy-19-like 4 (C. elegans) | 1.71 | Down | 0.00393 |
| RIKEN cDNA 1110008L16 gene | 1.71 | Down | 0.003291 |
| Zinc finger protein 809 | 1.71 | Down | 0.013567 |
| Leucine rich repeat containing 40 (Lrrc40) | 1.7 | Down | 0.005842 |
| Zinc finger, C3H1-type containing | 1.7 | Down | 0.002825 |
| Zinc finger, ZZ domain containing 3 (Zzz3), transcript variant 2 | 1.7 | Down | 0.005861 |
| Itchy, E3 ubiquitin protein ligase (Itch) | 1.7 | Down | 0.004116 |
| RIKEN cDNA 4921505C17 gene | 1.7 | Down | 0.011065 |
| Ubiquitin-like modifier activating enzyme 2 (Uba2) | 1.7 | Down | 0.002282 |
| CL-P1 mRNA for collectin placenta 1 | 1.7 | Down | 0.006068 |
| cdna:known chromosome:NCBIM37:17:66668413:66668852:-1 gene:ENSMUSG00000073376 | 1.7 | Up | 0.006343 |
| Ankyrin repeat and FYVE domain containing 1 | 1.7 | Down | 0.002549 |
| Expressed sequence AI314180 | 1.7 | Down | 0.005068 |
| Phenylalanyl-tRNA synthetase, beta subunit (Farsb) | 1.7 | Down | 0.007627 |
| Calcitonin receptor-like (Calcrl) | 1.7 | Down | 0.004469 |
| cdna:known chromosome:NCBIM37:2:150080254:150081327:-1 gene:ENSMUSG00000074735 | 1.7 | Down | 0.004739 |
| CTD (carboxy-terminal domain, RNA polymerase II, polypeptide A) small phosphatase like 2 (Ctdspl2) | 1.7 | Down | 0.002928 |
| Echinoderm microtubule associated protein like 4 | 1.7 | Down | 0.002008 |
| Stress-induced protein SIP18 (Sip) mRNA, complete cds, alternatively spliced | 1.7 | Down | 0.010697 |
| gi|34538597|ref|NC_005089.1|:c5257-5192, tRNA-Cys | 1.7 | Up | 0.044801 |
| Protection of telomeres 1A (Pot1a) | 1.7 | Down | 0.00632 |
| NEDD4 binding protein 2-like 1 (N4bp2l1) | 1.7 | Up | 0.00402 |
| Vimentin (Vim) | 1.7 | Down | 0.026777 |
| Capping protein (actin filament) muscle Z-line, alpha 1 (Capza1) | 1.7 | Down | 0.002902 |
| SUMO/Smt3-specific isopeptidase (SMT3IP3) | 1.7 | Down | 0.001714 |
| predicted gene 4989 | 1.7 | Up | 0.016833 |
| RIKEN cDNA 8430410K20 gene (8430410K20Rik) | 1.7 | Down | 0.002037 |
| Zinc finger protein 84 | 1.7 | Down | 0.001611 |
| Ring finger protein 160 | 1.7 | Down | 0.035909 |
| Deltex 3-like (Drosophila) | 1.7 | Down | 0.002012 |
| Syntaxin 17 (Stx17) | 1.7 | Down | 0.006692 |
| RIKEN cDNA 4933409K07 gene | 1.7 | Down | 0.002254 |
| Sorting nexin 4 (Snx4) | 1.69 | Down | 0.002457 |
| Ubiquitin specific peptidase 37 | 1.69 | Down | 0.003352 |
| exocyst complex component 6B | 1.69 | Down | 0.004268 |
| Ubiquitin specific peptidase 24 | 1.69 | Down | 0.003595 |
| MKIAA0678 protein | 1.69 | Down | 0.015423 |
|  | 1.69 | Down | 0.002928 |
| Lectin, galactose binding, soluble 8 (Lgals8) | 1.69 | Down | 0.001844 |
| Nicotinamide N-methyltransferase | 1.69 | Up | 0.010962 |
| Mid1 interacting protein 1 (gastrulation specific G12-like (zebrafish)) | 1.69 | Up | 0.006932 |
| MI0004687 Mus musculus miR-703 stem-loop | 1.69 | Down | 0.012489 |
| Expressed sequence AI987944 | 1.69 | Down | 0.022971 |
| IWS1 homolog (S. cerevisiae) | 1.69 | Down | 0.012239 |
| Phosphorylase kinase beta (Phkb) | 1.69 | Down | 0.00669 |
| Eukaryotic translation initiation factor 2a (Eif2a) | 1.69 | Down | 0.01123 |
| Tripartite motif protein TRIM33 (Trim33) | 1.69 | Down | 0.002908 |
| WD repeat domain 48 | 1.69 | Down | 0.002331 |
| Ngfi-A binding protein 1 | 1.69 | Down | 0.003541 |
| GA repeat binding protein, alpha | 1.69 | Down | 0.00577 |
| MEF2A mRNA, partial 3 UTR sequence | 1.69 | Down | 0.006672 |
| FCH domain only 2 | 1.69 | Down | 0.011836 |
| CDNA clone IMAGE:9053676 | 1.69 | Down | 0.006706 |
| COP9 (constitutive photomorphogenic) homolog, subunit 3 (Arabidopsis thaliana) (Cops3) | 1.69 | Down | 0.007674 |
| Phosphoglucomutase 3 (Pgm3) | 1.69 | Down | 0.025078 |
| Interleukin 1 receptor accessory protein (Il1rap), transcript variant 2 | 1.69 | Down | 0.005254 |
| Suppression of tumorigenicity 7-like (St7l) | 1.69 | Down | 0.004433 |
| Mitogen-activated protein kinase kinase kinase 7 interacting protein 3 (Map3k7ip3) | 1.69 | Down | 0.004339 |
| CDC28 protein kinase 1b (Cks1b) | 1.69 | Up | 0.00399 |
| Myotubularin related protein 6 (Mtmr6) | 1.69 | Down | 0.006855 |
| SMT3 suppressor of mif two 3 homolog 1 (yeast) (Sumo1) | 1.69 | Up | 0.036623 |
| Sno, strawberry notch homolog 1 (Drosophila) | 1.69 | Down | 0.003119 |
| cdna:pseudogene chromosome:NCBIM37:3:100217483:100218483:1 gene:ENSMUSG00000081440 | 1.69 | Up | 0.004027 |
| Male-specific lethal 3 homolog (Drosophila) | 1.69 | Down | 0.003526 |
| MKIAA1726 protein | 1.69 | Down | 0.003759 |
| Membrane magnesium transporter 1 (Mmgt1) | 1.69 | Down | 0.004399 |
| Copine III | 1.68 | Down | 0.011903 |
| cdna:pseudogene chromosome:NCBIM37:2:40411272:40412636:1 gene:ENSMUSG00000082536 | 1.68 | Down | 0.010172 |
| ATP binding domain 4 | 1.68 | Down | 0.003197 |
| OTU domain containing 6B (Otud6b) | 1.68 | Down | 0.006434 |
| PHD finger protein 20-like 1 | 1.68 | Down | 0.005107 |
| Zinc finger protein 595 | 1.68 | Down | 0.005462 |
| Dihydrolipoamide dehydrogenase | 1.68 | Down | 0.013095 |
| Poly(A) polymerase gamma | 1.68 | Down | 0.005402 |
| Zinc finger CCCH type containing 11A (Zc3h11a) | 1.68 | Down | 0.005186 |
| Metal response element binding transcription factor 2 (Mtf2) | 1.68 | Down | 0.004011 |
| Family with sequence similarity 134, member B | 1.68 | Down | 0.007837 |
| Clathrin interactor 1 | 1.68 | Down | 0.001031 |
| Nuclear receptor subfamily 0, group B, member 2 | 1.68 | Up | 0.012632 |
| Integrator complex subunit 4 | 1.68 | Down | 0.002409 |
| O-linked N-acetylglucosamine (GlcNAc) transferase (UDP-N-acetylglucosamine:polypeptide-N-acetylglucosaminyl transferase) (Ogt | 1.68 | Down | 0.013744 |
| ncrna:snRNA chromosome:NCBIM37:6:6874118:6874279:-1 gene:ENSMUSG00000065770 | 1.68 | Up | 0.0406 |
| PREDICTED: Mus musculus similar to pol protein | 1.68 | Down | 0.003604 |
| Premature mRNA for mKIAA0856 protein | 1.68 | Down | 0.003573 |
| Transcribed locus | 1.68 | Down | 0.004739 |
| Eukaryotic translation initiation factor 3, subunit J (Eif3j) | 1.68 | Down | 0.007084 |
| Mus musculus, clone IMAGE:4242613 | 1.67 | Down | 0.003339 |
| SEC62 homolog (S. cerevisiae) | 1.67 | Down | 0.00288 |
| cdna:Genscan chromosome:NCBIM37:7:50252240:50254829:1 | 1.67 | Down | 0.025154 |
| LanC (bacterial lantibiotic synthetase component C)-like 2 (Lancl2) | 1.67 | Down | 0.002577 |
| G protein-coupled receptor 116 (Gpr116) | 1.67 | Down | 0.00516 |
| Breast cancer resistance protein 1 (Bcrp1) | 1.67 | Down | 0.008765 |
| A disintegrin and metallopeptidase domain 17 (Adam17) | 1.67 | Down | 0.001631 |
| C57BL/6J adipose differentiation-related protein | 1.67 | Down | 0.024131 |
| Cysteine and glycine-rich protein 3 (Csrp3) | 1.67 | Down | 0.017136 |
| Regulator of sex limited protein 1 | 1.67 | Down | 0.015429 |
| RIKEN cDNA A230046K03 gene | 1.67 | Down | 0.012313 |
| RIKEN cDNA B230339M05 gene | 1.67 | Down | 0.001877 |
| Ring finger protein 213 gene:ENSMUSG00000070327 | 1.67 | Down | 0.002363 |
| X-ray repair complementing defective repair in Chinese hamster cells 4 (Xrcc4) | 1.67 | Down | 0.015097 |
| Twinfilin, actin-binding protein, homolog 1 (Drosophila) | 1.67 | Down | 0.009714 |
| Solute carrier family 23 (nucleobase transporters), member 1 | 1.67 | Down | 0.008414 |
| Arylsulfatase K (Arsk) | 1.67 | Down | 0.012729 |
| Bcl-2-binding protein BIS (Bis) | 1.67 | Up | 0.009469 |
| Translocase of inner mitochondrial membrane 8 homolog a1 (yeast) | 1.67 | Up | 0.002531 |
| ATP-binding cassette, sub-family A (ABC1), member 8b (Abca8b) | 1.67 | Down | 0.016767 |
| Protein tyrosine phosphatase-like (proline instead of catalytic arginine), member a (Ptpla), transcript variant 1 | 1.67 | Up | 0.004155 |
| RIKEN cDNA 4931406C07 gene (4931406C07Rik) | 1.67 | Down | 0.003908 |
| Origin recognition complex, subunit 5-like (S. cerevisiae) | 1.67 | Down | 0.014753 |
| Protein phosphatase 2 (formerly 2A), regulatory subunit A (PR 65), beta isoform (Ppp2r1b), transcript variant 2 | 1.67 | Down | 0.00288 |
| Capping protein (actin filament) muscle Z-line, alpha 1 (Capza1) | 1.67 | Down | 0.004111 |
| Ubiquitin specific peptidase 47 | 1.67 | Down | 0.013785 |
| Claudin 1 | 1.67 | Down | 0.002037 |
| RIO kinase 3 (yeast) | 1.67 | Down | 0.005718 |
| CDNA clone MGC:198792 IMAGE:9054373 | 1.67 | Down | 0.041719 |
| MAP kinase-interacting serine/threonine kinase 2 | 1.67 | Up | 0.003462 |
| Kruppel-like factor 7 (ubiquitous) | 1.67 | Down | 0.002734 |
| Ubiquitin specific peptidase 40 | 1.67 | Down | 0.010813 |
| Of ZT2 gene encoding zinc finger protein 125 | 1.67 | Down | 0.008385 |
| Zinc finger RNA binding protein | 1.67 | Down | 0.002409 |
| Claudin 2 | 1.67 | Down | 0.003521 |
| Proteasome (prosome, macropain) 26S subunit, non-ATPase, 5 (Psmd5) | 1.67 | Down | 0.004655 |
| Large tumor suppressor 1 (Lats1) | 1.67 | Down | 0.000782 |
| Ras homolog gene family, member T1 | 1.67 | Down | 0.004462 |
| RMI1, RecQ mediated genome instability 1, homolog (S. cerevisiae) | 1.66 | Down | 0.018357 |
| CDNA clone IMAGE:3675158 | 1.66 | Down | 0.004505 |
| CDC16 cell division cycle 16 homolog (S. cerevisiae) (Cdc16) | 1.66 | Down | 0.002928 |
| Ubiquitin specific peptidase 9, X chromosome (Usp9x) | 1.66 | Down | 0.008897 |
| DEAD (Asp-Glu-Ala-Asp) box polypeptide 5 | 1.66 | Down | 0.002541 |
| RIKEN cDNA 4933409K07 gene | 1.66 | Down | 0.002254 |
| Phospholipase A2, activating protein | 1.66 | Down | 0.006898 |
| Retinoblastoma binding protein 9 | 1.66 | Down | 0.003462 |
| 3-phosphoadenosine 5-phosphosulfate synthase 1 (Papss1) | 1.66 | Down | 0.002682 |
| RIKEN cDNA 4933409K07 gene | 1.66 | Down | 0.002908 |
| RIKEN cDNA 4933409K07 gene | 1.66 | Down | 0.002908 |
| UDP-glucose ceramide glucosyltransferase-like 1 | 1.66 | Down | 0.002445 |
| Erbb2 interacting protein | 1.66 | Down | 0.006602 |
| Twinfilin, actin-binding protein, homolog 1 (Drosophila) | 1.66 | Down | 0.005588 |
| Solute carrier family 43, member 3 (Slc43a3) | 1.66 | Down | 0.00151 |
| Striatin, calmodulin binding protein (Strn) | 1.66 | Down | 0.002885 |
| Nucleolar protein 11 | 1.66 | Down | 0.003994 |
| Maltase-glucoamylase (Mgam) | 1.66 | Down | 0.004791 |
| Pyridine nucleotide-disulphide oxidoreductase domain 1 | 1.66 | Down | 0.003951 |
| gi|34538597|ref|NC_005089.1|:11613-11671, tRNA-Ser | 1.66 | Up | 0.008765 |
| Ring finger protein 13 (Rnf13), transcript variant 2 | 1.66 | Down | 0.010724 |
| predicted gene, EG433168 | 1.66 | Up | 0.022107 |
| Endothelial-specific receptor tyrosine kinase (Tek) | 1.66 | Down | 0.003274 |
| Matrin 3 | 1.66 | Down | 0.019699 |
| DNA segment, Chr 3, ERATO Doi 300, expressed (D3Ertd300e) | 1.65 | Down | 0.005579 |
| cdna:known chromosome:NCBIM37:2:98506704:98507458:-1 gene:ENSMUSG00000075014 | 1.65 | Up | 0.038235 |
| ATPase, H+ transporting, lysosomal V1 subunit C1 | 1.65 | Down | 0.003185 |
| ncrna:rRNA chromosome:NCBIM37:8:126125472:126125580:-1 gene:ENSMUSG00000075772 | 1.65 | Up | 0.017355 |
| ncrna:rRNA chromosome:NCBIM37:8:126125472:126125580:-1 gene:ENSMUSG00000075772 | 1.65 | Up | 0.017355 |
| Activating signal cointegrator 1 complex subunit 3 | 1.65 | Down | 0.007775 |
| E1A-like inhibitor of differentiation | 1.65 | Up | 0.004327 |
| JNK-associated leucine-zipper protein | 1.65 | Down | 0.014027 |
| DEAD (Asp-Glu-Ala-Asp) box polypeptide 5 | 1.65 | Down | 0.004521 |
| Transcription elongation factor A (SII) 1 | 1.65 | Down | 0.007565 |
| Tudor domain containing 7 (Tdrd7) | 1.65 | Down | 0.003462 |
| ATPase type 13A3 (Atp13a3), transcript variant 2 | 1.65 | Down | 0.00577 |
| Glutamic pyruvic transaminase, soluble | 1.65 | Up | 0.001085 |
| Core 1 synthase, glycoprotein-N-acetylgalactosamine 3-beta-galactosyltransferase, 1 | 1.65 | Down | 0.0082 |
| Exportin 4 (Xpo4) | 1.65 | Down | 0.002885 |
| MKIAA0678 protein | 1.65 | Down | 0.0243 |
| Transcription factor 12 | 1.65 | Down | 0.003573 |
| Transcribed locus, strongly similar to NP_599008.2 acyl-CoA thioesterase 4 [Mus musculus] | 1.65 | Up | 0.033534 |
| NF1GRP mRNA for neurofibromatosis type-1-GTPase activating-protein type IV | 1.65 | Down | 0.008737 |
| Nuclear receptor coactivator ASC-1 testis specific form mRNA, complete cds; alternatively sliced | 1.65 | Down | 0.002688 |
| Suppressor of hairy wing homolog 4 (Drosophila) (Suhw4) | 1.65 | Down | 0.009115 |
| Arginyltransferase 1 (Ate1), transcript variant 1 | 1.65 | Down | 0.00393 |
| INSIG-2 membrane protein | 1.65 | Down | 0.020991 |
| Threonyl-tRNA synthetase (Tars) | 1.65 | Down | 0.001631 |
| ncrna:snoRNA chromosome:NCBIM37:18:75161534:75161598:1 gene:ENSMUSG00000064844 | 1.65 | Up | 0.044162 |
| Exonuclease domain containing 1 (Exod1) | 1.65 | Down | 0.018151 |
| RIKEN cDNA 4932438A13 gene | 1.65 | Down | 0.029782 |
| Transient receptor potential phospholipase C interacting kinase | 1.65 | Down | 0.036449 |
| Retinoblastoma 1 (Rb1) | 1.65 | Down | 0.006622 |
| Sorting nexin 5 (Snx5) | 1.65 | Down | 0.009105 |
| RIKEN cDNA 4833432P19 gene | 1.65 | Down | 0.011134 |
| Family with sequence similarity 62, member B (Fam62b) | 1.65 | Down | 0.005523 |
| SUMO/sentrin specific peptidase 6 (Senp6) | 1.65 | Down | 0.003365 |
| Karyopherin (importin) alpha 2 (Kpna2) | 1.65 | Down | 0.004323 |
| Stromal antigen 2 (Stag2), transcript variant 1 | 1.65 | Down | 0.022072 |
| Family with sequence similarity 82, member A1 | 1.65 | Down | 0.002253 |
| TATA box binding protein (Tbp)-associated factor, RNA polymerase I, D (Taf1d), transcript variant 2 | 1.65 | Down | 0.02401 |
| HECT domain containing 1 (Hectd1) | 1.65 | Down | 0.003695 |
| Arginine/serine-rich coiled-coil 2 | 1.65 | Down | 0.00341 |
| Neuregulin 4 | 1.65 | Up | 0.018261 |
| Inter-alpha trypsin inhibitor, heavy chain 2 | 1.64 | Down | 0.005437 |
| Polybromo 1 | 1.64 | Down | 0.022917 |
| NC_005089 | 1.64 | Up | 0.022598 |
| Karyopherin (importin) alpha 2 (Kpna2) | 1.64 | Down | 0.00632 |
| Golgi phosphoprotein 3-like (Golph3l) | 1.64 | Down | 0.005062 |
| ArfGAP with RhoGAP domain, ankyrin repeat and PH domain 2 | 1.64 | Down | 0.013021 |
| Carbonic anhydrase 14 (Car14) | 1.64 | Up | 0.003228 |
| Ataxin 3 | 1.64 | Down | 0.007005 |
| BTAF1 RNA polymerase II, B-TFIID transcription factor-associated, (Mot1 homolog, S. cerevisiae) | 1.64 | Down | 0.004028 |
| Glutamate-cysteine ligase, catalytic subunit | 1.64 | Down | 0.00828 |
| Tripartite motif-containing 2 (Trim2) | 1.64 | Down | 0.011086 |
| Malate dehydrogenase 1, NAD (soluble) (Mdh1) | 1.64 | Down | 0.004434 |
| 5-3 exoribonuclease 2 | 1.64 | Down | 0.016424 |
| Nucleophosmin 1 (Npm1) | 1.64 | Down | 0.013908 |
| Zinc finger CCCH-type containing 15 (Zc3h15) | 1.64 | Down | 0.01331 |
| Family with sequence similarity 178, member A | 1.64 | Down | 0.013922 |
| Exocyst complex component 3 | 1.64 | Down | 0.005825 |
| PREDICTED: Mus musculus hypothetical protein LOC100039986 | 1.64 | Down | 0.002598 |
| Transcription factor B2, mitochondrial (Tfb2m), nuclear gene encoding mitochondrial protein | 1.64 | Down | 0.013191 |
| Collagen, type IV, alpha 3 (Goodpasture antigen) binding protein | 1.64 | Down | 0.003908 |
| Janus kinase 2 (Jak2), transcript variant 1 | 1.64 | Down | 0.019484 |
| Transcription factor EC (Tcfec) | 1.64 | Down | 0.004268 |
| RIKEN cDNA D330038O06 gene | 1.64 | Down | 0.002724 |
| Peptidylprolyl isomerase (cyclophilin)-like 4 (Ppil4) | 1.64 | Down | 0.004644 |
| Complement component 9 | 1.64 | Down | 0.014033 |
| RAN binding protein 2 (Ranbp2) | 1.64 | Down | 0.01219 |
| Bone morphogenic protein receptor, type II (serine/threonine kinase) (Bmpr2) | 1.64 | Down | 0.003149 |
| Rho GTPase activating protein 12 | 1.64 | Down | 0.00228 |
| Endoplasmic reticulum aminopeptidase 1 (Erap1) | 1.64 | Down | 0.012847 |
| Transcription factor B2, mitochondrial (Tfb2m), nuclear gene encoding mitochondrial protein | 1.64 | Down | 0.012785 |
| DNA segment, Chr 14, ERATO Doi 449, expressed | 1.64 | Down | 0.000561 |
| predicted gene, EG665570 | 1.64 | Up | 0.010174 |
| PREDICTED: Mus musculus RIKEN cDNA 4930432O21 gene | 1.64 | Down | 0.008824 |
| RIKEN cDNA 9530009G21 gene | 1.64 | Down | 0.00341 |
| Membrane protein, palmitoylated 6 (MAGUK p55 subfamily member 6) (Mpp6) | 1.64 | Down | 0.003191 |
| ncrna:misc_RNA chromosome:NCBIM37:3:88497852:88497979:1 gene:ENSMUSG00000077300 | 1.64 | Up | 0.015265 |
| Translocase of inner mitochondrial membrane 8 homolog a1 (yeast) | 1.64 | Up | 0.002553 |
| SFRS12-interacting protein 1 | 1.64 | Down | 0.010504 |
| DNA segment, Chr 14, ERATO Doi 449, expressed | 1.64 | Down | 0.000561 |
| RING finger protein (Fxy2) | 1.64 | Down | 0.001675 |
| Ran binding protein 5 | 1.63 | Down | 0.004111 |
| RNA binding motif protein 5 (Rbm5) | 1.63 | Down | 0.003573 |
| RIKEN cDNA 2310035C23 gene, transcript variant 2 | 1.63 | Down | 0.003691 |
| Ribosomal protein S4, X-linked | 1.63 | Down | 0.012743 |
| RIKEN cDNA 5730427N09 gene (5730427N09Rik) | 1.63 | Down | 0.005467 |
| Vacuolar protein sorting 37A (yeast) | 1.63 | Down | 0.004067 |
| Phosphatidylethanolamine-binding protein (Pebp) | 1.63 | Up | 0.009763 |
| Adaptor protein, phosphotyrosine interaction, PH domain and leucine zipper containing 2 (Appl2) | 1.63 | Down | 0.003089 |
| Forty-two-three domain containing 1 (Fyttd1) | 1.63 | Down | 0.006321 |
| Adaptor molecule SRCASM (Srcasm) | 1.63 | Down | 0.002161 |
| NFATx | 1.63 | Down | 0.002107 |
| Fumarate hydratase 1 | 1.63 | Down | 0.006485 |
| Mitogen-activated protein kinase 9 | 1.63 | Down | 0.003072 |
| Nucleophosmin 1 (Npm1) | 1.63 | Down | 0.046887 |
| PREDICTED: Mus musculus RIKEN cDNA 6820431F20 gene | 1.63 | Down | 0.005598 |
| small nucleolar RNA, C/D box 82 | 1.63 | Up | 0.016633 |
| AVL9 homolog (S. cerevisiase) | 1.63 | Down | 0.007143 |
| Guanine nucleotide binding protein, alpha 13 | 1.63 | Down | 0.002499 |
| RIKEN cDNA 5730427N09 gene | 1.63 | Down | 0.004017 |
| Ectonucleotide pyrophosphatase/phosphodiesterase 4 | 1.63 | Down | 0.003229 |
| Elongation protein 2 homolog (S. cerevisiae) | 1.63 | Down | 0.005243 |
| Hook homolog 1 (Drosophila) (Hook1) | 1.63 | Down | 0.032173 |
| Replication factor C (activator 1) 4 (Rfc4) | 1.63 | Down | 0.010637 |
| RIKEN cDNA 4933426I21 gene | 1.63 | Down | 0.017532 |
| Chaperonin containing Tcp1, subunit 3 (gamma) (Cct3) | 1.63 | Down | 0.003946 |
| Ubiquitin specific peptidase 14 | 1.63 | Down | 0.006774 |
| predicted gene, OTTMUSG00000000777 | 1.63 | Up | 0.002107 |
| Zinc finger, MYND domain containing 11 | 1.63 | Down | 0.001838 |
| PREDICTED: Mus musculus predicted gene, EG620480 | 1.63 | Up | 0.006637 |
| Syntaxin binding protein 3A (Stxbp3a) | 1.63 | Down | 0.015343 |
| Masp3 mRNA for MBL-associated serine protease-3 | 1.63 | Down | 0.002923 |
| Lipase, hepatic | 1.63 | Down | 0.002863 |
| USO1 homolog, vesicle docking protein (yeast) (Uso1) | 1.63 | Down | 0.002409 |
| Expressed sequence AI182371 | 1.63 | Down | 0.003616 |
| DnaJ (Hsp40) homolog, subfamily B, member 9 | 1.63 | Up | 0.002927 |
| DEAD (Asp-Glu-Ala-Asp) box polypeptide 50 (Ddx50) | 1.63 | Down | 0.007837 |
| ATPase, H+ transporting, lysosomal V1 subunit A | 1.63 | Down | 0.002171 |
| cdna:pseudogene chromosome:NCBIM37:8:43332189:43332805:1 gene:ENSMUSG00000053038 | 1.63 | Up | 0.003595 |
| Transmembrane protein 60 | 1.63 | Up | 0.002704 |
| Methyltransferase like 4 | 1.63 | Down | 0.037272 |
| Syntaxin 12 (Stx12) | 1.63 | Down | 0.012818 |
| Coatomer protein complex subunit alpha | 1.63 | Down | 0.001844 |
| PRP4 pre-mRNA processing factor 4 homolog B (yeast) (Prpf4b) | 1.63 | Down | 0.003473 |
| Serine (or cysteine) peptidase inhibitor, clade A, member 3K | 1.63 | Down | 0.012767 |
| Aminoacylase 1 | 1.62 | Up | 0.002908 |
| Dihydrouridine synthase 4-like (S. cerevisiae) (Dus4l) | 1.62 | Down | 0.018762 |
| MRNA of enhancer-trap-locus 1 | 1.62 | Down | 0.015308 |
| Ectonucleoside triphosphate diphosphohydrolase 4 | 1.62 | Down | 0.000408 |
| Palmdelphin (PALMD gene) | 1.62 | Down | 0.008261 |
| Hemolytic complement | 1.62 | Down | 0.013755 |
| predicted gene, 100042289 | 1.62 | Up | 0.008415 |
| DNA segment, Chr 14, ERATO Doi 449, expressed | 1.62 | Down | 0.000489 |
| Calmodulin 1 (Calm1) | 1.62 | Down | 0.002577 |
| Karyopherin (importin) alpha 4 | 1.62 | Down | 0.003887 |
| Formin 1 (Fmn1), transcript variant 1 | 1.62 | Down | 0.03462 |
| Expressed sequence AU014645 | 1.62 | Down | 0.004067 |
| Family with sequence similarity 179, member B | 1.62 | Down | 0.009877 |
| Chaperonin containing Tcp1, subunit 6a (zeta) | 1.62 | Down | 0.002724 |
| RIKEN cDNA A630047E20 gene | 1.62 | Down | 0.002681 |
| Intraflagellar transport 52 homolog (Chlamydomonas) | 1.62 | Down | 0.003401 |
| UBX domain protein 2A (Ubxn2a) | 1.62 | Down | 0.00151 |
| Interferon activated gene 204 | 1.62 | Down | 0.013862 |
| Tubulin, gamma complex associated protein 5 (Tubgcp5) | 1.62 | Down | 0.002928 |
| Putative transcription factor ZNF131 (Znf131) | 1.62 | Down | 0.002577 |
| ncrna:snRNA chromosome:NCBIM37:3:86188169:86188275:1 gene:ENSMUSG00000064867 | 1.62 | Up | 0.040594 |
| T-cell specific GTPase | 1.62 | Down | 0.008019 |
| Phosphodiesterase 4B, cAMP specific | 1.62 | Down | 0.006653 |
| RIKEN cDNA 1110059E24 gene | 1.62 | Down | 0.01326 |
| G2/M-phase specific E3 ubiquitin ligase (G2e3) | 1.62 | Down | 0.005692 |
| Ubiquitin specific peptidase 32 (Usp32) | 1.62 | Down | 0.00632 |
| Predicted gene, EG668668 | 1.62 | Down | 0.019848 |
| ncrna:snRNA chromosome:NCBIM37:9:41147519:41147624:-1 gene:ENSMUSG00000064909 | 1.62 | Down | 0.028908 |
| Zinc finger protein 148 | 1.62 | Down | 0.005523 |
| RIKEN cDNA 5033414K04 gene | 1.62 | Down | 0.002902 |
| CDNA sequence BC003331 (BC003331), transcript variant 1 | 1.62 | Down | 0.034563 |
| CD2-associated protein (CD2AP gene) | 1.62 | Down | 0.00641 |
| Jumonji domain containing 1A | 1.62 | Down | 0.003348 |
| Activated leukocyte cell adhesion molecule | 1.62 | Down | 0.005784 |
| PREDICTED: Mus musculus A kinase (PRKA) anchor protein 11 (Akap11) | 1.62 | Down | 0.011169 |
| Ring finger protein 213 gene:ENSMUSG00000070327 | 1.62 | Down | 0.003119 |
| Transmembrane protein 144 (Tmem144) | 1.62 | Down | 0.002561 |
| PREDICTED: Mus musculus RIKEN cDNA D230012E17 gene (D230012E17Rik) | 1.62 | Down | 0.008779 |
| Rb1cc1 mRNA for transcription factor | 1.62 | Down | 0.042523 |
| Membrane protein NBR1 (Nbr1) | 1.62 | Down | 0.002908 |
| EPM2A (laforin) interacting protein 1 | 1.62 | Down | 0.011289 |
| Complement component factor h (Cfh) | 1.62 | Down | 0.036184 |
| General transcription factor IIIC, polypeptide 3 | 1.62 | Down | 0.028875 |
| RIKEN cDNA 9530058B02 gene | 1.62 | Up | 0.002103 |
| Reticuloendotheliosis oncogene (Rel) | 1.62 | Down | 0.003928 |
| Predicted gene, EG665378 (EG665378) | 1.62 | Down | 0.002577 |
| UTP6, small subunit (SSU) processome component, homolog (yeast) ( | 1.62 | Down | 0.00353 |
| Elongation factor RNA polymerase II 2 (Ell2) | 1.61 | Down | 0.003363 |
| Ubiquitin-conjugating enzyme E2 variant 2 | 1.61 | Down | 0.018343 |
| Interleukin 13 receptor, alpha 1 (Il13ra1) | 1.61 | Down | 0.048525 |
| Karyopherin (importin) alpha 2 (Kpna2) | 1.61 | Down | 0.005367 |
| RIKEN cDNA D630014A15 gene | 1.61 | Down | 0.003936 |
| RIKEN cDNA 1810030O07 gene | 1.61 | Down | 0.009667 |
| RIKEN cDNA A130022J15 gene | 1.61 | Down | 0.012885 |
| Integrin beta 3 binding protein (beta3-endonexin) (Itgb3bp) | 1.61 | Down | 0.002171 |
| Pentatricopeptide repeat domain 3 (Ptcd3) | 1.61 | Down | 0.03653 |
| Formin binding protein 1-like (Fnbp1l), transcript variant 2 | 1.61 | Down | 0.004521 |
| Ribosomal protein S4, X-linked | 1.61 | Down | 0.01498 |
| cDNA sequence AY036118 | 1.61 | Up | 0.02762 |
| Transmembrane protein 14A | 1.61 | Up | 0.001877 |
| PDLIM1 interacting kinase 1 like (Pdik1l) | 1.61 | Down | 0.000561 |
| KRIT1, ankyrin repeat containing (Krit1) | 1.61 | Down | 0.002279 |
| Suppressor of zeste 12 homolog (Drosophila) | 1.61 | Down | 0.005186 |
| DEAD (Asp-Glu-Ala-Asp) box polypeptide 6 | 1.61 | Down | 0.003708 |
| Hermansky-Pudlak syndrome 3 homolog (human) | 1.61 | Down | 0.003119 |
| XY body protein (Xybp) | 1.61 | Down | 0.005888 |
| SAPS domain family, member 3 (Saps3) | 1.61 | Down | 0.006125 |
| Kip1 C-terminus interacting protein-2 (Kic2) | 1.61 | Down | 0.008129 |
| SLU7 splicing factor homolog (S. cerevisiae) (Slu7), transcript variant 1 | 1.61 | Down | 0.027134 |
| SMG1 homolog, phosphatidylinositol 3-kinase-related kinase (C. elegans) (Smg1) | 1.61 | Down | 0.011871 |
| MKIAA0277 protein | 1.61 | Down | 0.01302 |
| Helicase (DNA) B (Helb) | 1.61 | Down | 0.002725 |
| Predicted gene, EG240549 | 1.61 | Down | 0.002256 |
| 5-azacytidine induced gene 2 | 1.61 | Down | 0.002724 |
| Phosphatidylinositol glycan anchor biosynthesis, class N | 1.61 | Down | 0.005728 |
| Armadillo repeat containing, X-linked 3 (Armcx3) | 1.61 | Down | 0.009909 |
| N-myristoyltransferase 1 | 1.61 | Down | 0.002005 |
| RIKEN cDNA C330023M02 gene | 1.61 | Down | 0.002409 |
| Tropomyosin 4 (Tpm4) | 1.61 | Down | 0.006263 |
| cdna:Genscan chromosome:NCBIM37:9:104051743:104140870:-1 | 1.61 | Down | 0.019228 |
| Calpain 2 (Capn2) | 1.61 | Down | 0.001631 |
| Inhibin beta-A (Inhba) | 1.61 | Down | 0.006545 |
| Pwcr1 mRNA, complete sequence | 1.61 | Up | 0.010395 |
| Proteasome (prosome, macropain) 26S subunit, non-ATPase, 12 | 1.61 | Down | 0.004126 |
| V-ATPase E2 subunit | 1.61 | Down | 0.005524 |
| Ubiquitin protein ligase E3 component n-recognin 1 (Ubr1) | 1.61 | Down | 0.003525 |
| Heat shock protein 4 | 1.61 | Down | 0.002908 |
| GTP-binding protein 10 (putative) | 1.61 | Down | 0.007141 |
| RIKEN cDNA 4732418C07 gene | 1.61 | Down | 0.002598 |
| ncrna:rRNA chromosome:NCBIM37:8:126108435:126108553:-1 gene:ENSMUSG00000075856 | 1.61 | Up | 0.017382 |
| Glycosyltransferase-like domain containing 1 | 1.61 | Down | 0.036184 |
| Nucleophosmin 1 (Npm1) | 1.61 | Down | 0.027332 |
| Ring finger protein 160 | 1.61 | Down | 0.006148 |
| RIKEN cDNA A530094I17 gene | 1.61 | Down | 0.018353 |
| ribonuclease P RNA-like 1 | 1.61 | Up | 0.007375 |
| Eukaryotic translation initiation factor 2, subunit 1 alpha | 1.6 | Down | 0.010927 |
| MKIAA0137 protein | 1.6 | Down | 0.00247 |
| Phosphatidylinositol 3-kinase, C2 domain containing, alpha polypeptide (Pik3c2a) | 1.6 | Down | 0.018259 |
| RIKEN cDNA 4933411K16 gene | 1.6 | Down | 0.026355 |
| Motile sperm domain containing 1 (Mospd1) | 1.6 | Down | 0.027371 |
| SMAD specific E3 ubiquitin protein ligase 2 | 1.6 | Down | 0.003688 |
| Dehydrogenase/reductase (SDR family) member 11 | 1.6 | Up | 0.0037 |
| Oxysterol binding protein-like 1A | 1.6 | Down | 0.005034 |
| AT rich interactive domain 2 (ARID, RFX-like) | 1.6 | Down | 0.003276 |
| Integrator complex subunit 6 (Ints6) | 1.6 | Down | 0.001965 |
| RIKEN cDNA 1810027O10 gene | 1.6 | Up | 0.003312 |
| Aldehyde dehydrogenase 1 family, member B1 (Aldh1b1), nuclear gene encoding mitochondrial protein | 1.6 | Up | 0.025229 |
| MKIAA0678 protein | 1.6 | Down | 0.012005 |
| component of oligomeric golgi complex 5 | 1.6 | Down | 0.008093 |
| ncrna:misc_RNA chromosome:NCBIM37:10:74905365:74905688:1 gene:ENSMUSG00000084622 | 1.6 | Down | 0.042989 |
| predicted gene, EG666579 | 1.6 | Up | 0.012736 |
| Anaphase promoting complex subunit 4 (Anapc4) | 1.6 | Down | 0.005928 |
| Low density lipoprotein receptor-related protein 6 (Lrp6) | 1.6 | Down | 0.004074 |
| IQ calmodulin-binding motif containing 1 | 1.6 | Down | 0.005689 |
| IK cytokine | 1.6 | Down | 0.008005 |
| Formin binding protein 4 (Fnbp4) | 1.6 | Down | 0.002037 |
| Zinc finger, FYVE domain containing 9 | 1.6 | Down | 0.002216 |
| Thyroid hormone receptor interactor 12 | 1.6 | Down | 0.005651 |
| DEAD (Asp-Glu-Ala-Asp) box polypeptide 46 (Ddx46) | 1.6 | Down | 0.005686 |
| Ring finger and SPRY domain containing 1 (Rspry1) | 1.6 | Down | 0.001085 |
| Mitochondrial ribosomal protein L1 | 1.6 | Down | 0.01331 |
| Nuclear factor of activated T-cells 5 (Nfat5), transcript variant a | 1.6 | Down | 0.008424 |
| Necdin (Ndn) | 1.6 | Up | 0.002902 |
| N-myc (and STAT) interactor | 1.6 | Down | 0.004074 |
| Neutral sphingomyelinase (N-SMase) activation associated factor (Nsmaf) | 1.6 | Down | 0.007856 |
| Ring finger protein, LIM domain interacting | 1.6 | Down | 0.005467 |
| Aminolevulinic acid synthase 1 (Alas1) | 1.6 | Up | 0.032683 |
| Spermatid perinuclear RNA binding protein | 1.6 | Down | 0.016069 |
| Synaptotagmin I (Syt1) | 1.6 | Down | 0.011353 |
| Ring finger protein 160 | 1.6 | Down | 0.01835 |
| cdna:pseudogene chromosome:NCBIM37:12:7887289:7890451:-1 gene:ENSMUSG00000071430 | 1.6 | Down | 0.009804 |
| Ataxin 7-like 3 (Atxn7l3), transcript variant 2 | 1.6 | Up | 0.003337 |
| Rosbin, round spermatid basic protein 1 | 1.6 | Down | 0.008041 |
| YTH domain containing 1 | 1.6 | Down | 0.005673 |
| DNA-damage inducible protein 2 (Ddi2) | 1.6 | Down | 0.002553 |
| Karyopherin (importin) alpha 1 | 1.6 | Down | 0.006607 |
| RIKEN cDNA 1110058L19 gene | 1.6 | Up | 0.008953 |
| Betaine-homocysteine methyltransferase | 1.6 | Down | 0.002262 |
| Mitogen-activated protein kinase kinase kinase kinase 5 | 1.59 | Down | 0.00643 |
| RIKEN cDNA 2310001H12 gene | 1.59 | Down | 0.015221 |
| Betaine-homocysteine methyltransferase | 1.59 | Down | 0.002445 |
| Uracil phosphoribosyltransferase (FUR1) homolog (S. cerevisiae) | 1.59 | Down | 0.021958 |
| Protein phosphatase 1K (PP2C domain containing) | 1.59 | Down | 0.014321 |
| RIKEN cDNA 1200003I07 gene, transcript variant 2 | 1.59 | Down | 0.003009 |
| Cyclin-dependent kinase-like 5 (Cdkl5) | 1.59 | Down | 0.014462 |
| Gamma-aminobutyric acid (GABA-A) receptor, subunit alpha 3 (Gabra3) | 1.59 | Down | 0.010927 |
| Protein phosphatase 1 binding protein PTG | 1.59 | Down | 0.006932 |
| Karyopherin (importin) beta 1 | 1.59 | Down | 0.003197 |
| Dicer1, Dcr-1 homolog (Drosophila) | 1.59 | Down | 0.008001 |
| Translocase of outer mitochondrial membrane 70 homolog A (yeast) | 1.59 | Down | 0.01235 |
| Ribosomal protein S4, X-linked | 1.59 | Down | 0.018911 |
| RIKEN cDNA 1600002H07 gene (1600002H07Rik) | 1.59 | Up | 0.04707 |
| SET domain, bifurcated 2 | 1.59 | Down | 0.024687 |
| Putative E1-E2 ATPase | 1.59 | Down | 0.01643 |
| Zinc finger protein 280C (Zfp280c) | 1.59 | Down | 0.019843 |
| Fragile X mental retardation protein FMRP mRNA, complete cds, alternatively spliced | 1.59 | Down | 0.009586 |
| La ribonucleoprotein domain family, member 5 | 1.59 | Down | 0.004191 |
| Apolipoprotein L 7a | 1.59 | Down | 0.010867 |
| Keratin 18 (Krt18) | 1.59 | Down | 0.00404 |
| Vacuolar protein sorting 36 (yeast) | 1.59 | Down | 0.002808 |
| Olfactomedin 3 (Olfm3), transcript variant A | 1.59 | Down | 0.025626 |
| E1 protein | 1.59 | Down | 0.003149 |
| small nucleolar RNA, C/D box 34 | 1.59 | Up | 0.038479 |
| Cleavage and polyadenylation specificity factor 3 | 1.59 | Down | 0.002254 |
| LIM domain containing preferred translocation partner in lipoma | 1.59 | Down | 0.002808 |
| Phosphatidylinositol 3-kinase, regulatory subunit, polypeptide 1 (p85 alpha) | 1.59 | Down | 0.003695 |
| Amylase 1, salivary | 1.59 | Down | 0.026592 |
| Coiled-coil-helix-coiled-coil-helix domain containing 1 (Chchd1) | 1.59 | Up | 0.003552 |
| Non-SMC element 2 homolog (MMS21, S. cerevisiae) (Nsmce2) | 1.59 | Down | 0.008301 |
| MACRO domain containing 1 (Macrod1) | 1.59 | Up | 0.001631 |
| RAN binding protein 6 | 1.59 | Down | 0.006374 |
| Dynein light chain Tctex-type 3 | 1.59 | Down | 0.011205 |
| Kynurenine 3-monooxygenase (kynurenine 3-hydroxylase) (Kmo) | 1.59 | Down | 0.00319 |
| GTP-binding protein 10 (putative) | 1.59 | Down | 0.010366 |
| Anaphase promoting complex subunit 1 (Anapc1) | 1.59 | Down | 0.004327 |
| PREDICTED: Mus musculus Bardet-Biedl syndrome 10 (human), transcript variant 1 (Bbs10) | 1.59 | Down | 0.003646 |
| Transforming growth factor, beta receptor I (Tgfbr1) | 1.59 | Down | 0.030458 |
| MKIAA4072 protein | 1.59 | Down | 0.016127 |
| DnaJ (Hsp40) homolog, subfamily B, member 1 | 1.59 | Up | 0.017751 |
| F-box protein 11 | 1.59 | Down | 0.004559 |
| CAS1 domain containing 1 | 1.59 | Down | 0.032035 |
| Nuclear receptor subfamily 3, group C, member 2 (Nr3c2) | 1.59 | Down | 0.008469 |
| Rho guanine nucleotide exchange factor (GEF) 3 | 1.58 | Down | 0.003211 |
| PRA1 domain family 2 (Praf2) | 1.58 | Up | 0.005086 |
| ncrna:miRNA chromosome:NCBIM37:13:8920582:8920684:-1 gene:ENSMUSG00000076030 | 1.58 | Up | 0.001844 |
| Ubiquitin-like modifier activating enzyme 6 | 1.58 | Down | 0.024099 |
| La ribonucleoprotein domain family, member 4 (Larp4), transcript variant 2 | 1.58 | Down | 0.006044 |
| Sorting nexin 14 | 1.58 | Down | 0.018261 |
| PHD finger protein 6 | 1.58 | Down | 0.01396 |
| DEAD/H (Asp-Glu-Ala-Asp/His) box polypeptide 3, X-linked (Ddx3x) | 1.58 | Down | 0.01069 |
| Desmocollin 2 | 1.58 | Down | 0.019722 |
| RIKEN cDNA 2310061J03 gene | 1.58 | Down | 0.003908 |
| Zinc finger protein 68 | 1.58 | Down | 0.007592 |
| Phosphatidylethanolamine-binding protein (Pebp) | 1.58 | Up | 0.003119 |
|  | 1.58 | Down | 0.01503 |
| predicted gene, EG620782 | 1.58 | Up | 0.022047 |
| Mitochondrial poly(A) polymerase (Mtpap), nuclear gene encoding mitochondrial protein | 1.58 | Down | 0.005145 |
| Proteasome (prosome, macropain) subunit, alpha type 1 | 1.58 | Down | 0.017729 |
| RIKEN cDNA 1810007M14 gene | 1.58 | Down | 0.003119 |
| Notum pectinacetylesterase homolog (Drosophila) | 1.58 | Up | 0.023998 |
| TNF receptor-associated factor 6 (Traf6) | 1.58 | Down | 0.004283 |
| CDNA clone IMAGE:40092312 | 1.58 | Down | 0.009373 |
| Synaptosomal-associated protein 29 | 1.58 | Down | 0.004017 |
| Eukaryotic translation initiation factor 4E binding protein 3 | 1.58 | Down | 0.01495 |
| LINE L1 ASL1/AK129128 fusion | 1.58 | Down | 0.006066 |
| Basic, immunoglobulin-like variable motif-containing protein (Bivm) | 1.58 | Down | 0.019598 |
| Mixed lineage kinase domain-like | 1.58 | Down | 0.039538 |
| murinoglobulin, pseudogene 1 | 1.58 | Down | 0.013111 |
| Coiled-coil domain containing 47 (Ccdc47) | 1.58 | Down | 0.024924 |
| Eukaryotic translation elongation factor 1 alpha 1 | 1.58 | Down | 0.002955 |
| Ethanolamine kinase 2 | 1.58 | Up | 0.010471 |
| Importin 11 | 1.58 | Down | 0.030868 |
| RIKEN cDNA 3110001I22 gene | 1.58 | Down | 0.001921 |
| Immunoglobulin superfamily, member 5 (Igsf5) | 1.58 | Down | 0.001838 |
| Sequestosome 1 | 1.58 | Down | 0.003134 |
| G-protein signalling modulator 2 (AGS3-like, C. elegans) (Gpsm2) | 1.58 | Down | 0.004644 |
| Ubiquitin specific peptidase 46 (Usp46) | 1.58 | Down | 0.003964 |
| Nuclear FMRP interacting protein 1 (Nufip1) | 1.58 | Down | 0.003965 |
| Jumonji C domain-containing histone demethylase 1 homolog D (S. cerevisiae) | 1.58 | Down | 0.005692 |
| Mannose-binding lectin (protein C) 2 | 1.58 | Up | 0.001631 |
| Ring finger protein 213 | 1.58 | Down | 0.007171 |
| Protein tyrosine phosphatase, receptor type, K | 1.58 | Down | 0.002254 |
| Praja1, RING-H2 motif containing | 1.58 | Down | 0.001714 |
| Adaptor-related protein complex 3, mu 1 subunit | 1.58 | Down | 0.020061 |
| RIKEN cDNA 5730427N09 gene | 1.58 | Down | 0.003647 |
| U2 small nuclear RNA | 1.58 | Up | 0.032455 |
| Ectonucleotide pyrophosphatase/phosphodiesterase 1 allotype b (Enpp1) mRNA, Enpp1-b allele | 1.58 | Down | 0.003661 |
| RIKEN cDNA 2900010J23 gene | 1.58 | Up | 0.002724 |
| Tax1 (human T-cell leukemia virus type I) binding protein 3 (Tax1bp3) | 1.57 | Up | 0.007565 |
| Chaperonin containing Tcp1, subunit 2 (beta) | 1.57 | Down | 0.002671 |
| Co-chaperone mt-GrpE#2 mRNA, nuclear gene encoding mitochondrial protein | 1.57 | Down | 0.01085 |
| Nuclear receptor subfamily 1, group D, member 2 (Nr1d2) | 1.57 | Down | 0.016855 |
| RIKEN cDNA C330011K17 gene | 1.57 | Down | 0.007044 |
| Ring finger protein 160 | 1.57 | Down | 0.03262 |
| ATP-binding cassette, sub-family B (MDR/TAP), member 4 (Abcb4) | 1.57 | Down | 0.002908 |
| Ankyrin repeat and IBR domain containing 1 | 1.57 | Down | 0.001744 |
| Tryptophan 2,3-dioxygenase | 1.57 | Down | 0.007515 |
| Pericentriolar material gene 1 protein (Pcm1) and pericentriolar material gene 1 protein (Pcm1) mRNAs | 1.57 | Down | 0.01284 |
| ncrna:snRNA chromosome:NCBIM37:3:40796615:40796772:1 gene:ENSMUSG00000064927 | 1.57 | Down | 0.00643 |
| MKIAA0678 protein | 1.57 | Down | 0.016693 |
| cdna:known chromosome:NCBIM37:12:88985196:88985417:-1 gene:ENSMUSG00000072910 | 1.57 | Up | 0.011252 |
| cdna:Genscan chromosome:NCBIM37:9:104051743:104140870:-1 | 1.57 | Down | 0.037802 |
| 3-phosphoadenosine 5-phosphosulfate synthase 2 (Papss2) | 1.57 | Down | 0.009888 |
| OTU domain containing 1 | 1.57 | Up | 0.009229 |
| CTD (carboxy-terminal domain, RNA polymerase II, polypeptide A) small phosphatase like 2 (Ctdspl2) | 1.57 | Down | 0.017158 |
| Zinc finger protein 281 | 1.57 | Down | 0.003096 |
| ncrna:snoRNA chromosome:NCBIM37:2:26766781:26766854:1 gene:ENSMUSG00000065258 | 1.57 | Up | 0.002656 |
| Pleckstrin and Sec7 domain containing 3 | 1.57 | Down | 0.002923 |
| NLR family, pyrin domain containing 6 | 1.57 | Down | 0.002403 |
| FGFR1 oncogene partner 2 | 1.57 | Down | 0.002254 |
| MAK16 homolog (S. cerevisiae) | 1.57 | Down | 0.0042 |
| RAS-homolog enriched in brain (Rheb) | 1.57 | Up | 0.002908 |
| cdna:Genscan chromosome:NCBIM37:8:35384870:35389099:-1 | 1.57 | Up | 0.034398 |
| CCAAT/enhancer binding protein (C/EBP), beta (Cebpb) | 1.57 | Up | 0.00785 |
| Ankyrin repeat and LEM domain containing 2 (Ankle2) | 1.57 | Down | 0.003274 |
| Replication protein A3 | 1.57 | Up | 0.003087 |
| WW domain containing adaptor with coiled-coil (Wac) | 1.57 | Down | 0.002902 |
| nicotinamide nucleotide transhydrogenase | 1.57 | Up | 0.001631 |
| Sorbin and SH3 domain containing 2 | 1.57 | Down | 0.002012 |
| Heat shock protein 1 (chaperonin 10) | 1.57 | Up | 0.002353 |
| Mediator complex subunit 23 (Med23) | 1.57 | Down | 0.002671 |
| Neurotrophic tyrosine kinase, receptor, type 2 (Ntrk2), transcript variant 2 | 1.57 | Up | 0.009638 |
| Cardiolipin synthase 1 (Crls1), transcript variant 2 | 1.57 | Up | 0.002267 |
| 5-3 exoribonuclease 1 (Xrn1) | 1.57 | Down | 0.006805 |
| Cytochrome P450, family 2, subfamily c, polypeptide 67 | 1.57 | Down | 0.041459 |
| Complement component (3b/4b) receptor 1-like | 1.57 | Up | 0.040789 |
| Guanylate binding protein 2 | 1.57 | Down | 0.01746 |
| RIKEN cDNA 2010309E21 gene | 1.57 | Down | 0.014266 |
| Ring finger protein 4 | 1.57 | Down | 0.002561 |
| CDNA clone MGC:198792 | 1.57 | Down | 0.026661 |
| Tax1 (human T-cell leukemia virus type I) binding protein 3 (Tax1bp3) | 1.57 | Up | 0.007338 |
| Serine/threonine kinase 35 | 1.57 | Up | 0.002425 |
| Zinc finger protein 277 (Zfp277), transcript variant 2 | 1.57 | Down | 0.01078 |
| Yamaguchi sarcoma viral (v-yes-1) oncogene homolog | 1.57 | Down | 0.002653 |
| Zinc finger and BTB domain containing 26 | 1.57 | Down | 0.002254 |
| Transducin (beta)-like 1 X-linked (Tbl1x) | 1.56 | Down | 0.002961 |
| Glycerate kinase | 1.56 | Up | 0.007368 |
| cdna:pseudogene chromosome:NCBIM37:2:161080192:161081193:-1 gene:ENSMUSG00000082603 | 1.56 | Up | 0.022878 |
| KLRAQ motif containing 1 (Klraq1) | 1.56 | Down | 0.001838 |
| Structural maintenance of chromosomes 5 (Smc5) | 1.56 | Down | 0.022707 |
| Nuclear VCP-like | 1.56 | Down | 0.004644 |
| PREDICTED: Mus musculus THAP domain containing 6 (Thap6) | 1.56 | Down | 0.003595 |
| Centrosome and spindle pole associated protein 1 | 1.56 | Down | 0.01127 |
| CDNA clone MGC:198792 IMAGE:9054373 | 1.56 | Down | 0.039527 |
| 11-zinc-finger transcription factor (CTCF) | 1.56 | Down | 0.003212 |
| Cell division cycle 5-like (S. pombe) (Cdc5l) | 1.56 | Down | 0.00606 |
| Cell division cycle 5-like (S. pombe) (Cdc5l) | 1.56 | Down | 0.00606 |
| PREDICTED: Mus musculus Ras association (RalGDS/AF-6) and pleckstrin homology domains 1 (Raph1) | 1.56 | Down | 0.0019 |
| DNA segment, Chr 1, ERATO Doi 622, expressed | 1.56 | Up | 0.002171 |
| Multiple EGF-like-domains 9 | 1.56 | Down | 0.002331 |
| Ring finger protein 170 | 1.56 | Down | 0.003879 |
| Zinc finger with KRAB and SCAN domains 1 (Zkscan1), transcript variant 1 | 1.56 | Down | 0.014181 |
| RIKEN cDNA 4833420G17 gene | 1.56 | Down | 0.005254 |
| Aldo-keto reductase family 1, member D1 (Akr1d1) | 1.56 | Down | 0.010164 |
| Cathepsin C (Ctsc) | 1.56 | Down | 0.002928 |
| Family with sequence similarity 108, member A (Fam108a) | 1.56 | Up | 0.003573 |
| Steroid 5 alpha-reductase 2 (Srd5a2) | 1.56 | Down | 0.003009 |
| Ectonucleotide pyrophosphatase/phosphodiesterase 5 (Enpp5) | 1.56 | Down | 0.002808 |
| Ring finger protein 170 | 1.56 | Down | 0.009469 |
| Serine/arginine-rich protein specific kinase 1 | 1.56 | Down | 0.001085 |
| Cylindromatosis (turban tumor syndrome) (Cyld), transcript variant 1 | 1.56 | Down | 0.010415 |
| ncrna:snoRNA chromosome:NCBIM37:9:40612831:40612920:1 gene:ENSMUSG00000064791 | 1.56 | Down | 0.030894 |
| Ribosomal protein S4, X-linked | 1.56 | Down | 0.016654 |
| Lon peptidase 2, peroxisomal (Lonp2) | 1.56 | Down | 0.003101 |
| Cyclin ania-6a (Ccn1) | 1.56 | Down | 0.02102 |
| Host cell factor C2 (Hcfc2) | 1.56 | Down | 0.010346 |
| UTP11-like, U3 small nucleolar ribonucleoprotein, (yeast) (Utp11l) | 1.56 | Down | 0.002928 |
| Phosphatidylethanolamine-binding protein (Pebp) | 1.56 | Up | 0.003268 |
| Ribosomal protein L13 (Rpl13) | 1.56 | Up | 0.020999 |
| Zinc finger protein 87 | 1.56 | Down | 0.038409 |
| Phosphatidylinositol glycan anchor biosynthesis, class F | 1.56 | Up | 0.010703 |
| ATP-binding cassette, sub-family D (ALD), member 3 | 1.56 | Down | 0.009713 |
| ncrna:snRNA chromosome:NCBIM37:9:65049533:65049648:-1 gene:ENSMUSG00000075930 | 1.56 | Up | 0.026691 |
| Tripartite motif protein 8 (Trim8) | 1.56 | Up | 0.004695 |
| CCR4-NOT transcription complex, subunit 1 (Cnot1), transcript variant 1 | 1.56 | Down | 0.017595 |
| Angiopoietin-like 4 | 1.56 | Up | 0.025977 |
| Ecotropic viral integration site 5 (Evi5) | 1.56 | Down | 0.006706 |
| Jumonji, AT rich interactive domain 1B (Rbp2 like) (Jarid1b) | 1.56 | Down | 0.011092 |
| NEDD4 binding protein 2-like 2 (N4bp2l2) | 1.56 | Down | 0.009766 |
| IKAROS family zinc finger 5 | 1.56 | Down | 0.003771 |
| Yamaguchi sarcoma viral (v-yes) oncogene homolog 1 | 1.56 | Down | 0.004523 |
| ADP-ribosylation factor-like 5A | 1.56 | Down | 0.004209 |
| 10582882 | 1.56 | Up | 0.032617 |
| Ring finger protein 5 | 1.56 | Up | 0.008618 |
| MKIAA3006 protein | 1.56 | Down | 0.024048 |
| ATP synthase, H+ transporting, mitochondrial F0 complex, subunit g | 1.56 | Up | 0.003482 |
| Adaptor-related protein complex 3, beta 1 subunit | 1.56 | Down | 0.005625 |
| Macrophage scavenger receptor 1 (Msr1), transcript variant 1 | 1.56 | Down | 0.001744 |
| CDNA clone IMAGE:40051010 | 1.56 | Down | 0.004719 |
| KRR1, small subunit (SSU) processome component, homolog (yeast) | 1.56 | Down | 0.002314 |
| Nuclear transcription factor, X-box binding 1 (Nfx1) | 1.56 | Down | 0.002725 |
| Mitochondrial ribosome recycling factor (Mrrf), nuclear gene encoding mitochondrial protein | 1.55 | Down | 0.000592 |
| Ring finger protein 213 gene:ENSMUSG00000070327 | 1.55 | Down | 0.005861 |
| Protein tyrosine phosphatase, non-receptor type 2 | 1.55 | Down | 0.00393 |
| RIKEN cDNA 4932438A13 gene | 1.55 | Down | 0.004501 |
| Predicted gene, EG639396 | 1.55 | Down | 0.005397 |
| DEAH (Asp-Glu-Ala-His) box polypeptide 15 (Dhx15), transcript variant 2 | 1.55 | Down | 0.005059 |
| Protein phosphatase 1, regulatory (inhibitor) subunit 7 | 1.55 | Down | 0.003552 |
| A disintegrin and metallopeptidase domain 10 (Adam10) | 1.55 | Down | 0.009314 |
| ATPase, Ca++ transporting, plasma membrane 1 | 1.55 | Down | 0.005705 |
| nuclear receptor subfamily 5, group A, member 2 | 1.55 | Down | 0.001631 |
| Soc-2 (suppressor of clear) homolog (C. elegans) | 1.55 | Down | 0.003699 |
| Ras association (RalGDS/AF-6) domain family member 4 | 1.55 | Up | 0.008595 |
| cdna:pseudogene chromosome:NCBIM37:14:12453002:12453359:1 gene:ENSMUSG00000072711 | 1.55 | Down | 0.006748 |
| Dynein cytoplasmic 1 intermediate chain 2 (Dync1i2) | 1.55 | Down | 0.009028 |
| cdna:Genscan chromosome:NCBIM37:7:46711759:46754888:1 | 1.55 | Down | 0.008019 |
| RIKEN cDNA 4933409K07 gene | 1.55 | Down | 0.003863 |
| RIKEN cDNA 4932438A13 gene | 1.55 | Down | 0.00609 |
| Glutamine and serine rich 1 | 1.55 | Down | 0.006393 |
| Sulfatase modifying factor 1 (Sumf1) | 1.55 | Up | 0.001584 |
| myxovirus (influenza virus) resistance 2 | 1.55 | Down | 0.046593 |
| cdna:Genscan chromosome:NCBIM37:8:107569088:107569324:1 | 1.55 | Up | 0.014209 |
| Bromodomain and WD repeat domain containing 2 | 1.55 | Down | 0.002103 |
| MKIAA0305 protein | 1.55 | Down | 0.010364 |
| RIKEN cDNA 1110057K04 gene | 1.55 | Down | 0.002046 |
| Testis expressed gene 261 | 1.55 | Up | 0.003088 |
| Protein tyrosine phosphatase, receptor type, J (Ptprj), transcript variant 1 | 1.55 | Down | 0.003087 |
| Expressed sequence AW209491 | 1.55 | Down | 0.002724 |
| Ankyrin repeat domain 17 | 1.55 | Down | 0.004695 |
| La ribonucleoprotein domain family, member 4 (Larp4), transcript variant 2 | 1.55 | Down | 0.023826 |
| Zinc finger and BTB domain containing 7a (Zbtb7a) | 1.55 | Up | 0.004388 |
| Transmembrane protein 93 | 1.55 | Up | 0.003276 |
| RIKEN cDNA 9230105E10 gene | 1.55 | Down | 0.01063 |
| RIKEN cDNA 4632427E13 gene | 1.55 | Down | 0.01274 |
| TISP73 | 1.55 | Up | 0.002653 |
| Membrane metallo endopeptidase | 1.55 | Down | 0.026159 |
| RIKEN cDNA 2010109K11 gene | 1.55 | Down | 0.003462 |
| Nucleoporin 54 | 1.55 | Down | 0.002923 |
| similar to transcription elongation factor B (SIII), polypeptide 2 | 1.55 | Up | 0.007491 |
| predicted gene 10796 | 1.55 | Down | 0.008039 |
| Protein inhibitor of activated STAT 1 (Pias1) | 1.55 | Down | 0.011006 |
| FYVE, RhoGEF and PH domain containing 6 | 1.55 | Down | 0.002724 |
| Vacuolar protein sorting 54 (yeast) | 1.54 | Down | 0.000782 |
| Protein tyrosine phosphatase, non-receptor type 3 (Ptpn3) | 1.54 | Down | 0.002928 |
| PCTAIRE-motif protein kinase 3 (Pctk3) | 1.54 | Up | 0.001136 |
| Peroxisomal biogenesis factor 1 (Pex1) | 1.54 | Down | 0.009469 |
| Armadillo repeat containing 1 (Armc1) | 1.54 | Down | 0.003089 |
| PREDICTED: Mus musculus RIKEN cDNA 2700023E23 gene | 1.54 | Up | 0.023544 |
| Meningioma expressed antigen 5 (hyaluronidase) (Mgea5) | 1.54 | Down | 0.005864 |
| RAB GTPase activating protein 1 (Rabgap1), transcript variant 1 | 1.54 | Down | 0.017283 |
| Endothelin receptor type B | 1.54 | Down | 0.005199 |
| Camello-like 1 | 1.54 | Up | 0.023405 |
| ERO1-like (S. cerevisiae) | 1.54 | Down | 0.006924 |
| cdna:known chromosome:NCBIM37:18:84730066:84758896:-1 gene:ENSMUSG00000044356 | 1.54 | Down | 0.010909 |
| Cell division cycle 5-like (S. pombe) (Cdc5l) | 1.54 | Down | 0.003875 |
| Cell division cycle 5-like (S. pombe) (Cdc5l) | 1.54 | Down | 0.003875 |
| Cell division cycle 5-like (S. pombe) (Cdc5l) | 1.54 | Down | 0.003875 |
| Cell division cycle 5-like (S. pombe) (Cdc5l) | 1.54 | Down | 0.003875 |
| Cell division cycle 5-like (S. pombe) (Cdc5l) | 1.54 | Down | 0.003875 |
| Cell division cycle 5-like (S. pombe) (Cdc5l) | 1.54 | Down | 0.003875 |
| Mannose receptor, C type 1 (Mrc1) | 1.54 | Down | 0.012531 |
| Fem-1 homolog c (C.elegans) | 1.54 | Down | 0.002738 |
| ROD1 regulator of differentiation 1 (S. pombe) (Rod1), transcript variant 1 | 1.54 | Down | 0.009933 |
| Acetyl-Coenzyme A acyltransferase 1B (Acaa1b) | 1.54 | Up | 0.026593 |
| Serine/threonine kinase UNC51.2 (Unc51.2) | 1.54 | Down | 0.003382 |
| Paraoxonase 3 | 1.54 | Down | 0.004446 |
| Ferric-chelate reductase 1 | 1.54 | Down | 0.002907 |
| HECT domain containing 1 (Hectd1) | 1.54 | Down | 0.006485 |
| Synaptosomal-associated protein 23 (Snap23) | 1.54 | Down | 0.003908 |
| ATPase family, AAA domain containing 2B (Atad2b) | 1.54 | Down | 0.005915 |
| Myc induced nuclear antigen | 1.54 | Down | 0.004212 |
| Annexin A3 (Anxa3) | 1.54 | Down | 0.009169 |
| Cyclin D3 | 1.54 | Up | 0.002012 |
| Ubiquitin protein ligase E3A (Ube3a), transcript variant 2 | 1.54 | Down | 0.010348 |
| PREDICTED: Mus musculus RIKEN cDNA 6820431F20 gene | 1.54 | Down | 0.00771 |
| Thymidine kinase 1 | 1.54 | Up | 0.0137 |
| Strain AKR/J sub-family C member 2 ATP-binding cassette protein (Abcc2) | 1.54 | Down | 0.006188 |
| UDP-N-acetyl-alpha-D-galactosamine:polypeptide N-acetylgalactosaminyltransferase 1 (Galnt1) | 1.54 | Down | 0.003934 |
| Transmembrane anterior posterior transformation 1 | 1.54 | Down | 0.001853 |
| Blocked early in transport 1 homolog (S. cerevisiae) | 1.54 | Up | 0.017749 |
| ncrna:snRNA chromosome:NCBIM37:12:60141322:60141512:1 gene:ENSMUSG00000065232 | 1.54 | Up | 0.003491 |
| Fragile X mental retardation, autosomal homolog 2 (Fxr2) | 1.54 | Up | 0.005842 |
| Serine/threonine kinase 3 (Ste20, yeast homolog) | 1.54 | Down | 0.007936 |
| predicted gene, EG382969 | 1.54 | Up | 0.014171 |
| Atlastin GTPase 2 (Atl2), transcript variant 1 | 1.54 | Down | 0.006546 |
| Sortilin 1 | 1.54 | Down | 0.043297 |
| Sec23-like A protein (Sec23a) | 1.54 | Down | 0.009716 |
| Sestrin 3 (Sesn3) | 1.54 | Down | 0.002902 |
| RIKEN cDNA 9030624J02 gene | 1.54 | Down | 0.002256 |
| THUMP domain containing 3 | 1.54 | Down | 0.010255 |
| Zinc finger protein 329 (Zfp329) | 1.54 | Down | 0.004779 |
| WD repeats and SOF domain containing 1 (Wdsof1) | 1.54 | Down | 0.021578 |
| RIKEN cDNA 4932441K18 gene | 1.53 | Down | 0.020872 |
| Neurobeachin like 1 | 1.53 | Down | 0.047633 |
| RIKEN cDNA 1110002B05 gene | 1.53 | Up | 0.00195 |
| Small nuclear ribonucleoprotein N | 1.53 | Up | 0.004111 |
| Signal transducer and activator of transcription 1 | 1.53 | Down | 0.011864 |
| RAS related protein 1b | 1.53 | Down | 0.005527 |
| Round spermatid basic protein 1-like (Rsbn1l) | 1.53 | Down | 0.002933 |
| PREDICTED: Mus musculus cDNA sequence BC023892, transcript variant 2 | 1.53 | Down | 0.003604 |
| Dihydropyrimidine dehydrogenase | 1.53 | Down | 0.035909 |
| RIKEN cDNA 0610010K06 gene | 1.53 | Down | 0.041219 |
| Heat shock factor 2 | 1.53 | Down | 0.015904 |
| CDNA clone IMAGE:30544637 | 1.53 | Down | 0.002749 |
| DCN1, defective in cullin neddylation 1, domain containing 1 (S. cerevisiae) | 1.53 | Down | 0.01123 |
| Ubiquitin domain containing 1 (Ubtd1) | 1.53 | Up | 0.003009 |
| Sialic acid acetylesterase | 1.53 | Down | 0.006393 |
| predicted gene, OTTMUSG00000011546 | 1.53 | Up | 0.004834 |
| Kelch-like 7 (Drosophila) (Klhl7) | 1.53 | Down | 0.007223 |
| PRP40 pre-mRNA processing factor 40 homolog A (yeast) (Prpf40a) | 1.53 | Down | 0.025009 |
| Ubiquitin protein ligase E3 component n-recognin 3 | 1.53 | Down | 0.011995 |
| Splicing factor, arginine/serine-rich 11 | 1.53 | Down | 0.002908 |
| DEAH (Asp-Glu-Ala-His) box polypeptide 40 (Dhx40) | 1.53 | Down | 0.006653 |
| RIKEN cDNA 1200016B10 gene | 1.53 | Down | 0.015931 |
| DnaJ (Hsp40) homolog, subfamily C, member 19 | 1.53 | Up | 0.001714 |
| Deoxyhypusine hydroxylase/monooxygenase (Dohh) | 1.53 | Up | 0.008063 |
| Poly (ADP-ribose) polymerase family, member 2 (Parp2) | 1.53 | Down | 0.005005 |
| SWI/SNF related, matrix associated, actin dependent regulator of chromatin, subfamily e, member 1 (Smarce1) | 1.53 | Down | 0.002577 |
| Atlastin GTPase 3 (Atl3) | 1.53 | Down | 0.006434 |
| GUF1 GTPase homolog (S. cerevisiae) | 1.53 | Down | 0.009801 |
| Nucleophosmin 1 (Npm1) | 1.53 | Down | 0.042986 |
| Solute carrier organic anion transporter family, member 1b2 (Slco1b2), transcript variant 1 | 1.53 | Down | 0.009716 |
| predicted gene, EG627543 | 1.53 | Down | 0.00819 |
| Brix domain containing 2 (Bxdc2) | 1.53 | Down | 0.026253 |
| Bromodomain containing 7 (Brd7) | 1.53 | Down | 0.003964 |
| Aspartyl-tRNA synthetase 2 (mitochondrial) | 1.53 | Down | 0.002653 |
| Angiopoietin-like 3 | 1.53 | Down | 0.018459 |
| Ubiquitin specific peptidase 48 (Usp48) | 1.53 | Down | 0.005243 |
| Kelch-like 28 (Drosophila) | 1.53 | Down | 0.003166 |
| Molybdenum cofactor synthesis 2 | 1.53 | Down | 0.004454 |
| Spermidine synthase | 1.53 | Up | 0.001877 |
| Mucosa associated lymphoid tissue lymphoma translocation gene 1 | 1.53 | Down | 0.003482 |
| Exocyst complex component 4 | 1.53 | Down | 0.011057 |
| Ubiquitin-conjugating enzyme E2E 2 (UBC4/5 homolog, yeast) (Ube2e2) | 1.53 | Up | 0.002902 |
| Cadherin 2 | 1.53 | Down | 0.002103 |
| Ring finger protein 213 | 1.53 | Down | 0.003096 |
| 10596263 | 1.53 | Down | 0.006984 |
| Trans-acting transcription factor 3 | 1.53 | Down | 0.011797 |
| RIKEN cDNA 4933425L03 gene | 1.53 | Down | 0.003265 |
| FIG4 homolog (S. cerevisiae) | 1.53 | Down | 0.002161 |
| DENN/MADD domain containing 4C | 1.53 | Down | 0.010383 |
| Asparaginyl-tRNA synthetase (Nars), transcript variant 1 | 1.53 | Down | 0.002171 |
| RIKEN cDNA 1700023B02 gene | 1.53 | Down | 0.004209 |
| similar to Glyceraldehyde-3-phosphate dehydrogenase (GAPDH) | 1.53 | Up | 0.002902 |
| ncrna:snoRNA chromosome:NCBIM37:5:130295415:130295549:1 gene:ENSMUSG00000065304 | 1.53 | Up | 0.018067 |
| ELMO domain containing 2 | 1.53 | Down | 0.003347 |
| Interferon induced with helicase C domain 1 (Ifih1) | 1.53 | Down | 0.024701 |
| S-adenosylmethionine decarboxylase 1 | 1.53 | Down | 0.034463 |
| RIKEN cDNA 1110037F02 gene | 1.53 | Down | 0.0141 |
| predicted gene 9884 | 1.52 | Down | 0.014266 |
| Zinc finger protein 397 | 1.52 | Down | 0.00851 |
| TRM5 tRNA methyltransferase 5 homolog (S. cerevisiae) (Trmt5) | 1.52 | Down | 0.007017 |
| N-TAF1 mRNA for TAF1 RNA polymerase II, TATA box binding protein (TBP)-associated factor, neuron specific isoform | 1.52 | Down | 0.00883 |
| Small nucleolar RNA, H/ACA box 69 (Snora69), non-coding RNA | 1.52 | Up | 0.007805 |
| small nucleolar RNA, C/D box 116 cluster | 1.52 | Up | 0.04321 |
| Protein phosphatase 4, regulatory subunit 1 (Ppp4r1), transcript variant 1 | 1.52 | Down | 0.000782 |
| RIKEN cDNA 2410002O22 gene | 1.52 | Down | 0.009638 |
| Cytochrome P450, family 2, subfamily c, polypeptide 40 (Cyp2c40) | 1.52 | Down | 0.019289 |
| S-adenosylmethionine decarboxylase 1 | 1.52 | Down | 0.042956 |
| Son of sevenless 2 | 1.52 | Down | 0.002107 |
| Syndecan 1 | 1.52 | Up | 0.002478 |
| SH3-binding kinase 1 (Sbk1) | 1.52 | Up | 0.011633 |
| RAB4A, member RAS oncogene family (Rab4a) | 1.52 | Up | 0.002084 |
| ncrna:snoRNA chromosome:NCBIM37:17:35299508:35299641:-1 gene:ENSMUSG00000064853 | 1.52 | Up | 0.024045 |
| TATA box binding protein (Tbp)-associated factor, RNA polymerase I, B (Taf1b) | 1.52 | Down | 0.013245 |
| Tankyrase, TRF1-interacting ankyrin-related ADP-ribose polymerase | 1.52 | Down | 0.011612 |
| PDS5, regulator of cohesion maintenance, homolog B (S. cerevisiae) | 1.52 | Down | 0.032646 |
| Pleckstrin homology-like domain, family B, member 2 (Phldb2) | 1.52 | Down | 0.005438 |
| Adenosine kinase (Adk) | 1.52 | Down | 0.005402 |
| IQ motif containing GTPase activating protein 2 | 1.52 | Down | 0.015944 |
| Pwcr1 mRNA, complete sequence | 1.52 | Up | 0.017976 |
| Erythrocyte protein band 4.1-like 5 (Epb4.1l5), transcript variant 1 | 1.52 | Down | 0.004888 |
| EF hand domain containing 2 (Efhd2) | 1.52 | Up | 0.003274 |
| Expressed sequence AI413782 | 1.52 | Down | 0.002331 |
| Expressed sequence AI848100 | 1.52 | Down | 0.010949 |
| ArfGAP with coiled-coil, ankyrin repeat and PH domains 2 (Acap2) | 1.52 | Down | 0.003361 |
| Guanine nucleotide binding protein-like 3 (nucleolar) (Gnl3), transcript variant 1 | 1.52 | Down | 0.011257 |
| Kelch domain containing 3 | 1.52 | Up | 0.005523 |
| Nuclear body associated kinase 2b (Nbak2) mRNA, alternatively spliced | 1.52 | Down | 0.001294 |
| Prostaglandin E synthase 3 (cytosolic) | 1.52 | Up | 0.004023 |
| Eukaryotic translation initiation factor 5 | 1.52 | Up | 0.022051 |
| Small nuclear ribonucleoprotein E | 1.52 | Up | 0.007141 |
| CD55 antigen | 1.52 | Down | 0.009434 |
| Tight junction protein 1 | 1.52 | Down | 0.013241 |
| Guanine nucleotide exchange factor (Larg) | 1.52 | Down | 0.003447 |
| Cell division cycle 5-like (S. pombe) (Cdc5l) | 1.52 | Down | 0.004521 |
| Cell division cycle 5-like (S. pombe) (Cdc5l) | 1.52 | Down | 0.004521 |
| STAM binding protein | 1.52 | Down | 0.004677 |
| Mitochondrial tumor suppressor 1 | 1.52 | Down | 0.003616 |
| Protein phosphatase 2, regulatory subunit B (B56), gamma isoform (Ppp2r5c), transcript variant 1 | 1.52 | Down | 0.003261 |
| Ornithine transcarbamylase | 1.52 | Down | 0.033403 |
| Sestrin 1 | 1.52 | Down | 0.015225 |
| PREDICTED: Mus musculus hect domain and RLD 5, transcript variant 1 (Herc5) | 1.52 | Down | 0.009198 |
| predicted gene, EG665806 | 1.52 | Up | 0.009104 |
| Histone cluster 1, H4f (Hist1h4f) | 1.52 | Up | 0.004384 |
| Heat shock protein 1 (chaperonin 10) | 1.52 | Up | 0.002331 |
| S-adenosylhomocysteine hydrolase-like 1 (Ahcyl1) | 1.52 | Down | 0.003555 |
| Clone MBI-3 H/ACA box snoRNA, partial sequence | 1.52 | Up | 0.026371 |
| Attractin like 1 | 1.52 | Down | 0.004024 |
| Tetratricopeptide repeat domain 39B | 1.52 | Down | 0.007338 |
| RIKEN cDNA 4932438A13 gene | 1.52 | Down | 0.032206 |
| Kinase insert domain protein receptor | 1.52 | Down | 0.01164 |
| Ubiquitin specific peptidase 8 | 1.52 | Down | 0.004045 |
| Eukaryotic translation initiation factor 2B, subunit 1 (alpha) (Eif2b1) | 1.52 | Down | 0.002577 |
| THO complex 2 (Thoc2) | 1.52 | Down | 0.013018 |
| Latrophilin 2 | 1.52 | Down | 0.004805 |
| Adaptor-related protein complex 3, mu 1 subunit | 1.52 | Down | 0.035186 |
| Mitochondrial ribosomal protein S36 | 1.52 | Up | 0.008224 |
| ncrna:misc_RNA chromosome:NCBIM37:18:36961521:36961656:1 gene:ENSMUSG00000065145 | 1.52 | Up | 0.010596 |
| CD-1 orphan receptor TAK1 (TAK1) | 1.52 | Down | 0.003908 |
| Tet oncogene family member 2 | 1.52 | Down | 0.01936 |
| CWC22 spliceosome-associated protein homolog (S. cerevisiae) | 1.52 | Down | 0.039842 |
| CWC22 spliceosome-associated protein homolog (S. cerevisiae) | 1.52 | Down | 0.039842 |
| CWC22 spliceosome-associated protein homolog (S. cerevisiae) | 1.52 | Down | 0.039842 |
| CWC22 spliceosome-associated protein homolog (S. cerevisiae) | 1.52 | Down | 0.039842 |
| CWC22 spliceosome-associated protein homolog (S. cerevisiae) | 1.52 | Down | 0.039842 |
| CWC22 spliceosome-associated protein homolog (S. cerevisiae) | 1.52 | Down | 0.039842 |
| CWC22 spliceosome-associated protein homolog (S. cerevisiae) | 1.52 | Down | 0.039842 |
| nucleoporin 205 | 1.52 | Down | 0.007514 |
| RIKEN cDNA 0610010F05 gene | 1.51 | Down | 0.005588 |
| cdna:pseudogene chromosome:NCBIM37:2:35346733:35347039:-1 gene:ENSMUSG00000081421 | 1.51 | Up | 0.002724 |
| Zinc finger protein 110 | 1.51 | Down | 0.009933 |
| RIKEN cDNA 2010109K11 gene | 1.51 | Up | 0.002012 |
| Fragilis R | 1.51 | Up | 0.007945 |
| Mutated in colorectal cancers (Mcc), transcript variant 1 | 1.51 | Down | 0.01149 |
| ncrna:snRNA chromosome:NCBIM37:9:90246833:90246977:1 gene:ENSMUSG00000065881 | 1.51 | Up | 0.031193 |
| U73B small nuclear RNA | 1.51 | Up | 0.010466 |
| Signal recognition particle 14 | 1.51 | Up | 0.006314 |
| Lysophosphatidylglycerol acyltransferase 1 | 1.51 | Down | 0.008177 |
| Non-POU-domain-containing, octamer binding protein (Nono) | 1.51 | Down | 0.003625 |
| CLIP associating protein 2 | 1.51 | Down | 0.005728 |
| Achalasia, adrenocortical insufficiency, alacrimia (Aaas) | 1.51 | Up | 0.005571 |
| Hbs1-like (S. cerevisiae) | 1.51 | Down | 0.002418 |
| Calcium/calmodulin-dependent protein kinase II inhibitor 1 (Camk2n1) | 1.51 | Up | 0.001838 |
| Catenin (cadherin associated protein), alpha 1 (Ctnna1) | 1.51 | Down | 0.006563 |
| Histone cluster 2, H2bb (Hist2h2bb) | 1.51 | Up | 0.009051 |
| Vinculin | 1.51 | Down | 0.001921 |
| Family with sequence similarity 102, member A (Fam102a) | 1.51 | Up | 0.012935 |
| Alanine-glyoxylate aminotransferase 2 | 1.51 | Down | 0.009293 |
| CDNA clone IMAGE:30021652 | 1.51 | Down | 0.021257 |
| DnaJ (Hsp40) homolog, subfamily C, member 7 | 1.51 | Down | 0.003391 |
| Strain C57BL/6J nuclear factor I/B (Nfib) | 1.51 | Down | 0.009338 |
| Pantothenate kinase 3 (Pank3) | 1.51 | Down | 0.042284 |
| Seryl-aminoacyl-tRNA synthetase 2 (Sars2) | 1.51 | Up | 0.002961 |
| EIA (Serpinb1) | 1.51 | Down | 0.041272 |
| F-box protein 30 | 1.51 | Down | 0.011121 |
| Insulin-like growth factor binding protein, acid labile subunit (Igfals) | 1.51 | Up | 0.036668 |
| Cytochrome P450, family 39, subfamily a, polypeptide 1 | 1.51 | Down | 0.00993 |
| Inhibitor of DNA binding 1 | 1.51 | Up | 0.02639 |
| Gene model 561, (NCBI) | 1.51 | Up | 0.005005 |
| PREDICTED: Mus musculus cDNA sequence BC043476, transcript variant 2 | 1.51 | Down | 0.025397 |
| TISP38 | 1.51 | Down | 0.010755 |
| TATA element modulatory factor 1 | 1.51 | Down | 0.010244 |
| CDNA sequence BC057079 | 1.51 | Down | 0.007536 |
| Hyaluronan and proteoglycan link protein 4 | 1.51 | Up | 0.02053 |
| Eukaryotic translation initiation factor 1B | 1.51 | Up | 0.005914 |
| predicted gene, OTTMUSG00000025797 | 1.51 | Up | 0.015017 |
| Pelota homolog (Drosophila) (Pelo) | 1.51 | Down | 0.011289 |
| SFFV proviral integration 1 | 1.51 | Up | 0.006796 |
| Metadherin (Mtdh) | 1.51 | Down | 0.00577 |
| Kininogen 2 (Kng2), transcript variant 3 | 1.51 | Down | 0.0037 |
| RIKEN cDNA B130055D15 gene | 1.51 | Down | 0.006023 |
| Recombining binding protein suppressor of hairless (Drosophila) | 1.51 | Down | 0.005692 |
| Thymoma viral proto-oncogene 1 (Akt1) | 1.51 | Up | 0.003526 |
| Orphan receptor (TR2) | 1.51 | Down | 0.014156 |
| Acyl-Coenzyme A dehydrogenase, medium chain | 1.51 | Down | 0.007172 |
| Transmembrane protein 189 (Tmem189) | 1.51 | Up | 0.016317 |
| Protein tyrosine phosphatase, receptor type, J (Ptprj), transcript variant 1 | 1.51 | Down | 0.001921 |
| RIKEN cDNA 4732479N06 gene | 1.51 | Down | 0.003688 |
| TAF4B RNA polymerase II, TATA box binding protein (TBP)-associated factor | 1.51 | Down | 0.002037 |
| Cytoplasmic tyrosine kinase, Dscr28C related (Drosophila) | 1.51 | Down | 0.014753 |
| Solute carrier family 37 (glucose-6-phosphate transporter), member 4 (Slc37a4) | 1.51 | Up | 0.002908 |
| Ribosomal protein L4 | 1.51 | Down | 0.002012 |
| Resistance to inhibitors of cholinesterase 8 homolog B (C. elegans) (Ric8b), transcript variant 1 | 1.51 | Down | 0.002321 |
| NADH dehydrogenase (ubiquinone) Fe-S protein 1 (Ndufs1), nuclear gene encoding mitochondrial protein | 1.51 | Down | 0.009197 |
| 8-oxoguanine DNA-glycosylase 1 (Ogg1), nuclear gene encoding mitochondrial protein | 1.51 | Down | 0.001346 |
| Beta-amyloid binding protein (Bbp) | 1.51 | Up | 0.010778 |
| Period homolog 2 (Drosophila) (Per2) | 1.51 | Up | 0.011169 |
| Eukaryotic translation initiation factor 2, subunit 3, structural gene X-linked | 1.51 | Down | 0.004402 |
| Transmembrane 4 superfamily member 1 (Tm4sf1) | 1.51 | Down | 0.011049 |
| Rho family GTPase 3 | 1.51 | Down | 0.005726 |
| cdna:known chromosome:NCBIM37:X:151585495:151585797:-1 gene:ENSMUSG00000045694 | 1.51 | Up | 0.013203 |
| interferon regulatory factor 9 | 1.51 | Down | 0.002902 |
| CD52 antigen | 1.51 | Up | 0.017094 |
| Guanine nucleotide binding protein (G protein), gamma 5 | 1.51 | Up | 0.004111 |
| RIKEN cDNA 1810022C23 gene | 1.51 | Down | 0.009513 |
| RIKEN cDNA 1110005A23 gene | 1.51 | Down | 0.024679 |
| Oxidation resistance 1 (Oxr1), transcript variant 1 | 1.51 | Down | 0.006535 |
| Chromobox homolog 5 (Drosophila HP1a) | 1.51 | Down | 0.004548 |
| Acyl-CoA thioesterase 1 | 1.51 | Up | 0.002037 |
| Solute carrier family 36 (proton/amino acid symporter), member 4 | 1.51 | Down | 0.010237 |
| AT rich interactive domain 4B (RBP1-like | 1.51 | Down | 0.005254 |
| Dehydrogenase/reductase (SDR family) member 9 | 1.51 | Down | 0.006374 |
| RIKEN cDNA 3110043O21 gene | 1.51 | Down | 0.013441 |
| Forkhead box N3 | 1.5 | Down | 0.015128 |
| RIKEN cDNA 4932438A13 gene | 1.5 | Down | 0.022835 |
| X-prolyl aminopeptidase (aminopeptidase P) 3, putative | 1.5 | Down | 0.002808 |
| Tripartite motif protein TRIM23 | 1.5 | Down | 0.025508 |
| Programmed cell death 4 (Pdcd4) | 1.5 | Down | 0.025779 |
| similar to syndecan-1 | 1.5 | Up | 0.003315 |
| TAO kinase 1 | 1.5 | Down | 0.019074 |
| Transmembrane protein 98 (Tmem98) | 1.5 | Up | 0.046577 |
| Plasma glutamate carboxypeptidase | 1.5 | Down | 0.007029 |
| WD repeat domain 75 | 1.5 | Down | 0.004745 |
| MAP Kinase Kinase | 1.5 | Up | 0.021939 |
| Transcription factor 4 | 1.5 | Down | 0.002902 |
| Poly (ADP-ribose) glycohydrolase (Parg) | 1.5 | Down | 0.008471 |
| Heat shock protein 8 | 1.5 | Up | 0.002037 |
| RIKEN cDNA 2610301F02 gene | 1.5 | Down | 0.027689 |
| Acid ceramidase (Asah1) | 1.5 | Down | 0.012521 |
| Sepiapterin reductase | 1.5 | Up | 0.002103 |
| RIKEN cDNA 5730455P16 gene | 1.5 | Down | 0.003875 |
| Folliculin interacting protein 2 | 1.5 | Down | 0.017696 |
| B6D2F1 clone 2C11B mRNA | 1.5 | Down | 0.005003 |
| Ring finger protein 144B (Rnf144b) | 1.5 | Down | 0.002012 |
| Cyclin-dependent kinase 2 | 1.5 | Up | 0.006845 |
| Protein phosphatase 1, regulatory (inhibitor) subunit 12A | 1.5 | Down | 0.004062 |
| Adenylosuccinate synthetase like 1 (Adssl1) | 1.5 | Up | 0.000805 |
| apolipoprotein O, pseudogene | 1.5 | Up | 0.006617 |
| Platelet-derived growth factor, D polypeptide | 1.5 | Down | 0.012309 |
| Ribosomal protein S6 kinase, polypeptide 1 | 1.5 | Down | 0.001877 |
| Receptor (TNFRSF)-interacting serine-threonine kinase 1 (Ripk1) | 1.5 | Down | 0.004272 |
| predicted gene, EG633736 | 1.5 | Up | 0.003964 |
| Nucleoporin like 1 (Nupl1) | 1.5 | Down | 0.003695 |
| heat shock protein 8 | 1.5 | Down | 0.04861 |
| Interleukin 6 signal transducer (Il6st) | 1.5 | Down | 0.003964 |
| RIKEN cDNA 5730403M16 gene | 1.5 | Down | 0.007693 |
| G1 to S phase transition 1 (Gspt1), transcript variant 1 | 1.5 | Down | 0.003537 |
| Tumor suppressor candidate 3 | 1.5 | Up | 0.001792 |
| Isoleucine-tRNA synthetase | 1.5 | Down | 0.005252 |
| Ephrin A3 (Efna3) | 1.5 | Down | 0.010637 |
| MutL homolog 3 (E coli) ( | 1.5 | Down | 0.009505 |
| Acyl-Coenzyme A dehydrogenase, short/branched chain | 1.5 | Down | 0.022558 |
| Rho guanine nucleotide exchange factor (GEF) 5 | 1.5 | Down | 0.001856 |
| Transmembrane protein 41a (Tmem41a) | 1.5 | Up | 0.012235 |
| MRNA sequence | 1.5 | Down | 0.012053 |
| CDNA clone IMAGE:4016974 | 1.5 | Down | 0.030931 |
| THO complex 1 (Thoc1) | 1.5 | Down | 0.031008 |
| Anterior pharynx defective 1a homolog (C. elegans) (Aph1a), transcript variant 1 | 1.5 | Up | 0.002305 |
| Solute carrier family 2 (facilitated glucose transporter), member 2 | 1.5 | Down | 0.012911 |
| solute carrier family 39 (zinc transporter), member 1 | 1.5 | Up | 0.007831 |
| Cell division cycle 34 homolog (S. cerevisiae) | 1.5 | Up | 0.014677 |
| ncrna:miRNA chromosome:NCBIM37:16:73974686:73974783:1 gene:ENSMUSG00000076284 | 1.5 | Up | 0.006661 |
| RIKEN cDNA 4732429D16 gene | 1.5 | Down | 0.003228 |
| Kelch repeat and BTB (POZ) domain containing 4 | 1.5 | Down | 0.003228 |
| Zinc finger protein 119 (Zfp119) | 1.5 | Down | 0.009028 |
| Amino carboxymuconate semialdehyde decarboxylase | 1.5 | Down | 0.04087 |
| Mbt domain containing 1 (Mbtd1) | 1.5 | Down | 0.009536 |
| Transducin (beta)-like 1X-linked receptor 1 | 1.5 | Down | 0.011065 |
| Neuronal PAS domain protein 2 (Npas2) | 1.5 | Down | 0.010388 |
| PREDICTED: Mus musculus protein prenyltransferase alpha subunit repeat containing 1, transcript variant 1 (Ptar1) | 1.5 | Down | 0.001631 |
| zinc finger CCCH type, antiviral 1 | 1.5 | Down | 0.001611 |
| Transmembrane protein 86A (Tmem86a) | 1.5 | Up | 0.010384 |
| GRAM domain containing 1C (Gramd1c) | 1.5 | Down | 0.005438 |
| T-cell specific GTPase | 1.5 | Down | 0.022068 |
| Kinesin-like protein KIF1B (Kif1b) | 1.5 | Down | 0.003604 |

*In some instances, where a gene name is not available, a gene ID has been provided instead
