# Supplementary material for: 8-Oxoguanine DNA Glycosylase (OGG1) Deficiency Increases Susceptibility to Obesity and Metabolic Dysfunction
Source: PLoS One. 2012 Dec 17;7(12):e51697. doi: 10.1371/journal.pone.0051697 (PMC3524114; doi:10.1371/journal.pone.0051697)
Supplement: Table S3 — DEPs identified by iReport in chow-fed Ogg1−/− livers. Pairwise analysis of chow-fed WT vs. Ogg1−/− was conducted by iReport (Ingenuity Systems, Redwood City, CA), with WT mice were designated as the control group, and Ogg1−/− mice designated as the experimental group. Probesets that were differentially expressed by at least 1.5 fold and with an adjusted p<0.05 are presented. n = 6 in each group. (DOC) [file pone.0051697.s004.doc]

**Supporting Table S3: DEPs in chow-fed *Ogg1^-/-^*livers, compared to chow-fed WT livers, from iReport analysis**

| **Gene Symbol** | **Fold Change** | **P-Value** |
| --- | --- | --- |
| **Usp2** | 5.102365 | 3.20393E-06 |
| **Bhlhe41** | 4.312267 | 3.20393E-06 |
| **Gm129** | 4.046061 | 0.000814563 |
| **Rgs16** | 3.472978 | 0.035639942 |
| **Dbp** | 2.778841 | 0.000881202 |
| **Tmeff2** | 2.631569 | 6.07757E-05 |
| **Wee1** | 2.619596 | 6.03275E-05 |
| **Ugt1a7c** | 2.218517 | 0.028844094 |
| **Per1** | 2.009449 | 0.022148903 |
| **Per3** | 1.961375 | 0.000918609 |
| **Gabrb3** | 1.828627 | 0.010920984 |
| **Tef** | 1.789967 | 0.00328732 |
| **Coq10b** | 1.763748 | 0.049108745 |
| **Nrg4** | 1.737532 | 0.029987339 |
| **Klf13** | 1.644481 | 0.009486482 |
| **N4bp2l1** | 1.602005 | 0.005946374 |
| **1600002H07Rik** | 1.587143 | 0.03691056 |
| **Sumf1** | 1.58067 | 0.000259796 |
| **Plekhf1** | 1.543562 | 0.027111724 |
| **Stbd1** | 1.528851 | 0.035088938 |
| **Mtss1** | 1.509797 | 0.00064285 |
| **Adrb3** | -1.52636 | 0.044582085 |
| **Apobec1** | -1.59897 | 0.038268093 |
| **Cyp8b1** | -1.61308 | 0.036520157 |
| **St5** | -1.61589 | 0.010920984 |
| **Csrp3** | -1.65389 | 0.0043035 |
| **Papss1** | -1.67793 | 0.000814563 |
| **Cenpm** | -1.6819 | 0.047536049 |
| **Trim13** | -1.69947 | 0.036520157 |
| **Bid** | -1.71443 | 0.000270754 |
| **G6pc** | -1.78319 | 0.005946374 |
| **Camk1d** | -1.78623 | 0.044582085 |
| **Rnf125** | -1.8097 | 0.023135232 |
| **Rdh11** | -1.8498 | 0.029987339 |
| **Clpx** | -1.98136 | 0.042931044 |
| **Chka** | -2.00384 | 0.004753626 |
| **Gm11437** | -2.34623 | 0.035088938 |
| **Aqp8** | -2.49254 | 0.010920984 |
| **Gm10319** | -2.59182 | 0.01708506 |
| **Mug-ps1** | -2.65517 | 0.028955923 |
| **Nipal1** | -2.66148 | 0.042931044 |
| **Arntl** | -2.70849 | 0.000814563 |
| **Npas2** | -2.78701 | 0.030556972 |
| **Mug2** | -7.63087 | 3.20393E-06 |
