# Supplementary material for: 8-Oxoguanine DNA Glycosylase (OGG1) Deficiency Increases Susceptibility to Obesity and Metabolic Dysfunction
Source: PLoS One. 2012 Dec 17;7(12):e51697. doi: 10.1371/journal.pone.0051697 (PMC3524114; doi:10.1371/journal.pone.0051697)
Supplement: Table S4 — DEPs identified by iReport in HFD-fed Ogg1−/− livers. Pairwise analysis of HFD-fed WT vs. Ogg1−/was conducted by iReport (Ingenuity Systems, Redwood City, CA), with WT mice were designated as the control group, and Ogg1−/− mice designated as the experimental group. Probesets that were differentially expressed by at least 1.5 fold and with an adjusted p<0.05 are presented. n = 6 in each group. (DOC) [file pone.0051697.s005.doc]

**Supporting Table S4**: **DEPs in HFD-fed *Ogg1^-/-^*livers, compared to HFD-fed WT livers, from iReport analysis**

| **Gene Symbol** | **Fold Change** | **P-Value** |
| --- | --- | --- |
| **Cyp2b13** | 9.559698 | 0.001354 |
| **Usp2** | 3.915984 | 0.000298 |
| **Gm129** | 3.699006 | 0.000234 |
| **Gm5331** | 2.893283 | 0.000977 |
| **Per1** | 2.788791 | 0.000393 |
| **Acot3** | 2.608781 | 0.000755 |
| **Zbtb16** | 2.555443 | 0.00317 |
| **Hamp2** | 2.553132 | 0.000213 |
| **Tsku** | 2.476931 | 0.004251 |
| **Ccdc72** | 2.440129 | 0.002333 |
| **Aph1a** | 2.400295 | 0.004524 |
| **LOC100502908** | 2.231727 | 0.00029 |
| **Por** | 2.227947 | 6.3E-06 |
| **Snord68** | 2.185344 | 0.006209 |
| **H2afz** | 2.152567 | 0.000767 |
| **Dbp** | 2.108134 | 0.000171 |
| **Igh-2** | 2.105581 | 0.00156 |
| **Gm5643** | 2.072237 | 0.003821 |
| **Rgs16** | 2.065164 | 0.041112 |
| **Mmd2** | 2.058707 | 0.019133 |
| **Tspan4** | 2.053397 | 2.7E-05 |
| **Tomm22** | 2.042768 | 0.010695 |
| **Gm6484** | 1.954067 | 0.001852 |
| **Wee1** | 1.952642 | 0.000214 |
| **Spry4** | 1.951384 | 0.003457 |
| **Marveld1** | 1.948015 | 0.000225 |
| **Srebf1** | 1.942698 | 0.000295 |
| **B4galt5** | 1.910545 | 0.000444 |
| **Sds** | 1.891117 | 0.001251 |
| **Acnat2** | 1.886996 | 0.010733 |
| **Nat8** | 1.877121 | 0.000267 |
| **LOC100504651** | 1.875432 | 0.002133 |
| **Bhlhe41** | 1.863937 | 0.007043 |
| **Tef** | 1.861447 | 0.000171 |
| **LOC100503724** | 1.854972 | 0.000177 |
| **D0H4S114** | 1.850978 | 0.034655 |
| **Klf13** | 1.832416 | 0.000178 |
| **4833442J19Rik** | 1.827954 | 0.001217 |
| **BC048355** | 1.825907 | 0.000244 |
| **Acot4** | 1.808597 | 0.002479 |
| **Ccrn4l** | 1.799672 | 0.023857 |
| **N4bp2l1** | 1.797404 | 0.000554 |
| **Kcnk5** | 1.783462 | 0.038369 |
| **Mid1ip1** | 1.776908 | 0.000687 |
| **Mfsd2a** | 1.769843 | 0.02766 |
| **Coq10b** | 1.760755 | 0.001414 |
| **Tmem69** | 1.759797 | 0.000592 |
| **Wfdc2** | 1.758372 | 0.047669 |
| **Gm5093** | 1.752665 | 0.000662 |
| **Cdk10** | 1.752175 | 0.000533 |
| **Snord82** | 1.750985 | 0.002748 |
| **Cr1l** | 1.749605 | 0.0059 |
| **Nudt19** | 1.737467 | 0.000347 |
| **G0s2** | 1.737365 | 0.016735 |
| **S1pr5** | 1.737146 | 0.002184 |
| **Nnmt** | 1.730115 | 0.001079 |
| **Gm5786** | 1.727158 | 0.001506 |
| **Gpt** | 1.719961 | 8.61E-05 |
| **Nr0b2** | 1.717445 | 0.001979 |
| **Cks1b** | 1.713626 | 0.000291 |
| **Rassf4** | 1.712761 | 0.000849 |
| **Cyp2b10** | 1.712717 | 0.018195 |
| **Mknk2** | 1.710508 | 0.000308 |
| **Bag3** | 1.708016 | 0.001225 |
| **Sumo1** | 1.705646 | 0.003151 |
| **Tmem60** | 1.70435 | 0.000275 |
| **Tk1** | 1.703494 | 0.001722 |
| **Chchd2** | 1.700586 | 0.001156 |
| **Tmed2** | 1.698346 | 0.038416 |
| **Car14** | 1.698005 | 0.000343 |
| **Hamp** | 1.692431 | 0.019238 |
| **Nrg4** | 1.691489 | 0.003278 |
| **Notum** | 1.690268 | 0.004249 |
| **Gm10651** | 1.688006 | 0.001722 |
| **Etnk2** | 1.686981 | 0.001023 |
| **LOC100504611** | 1.686722 | 0.000384 |
| **1810027O10Rik** | 1.681208 | 0.000429 |
| **Hist1h4f** | 1.678881 | 0.004754 |
| **Dnajb9** | 1.676472 | 0.000267 |
| **Atxn7l3** | 1.675541 | 0.000295 |
| **2410002F23Rik** | 1.670079 | 0.010985 |
| **LOC100048057** | 1.663544 | 0.047273 |
| **Rprl1** | 1.661442 | 0.000839 |
| **Angptl4** | 1.659902 | 0.003182 |
| **Ptpla** | 1.659198 | 0.000377 |
| **Ifitm2** | 1.658569 | 0.000858 |
| **Eid1** | 1.655987 | 0.000456 |
| **Gm8909** | 1.655153 | 0.002272 |
| **Nudt21** | 1.65148 | 0.049935 |
| **Cml1** | 1.648475 | 0.0059 |
| **Dhrs11** | 1.643158 | 0.000463 |
| **Acy1** | 1.64286 | 0.000306 |
| **Gm6155** | 1.64218 | 0.000909 |
| **2900010J23Rik** | 1.642135 | 0.00028 |
| **Acaa1b** | 1.639781 | 0.003025 |
| **1600002H07Rik** | 1.6391 | 0.012404 |
| **Nnt** | 1.637493 | 0.000177 |
| **Aldh1b1** | 1.637236 | 0.003376 |
| **Fam195a** | 1.633265 | 0.000178 |
| **Cdk18** | 1.631837 | 0.000172 |
| **Alas1** | 1.627018 | 0.006876 |
| **Snora21** | 1.618427 | 0.003556 |
| **H2-DMb1** | 1.616177 | 0.009776 |
| **Hist2h2bb** | 1.615999 | 0.005541 |
| **Hist1h2ab** | 1.614668 | 0.000753 |
| **Gm4997** | 1.613587 | 0.002276 |
| **Chchd1** | 1.613257 | 0.000528 |
| **Cebpb** | 1.612865 | 0.00072 |
| **Rheb** | 1.612712 | 0.000343 |
| **Tmem14a** | 1.610728 | 0.000296 |
| **Sema5b** | 1.610584 | 0.004915 |
| **Praf2** | 1.610056 | 0.000496 |
| **C030006K11Rik** | 1.60969 | 0.000229 |
| **Gng5** | 1.609089 | 0.000892 |
| **Macrod1** | 1.608496 | 0.000178 |
| **Trim8** | 1.607344 | 0.000525 |
| **Mbl2** | 1.601672 | 0.00017 |
| **Sumf1** | 1.597786 | 0.000152 |
| **Igkv3-4** | 1.59752 | 0.045745 |
| **Fxr2** | 1.597515 | 0.00079 |
| **Snord34** | 1.597474 | 0.008561 |
| **Crls1** | 1.597076 | 0.000234 |
| **Rnf5** | 1.596302 | 0.000988 |
| **Sbk1** | 1.596286 | 0.001988 |
| **Snora69** | 1.594894 | 0.00069 |
| **Stk35** | 1.589559 | 0.000283 |
| **Tex261** | 1.588252 | 0.000391 |
| **Tusc3** | 1.588078 | 0.000178 |
| **Dnajb1** | 1.582862 | 0.004145 |
| **Srp14** | 1.582009 | 0.000542 |
| **1110058L19Rik** | 1.581702 | 0.000783 |
| **Hapln4** | 1.581475 | 0.002691 |
| **Rps27a** | 1.580474 | 0.001407 |
| **D1Ertd622e** | 1.579227 | 0.000355 |
| **Bet1** | 1.57885 | 0.002175 |
| **Gm4889** | 1.578319 | 0.00212 |
| **Rab4a** | 1.577579 | 0.000192 |
| **Fam102a** | 1.577297 | 0.00115 |
| **Id1** | 1.57713 | 0.003746 |
| **Ndn** | 1.576872 | 0.000268 |
| **Rhob** | 1.57599 | 0.00066 |
| **Pcp4l1** | 1.573775 | 0.022267 |
| **Ubtd1** | 1.573627 | 0.000523 |
| **Gm6252** | 1.573197 | 0.020485 |
| **Arsa** | 1.57168 | 0.000283 |
| **Pebp1** | 1.571216 | 0.000744 |
| **Gm3550** | 1.568314 | 0.000802 |
| **Gm11942** | 1.566764 | 0.001923 |
| **Rnu73b** | 1.565707 | 0.001478 |
| **Klhdc3** | 1.565694 | 0.000644 |
| **Gpr182** | 1.565406 | 0.000434 |
| **Egln1** | 1.564802 | 0.000295 |
| **Efhd2** | 1.563986 | 0.00046 |
| **2700023E23Rik** | 1.563299 | 0.002052 |
| **Sdc1** | 1.560336 | 0.000218 |
| **Fam108a** | 1.560204 | 0.000292 |
| **Vaultrc5** | 1.559372 | 0.00138 |
| **Slc37a4** | 1.558643 | 0.000299 |
| **Tob2** | 1.558299 | 0.001284 |
| **Camk2n1** | 1.558033 | 0.000177 |
| **Ccnd3** | 1.557869 | 0.000372 |
| **Ube2a** | 1.556939 | 0.000378 |
| **Dohh** | 1.555128 | 0.001182 |
| **Igfals** | 1.554933 | 0.007127 |
| **Xrcc6bp1** | 1.554818 | 0.000266 |
| **Fuz** | 1.554041 | 0.000292 |
| **Snora28** | 1.553785 | 0.004094 |
| **Tmem98** | 1.552462 | 0.010066 |
| **Zbtb7a** | 1.548825 | 0.00077 |
| **Ube2e2** | 1.548427 | 0.00038 |
| **Gtf2a2** | 1.54731 | 0.003806 |
| **Pigf** | 1.547212 | 0.001503 |
| **Hist1h2bj** | 1.546847 | 0.005792 |
| **Cd52** | 1.546303 | 0.003537 |
| **Tmem41a** | 1.545749 | 0.001241 |
| **Aaas** | 1.545744 | 0.000672 |
| **Adssl1** | 1.545586 | 0.000178 |
| **Gm13202** | 1.545358 | 0.002405 |
| **Klf15** | 1.544818 | 0.003782 |
| **2410018L13Rik** | 1.54338 | 0.005023 |
| **Dcun1d5** | 1.543367 | 0.002405 |
| **Atpaf2** | 1.542646 | 0.000298 |
| **Chchd7** | 1.54236 | 0.000687 |
| **Pusl1** | 1.542305 | 0.000178 |
| **Gm13015** | 1.541335 | 0.000295 |
| **Rcan1** | 1.541074 | 0.047554 |
| **Mapk3** | 1.540941 | 0.000527 |
| **LOC100503863** | 1.540728 | 0.0018 |
| **Parp16** | 1.539711 | 0.000194 |
| **Rhbdd2** | 1.539035 | 8.61E-05 |
| **Clcn2** | 1.53577 | 0.000411 |
| **Klhl21** | 1.534974 | 0.000532 |
| **Snord47** | 1.534378 | 0.014564 |
| **Sars2** | 1.533887 | 0.000283 |
| **1810063B05Rik** | 1.533723 | 0.00031 |
| **2810453I06Rik** | 1.533064 | 0.0059 |
| **Ntrk2** | 1.532608 | 0.004125 |
| **Acot1** | 1.532548 | 0.000292 |
| **Tmem86a** | 1.532122 | 0.001525 |
| **Slc6a9** | 1.53196 | 0.000652 |
| **Tmem93** | 1.530891 | 0.000672 |
| **Ly6c1** | 1.529753 | 0.028252 |
| **Gm561** | 1.529266 | 0.001166 |
| **Akt1** | 1.529166 | 0.000465 |
| **Kti12** | 1.528865 | 0.00236 |
| **Srm** | 1.526884 | 0.000292 |
| **Hspb8** | 1.526688 | 0.000298 |
| **Eif1b** | 1.526623 | 0.000438 |
| **1110002B05Rik** | 1.525158 | 0.000264 |
| **Tmem37** | 1.52363 | 0.000641 |
| **Cdk2** | 1.522003 | 0.000686 |
| **Gm6181** | 1.521977 | 0.009389 |
| **C1qbp** | 1.521941 | 0.000203 |
| **Tmem63b** | 1.521602 | 0.000532 |
| **Ctdsp2** | 1.520819 | 0.000743 |
| **St3gal4** | 1.520341 | 0.000571 |
| **Zfp36** | 1.519021 | 0.00072 |
| **BC029722** | 1.518991 | 0.000686 |
| **Magoh** | 1.518685 | 0.000858 |
| **Vat1** | 1.518589 | 0.001891 |
| **Tysnd1** | 1.518385 | 0.001217 |
| **Pim1** | 1.516663 | 0.019509 |
| **Bod1** | 1.516631 | 0.000978 |
| **Map2k6** | 1.515135 | 0.005383 |
| **Tm2d2** | 1.513607 | 0.000594 |
| **Nfic** | 1.513281 | 0.000234 |
| **Sfpi1** | 1.512805 | 0.001758 |
| **Rnd1** | 1.512711 | 0.000919 |
| **Scrn2** | 1.512695 | 0.000347 |
| **Rnpep** | 1.512456 | 0.004363 |
| **2010109K11Rik** | 1.510711 | 0.000214 |
| **Mrps18c** | 1.51053 | 0.000267 |
| **2900010M23Rik** | 1.509895 | 0.00038 |
| **Mast3** | 1.509021 | 9.64E-05 |
| **Rpa3** | 1.507868 | 0.001029 |
| **2310061I04Rik** | 1.50751 | 0.000218 |
| **Clec2d** | 1.506816 | 0.017838 |
| **Otud1** | 1.506393 | 0.001829 |
| **Ggt6** | 1.505797 | 0.000267 |
| **Gsto1** | 1.505362 | 0.000607 |
| **Mrpl38** | 1.505041 | 0.00032 |
| **Rhobtb1** | 1.504509 | 0.000283 |
| **Mafg** | 1.503198 | 0.00054 |
| **Chchd4** | 1.502945 | 0.001933 |
| **Serf1** | 1.500165 | 0.000398 |
| **Nt5dc2** | 1.500112 | 0.001438 |
| **Slc9a3r1** | 1.500111 | 0.000724 |
| **Nr2c2** | -1.50014 | 0.000765 |
| **Abcc2** | -1.50017 | 0.001414 |
| **Mx2** | -1.50035 | 0.02359 |
| **Ppp4r1** | -1.5004 | 8.61E-05 |
| **Dpyd** | -1.50087 | 0.009093 |
| **Yes1** | -1.50087 | 0.000544 |
| **Amy1** | -1.50093 | 0.015451 |
| **Oxr1** | -1.50146 | 0.001452 |
| **Xrn1** | -1.50175 | 0.002733 |
| **Cep120** | -1.50239 | 0.001646 |
| **2410002O22Rik** | -1.50275 | 0.001827 |
| **Pex1** | -1.50282 | 0.002445 |
| **Kdm5b** | -1.50292 | 0.005236 |
| **Tcea1** | -1.50294 | 0.001254 |
| **Sp3** | -1.50339 | 0.003903 |
| **Ifih1** | -1.50387 | 0.007922 |
| **Cdh2** | -1.50457 | 0.000283 |
| **Wdr11** | -1.50503 | 0.000251 |
| **Tec** | -1.50508 | 0.003138 |
| **Parp2** | -1.50513 | 0.001069 |
| **2700062C07Rik** | -1.50544 | 0.000755 |
| **Plekha1** | -1.50561 | 0.013683 |
| **Wdr75** | -1.5057 | 0.000631 |
| **Nufip2** | -1.50577 | 0.000297 |
| **Zfhx4** | -1.50583 | 0.000687 |
| **Pfkfb1** | -1.50597 | 0.022411 |
| **Glce** | -1.50673 | 0.002672 |
| **Mocs2** | -1.50689 | 0.000554 |
| **Krr1** | -1.5074 | 0.000308 |
| **Nedd1** | -1.50748 | 0.000964 |
| **Fgd6** | -1.50794 | 0.000309 |
| **Fpgt** | -1.508 | 0.002706 |
| **Gnl3** | -1.50849 | 0.003085 |
| **Cxadr** | -1.50956 | 0.001648 |
| **Dnajc7** | -1.50957 | 0.000724 |
| **Mospd1** | -1.50988 | 0.019879 |
| **Mina** | -1.51009 | 0.000763 |
| **Hus1** | -1.51049 | 0.003205 |
| **Osbp** | -1.51095 | 0.000343 |
| **Lpgat1** | -1.51107 | 0.001262 |
| **Gabra3** | -1.51157 | 0.004299 |
| **Igbp1** | -1.51161 | 0.000527 |
| **Atl3** | -1.51173 | 0.00121 |
| **Calu** | -1.51186 | 0.000444 |
| **Pank3** | -1.51192 | 0.011453 |
| **Arhgap12** | -1.5121 | 0.00052 |
| **Abcd3** | -1.51221 | 0.001674 |
| **Ccnl1** | -1.51223 | 0.003376 |
| **Ulk2** | -1.51238 | 0.000878 |
| **Pde7b** | -1.51292 | 0.00283 |
| **Gpd2** | -1.51317 | 0.002759 |
| **Sos2** | -1.5134 | 0.000337 |
| **Ralgapa2** | -1.51348 | 0.003179 |
| **Msr1** | -1.5135 | 0.000177 |
| **Ap3b1** | -1.51359 | 0.000869 |
| **Tcf4** | -1.5137 | 0.000426 |
| **Rnmt** | -1.51372 | 0.001268 |
| **Cyp39a1** | -1.51388 | 0.001655 |
| **Rlim** | -1.51416 | 0.001375 |
| **Lztfl1** | -1.51419 | 0.002617 |
| **Zfp229** | -1.51451 | 0.006804 |
| **Vps54** | -1.51459 | 9.64E-05 |
| **Acmsd** | -1.51475 | 0.006182 |
| **Snap23** | -1.51483 | 0.000558 |
| **Sec23a** | -1.51577 | 0.001392 |
| **Akr1d1** | -1.51606 | 0.002034 |
| **Ppp1r12a** | -1.51609 | 0.00072 |
| **Utp11l** | -1.51665 | 0.000276 |
| **0610010F05Rik** | -1.5169 | 0.000716 |
| **Mtdh** | -1.51738 | 0.000962 |
| **Dhx15** | -1.51795 | 0.000731 |
| **Ctsc** | -1.51808 | 0.00052 |
| **Phldb2** | -1.51811 | 0.00038 |
| **Arid2** | -1.51818 | 0.00052 |
| **Nars** | -1.51844 | 0.000251 |
| **Akap11** | -1.51852 | 0.002883 |
| **Zfp280c** | -1.51868 | 0.003908 |
| **Suz12** | -1.51875 | 0.000745 |
| **Gbp2** | -1.51896 | 0.002659 |
| **Rabgap1** | -1.51912 | 0.002718 |
| **Gtf3c3** | -1.51936 | 0.01701 |
| **Lin7a** | -1.51953 | 0.010654 |
| **Stk3** | -1.51959 | 0.000949 |
| **Birc2** | -1.51994 | 0.009957 |
| **Mrc1** | -1.52072 | 0.002179 |
| **2610101N10Rik** | -1.5209 | 0.000724 |
| **Dync1i2** | -1.52126 | 0.001722 |
| **Snap29** | -1.52162 | 0.000733 |
| **Zbtb26** | -1.52176 | 0.00103 |
| **Cdc42bpa** | -1.52228 | 0.000667 |
| **D6Wsu163e** | -1.52234 | 0.002203 |
| **Gpkow** | -1.52281 | 0.000295 |
| **Arhgef12** | -1.52285 | 0.000377 |
| **Slc25a36** | -1.52321 | 0.014542 |
| **Nmi** | -1.52349 | 0.001467 |
| **Fbxo11** | -1.52382 | 0.000716 |
| **Brix1** | -1.52413 | 0.007912 |
| **Apol7a** | -1.52535 | 0.006747 |
| **4932438A13Rik** | -1.52559 | 0.001392 |
| **Rp2h** | -1.52572 | 0.005693 |
| **Setd5** | -1.52619 | 0.000297 |
| **Vipar** | -1.52654 | 0.000443 |
| **Ero1l** | -1.52663 | 0.000907 |
| **Ppm1k** | -1.52671 | 0.004263 |
| **Zfp277** | -1.5271 | 0.001691 |
| **Fnbp4** | -1.52711 | 0.000309 |
| **Sort1** | -1.52733 | 0.011538 |
| **4833420G17Rik** | -1.52771 | 0.001251 |
| **Tapt1** | -1.52803 | 0.000244 |
| **Usp24** | -1.52815 | 0.000557 |
| **Znhit6** | -1.52832 | 0.001117 |
| **Nsmce2** | -1.52924 | 0.001092 |
| **Atl2** | -1.52937 | 0.001123 |
| **Nr5a2** | -1.52949 | 0.000184 |
| **Hipk1** | -1.52952 | 0.000192 |
| **Zfp954** | -1.52963 | 0.000515 |
| **Lonp2** | -1.52973 | 0.000502 |
| **Klhl28** | -1.53024 | 0.000309 |
| **Fig4** | -1.53086 | 0.000298 |
| **Nr3c2** | -1.53087 | 0.001377 |
| **Rad17** | -1.53111 | 0.00168 |
| **Parg** | -1.53115 | 0.001259 |
| **Hcfc2** | -1.53119 | 0.001758 |
| **2610507B11Rik** | -1.53121 | 0.000527 |
| **Srd5a2** | -1.53167 | 0.000582 |
| **Casd1** | -1.53277 | 0.00893 |
| **Zfp329** | -1.5328 | 0.001 |
| **Tjp1** | -1.53286 | 0.002405 |
| **Uprt** | -1.53286 | 0.002799 |
| **Exoc4** | -1.53348 | 0.002979 |
| **Nfx1** | -1.53376 | 0.000298 |
| **9030624J02Rik** | -1.53387 | 0.000178 |
| **Ncapd3** | -1.53503 | 0.002164 |
| **Zdhhc13** | -1.53519 | 0.008193 |
| **Eif2s2** | -1.53533 | 0.008992 |
| **Ltn1** | -1.53547 | 0.005605 |
| **Cyp7a1** | -1.53629 | 0.047764 |
| **Nr2c1** | -1.53706 | 0.001717 |
| **Ppargc1a** | -1.53729 | 0.000957 |
| **Adam10** | -1.53773 | 0.000983 |
| **Ctcf** | -1.5396 | 0.00038 |
| **Nsmaf** | -1.5397 | 0.001676 |
| **Dnajc13** | -1.54014 | 0.001761 |
| **Vcl** | -1.54097 | 0.000283 |
| **Vps36** | -1.54126 | 0.000453 |
| **Pja1** | -1.54168 | 0.000178 |
| **Papss2** | -1.54206 | 0.002001 |
| **Rap1b** | -1.54263 | 0.000598 |
| **Tdo2** | -1.54277 | 0.001166 |
| **Megf9** | -1.54288 | 0.000367 |
| **Eef1a1** | -1.54393 | 0.000368 |
| **AI848100** | -1.54462 | 0.003341 |
| **Med23** | -1.54466 | 0.000599 |
| **Snord14e** | -1.54471 | 0.008783 |
| **Thap6** | -1.54484 | 0.00077 |
| **Ogg1** | -1.54491 | 0.000153 |
| **Srsf11** | -1.545 | 0.000341 |
| **Ube3a** | -1.54514 | 0.000808 |
| **Traf6** | -1.54566 | 0.000556 |
| **Fem1c** | -1.54592 | 0.000306 |
| **Ankib1** | -1.54682 | 0.000244 |
| **Klhl7** | -1.54732 | 0.002653 |
| **Trip12** | -1.54767 | 0.000512 |
| **Cct2** | -1.54804 | 0.000317 |
| **Pik3c2g** | -1.54815 | 0.006895 |
| **Ik** | -1.54821 | 0.002487 |
| **Klraq1** | -1.54888 | 0.000177 |
| **1110057K04Rik** | -1.54896 | 0.000234 |
| **Ddx50** | -1.54905 | 0.003075 |
| **Taf1d** | -1.54946 | 0.011519 |
| **Arcn1** | -1.55038 | 0.000945 |
| **Inhba** | -1.55079 | 0.00213 |
| **Cyld** | -1.55082 | 0.001729 |
| **Nufip1** | -1.55152 | 0.000783 |
| **Pign** | -1.55178 | 0.000661 |
| **Enpp1** | -1.55182 | 0.000537 |
| **Kpnb1** | -1.5519 | 0.000418 |
| **Atp11c** | -1.55212 | 0.005219 |
| **Qser1** | -1.55243 | 0.000729 |
| **Ptprk** | -1.55279 | 0.000298 |
| **Cfh** | -1.55312 | 0.011037 |
| **Hspa4** | -1.55316 | 0.000702 |
| **Arl5a** | -1.55317 | 0.000533 |
| **LOC100502885** | -1.55384 | 0.002375 |
| **Sdr9c7** | -1.55418 | 0.031396 |
| **Map4k5** | -1.55431 | 0.001103 |
| **C030016D13Rik** | -1.5544 | 0.006524 |
| **Fam134b** | -1.55465 | 0.005476 |
| **Ripk1** | -1.55474 | 0.000662 |
| **Lyn** | -1.55475 | 0.000171 |
| **Fmn1** | -1.5558 | 0.007631 |
| **Ift52** | -1.55624 | 0.000587 |
| **Gna13** | -1.55634 | 0.000291 |
| **Abcb4** | -1.55642 | 0.00038 |
| **Ankle2** | -1.55651 | 0.000506 |
| **Bfar** | -1.55655 | 0.000244 |
| **Tbl1x** | -1.55665 | 0.000343 |
| **Shoc2** | -1.55769 | 0.000984 |
| **Trmt5** | -1.55776 | 0.000919 |
| **Prpf40a** | -1.55787 | 0.004261 |
| **Agphd1** | -1.55792 | 0.0013 |
| **Atp2b1** | -1.55793 | 0.000518 |
| **Ppp1r7** | -1.55799 | 0.000533 |
| **Dynlt3** | -1.55816 | 0.002227 |
| **Igsf5** | -1.55831 | 0.000283 |
| **Atrx** | -1.55843 | 0.001887 |
| **Zkscan1** | -1.55854 | 0.002119 |
| **Zbtb11** | -1.55931 | 0.000446 |
| **Epb4.1l5** | -1.55954 | 0.000298 |
| **Usp48** | -1.55977 | 0.000716 |
| **Jak2** | -1.56006 | 0.002198 |
| **Siae** | -1.56026 | 0.000673 |
| **Dars2** | -1.5606 | 0.000494 |
| **Rsbn1l** | -1.5606 | 0.000292 |
| **3110043O21Rik** | -1.56061 | 0.005265 |
| **Armc1** | -1.56095 | 0.000493 |
| **Thumpd3** | -1.56098 | 0.001 |
| **Pik3r1** | -1.56163 | 0.00073 |
| **Ranbp2** | -1.56187 | 0.002449 |
| **Pcm1** | -1.56199 | 0.009068 |
| **Zfp281** | -1.56222 | 0.000303 |
| **Fam136a** | -1.5628 | 0.011755 |
| **Zfyve16** | -1.56306 | 0.001529 |
| **Rsad2** | -1.56377 | 0.011751 |
| **Phka2** | -1.56382 | 0.00054 |
| **Copa** | -1.56404 | 0.000185 |
| **Xpo4** | -1.56424 | 0.000724 |
| **Kmo** | -1.56449 | 0.000536 |
| **Snx14** | -1.56458 | 0.002664 |
| **Zfp595** | -1.56516 | 0.005538 |
| **Guf1** | -1.56617 | 0.001357 |
| **Fam48a** | -1.56656 | 0.000974 |
| **Psma1** | -1.56678 | 0.003688 |
| **Nr1d2** | -1.56724 | 0.002763 |
| **Gm4952** | -1.56791 | 0.00028 |
| **LOC100038947** | -1.56874 | 0.010821 |
| **Taf4b** | -1.56887 | 0.000178 |
| **Larp4b** | -1.56889 | 0.000693 |
| **Smg1** | -1.5696 | 0.002642 |
| **Senp6** | -1.56973 | 0.000607 |
| **Nbr1** | -1.56995 | 0.000335 |
| **Phf6** | -1.57015 | 0.002832 |
| **Arap2** | -1.57019 | 0.002653 |
| **Mak16** | -1.57025 | 0.000652 |
| **Serpina3m** | -1.57029 | 0.003279 |
| **Rsrc2** | -1.57047 | 0.000483 |
| **Usp14** | -1.57067 | 0.001464 |
| **Utp6** | -1.57151 | 0.000592 |
| **Stxbp3a** | -1.57217 | 0.002898 |
| **Tomm70a** | -1.57221 | 0.004702 |
| **Ankhd1** | -1.57223 | 0.002045 |
| **Pias1** | -1.57242 | 0.000832 |
| **Gbp3** | -1.57263 | 0.003827 |
| **Usp46** | -1.57266 | 0.000649 |
| **Bivm** | -1.57273 | 0.004524 |
| **Lpp** | -1.57304 | 0.001449 |
| **Cacybp** | -1.57318 | 0.00043 |
| **Gpr110** | -1.57319 | 0.017033 |
| **Masp1** | -1.57336 | 0.000339 |
| **Srpk1** | -1.57397 | 0.000177 |
| **Mlh3** | -1.57411 | 0.000971 |
| **Cct3** | -1.57475 | 0.000596 |
| **Rnf19a** | -1.57503 | 0.001124 |
| **Ccdc47** | -1.57581 | 0.009265 |
| **Cct6a** | -1.57629 | 0.000514 |
| **Mrpl1** | -1.57669 | 0.002069 |
| **Hps3** | -1.57726 | 0.00054 |
| **Ythdc1** | -1.57742 | 0.001126 |
| **Hook1** | -1.57764 | 0.019998 |
| **Nfat5** | -1.5782 | 0.001284 |
| **Zfp148** | -1.57867 | 0.001458 |
| **Ddx6** | -1.57872 | 0.000587 |
| **Tgfbr1** | -1.57926 | 0.004937 |
| **Ivns1abp** | -1.57941 | 0.017384 |
| **Ube2v2** | -1.57959 | 0.002673 |
| **2210404J11Rik** | -1.57988 | 0.00117 |
| **Raph1** | -1.5802 | 0.000177 |
| **Evi5** | -1.58026 | 0.001186 |
| **Tdrd7** | -1.58032 | 0.000358 |
| **Psmd12** | -1.58109 | 0.000656 |
| **Sorbs2** | -1.58131 | 0.000171 |
| **Smc5** | -1.58147 | 0.001636 |
| **Krt18** | -1.5815 | 0.000603 |
| **Dcun1d1** | -1.58309 | 0.001189 |
| **Ncbp1** | -1.58317 | 0.000664 |
| **Capn2** | -1.58358 | 9.64E-05 |
| **Mtpap** | -1.58372 | 0.00079 |
| **Anapc1** | -1.58373 | 0.000527 |
| **Ell2** | -1.584 | 0.000705 |
| **Wac** | -1.58427 | 0.000531 |
| **Syt1** | -1.58464 | 0.005449 |
| **Stambp** | -1.58484 | 0.000468 |
| **Zfyve9** | -1.58582 | 0.000496 |
| **Gclc** | -1.58602 | 0.001364 |
| **Zfp871** | -1.58658 | 0.004279 |
| **2310035C23Rik** | -1.58669 | 0.000298 |
| **Nab1** | -1.58688 | 0.000366 |
| **Rbm5** | -1.58692 | 0.000519 |
| **Kpna1** | -1.58713 | 0.001209 |
| **Atxn3** | -1.58727 | 0.000884 |
| **Nmt1** | -1.58783 | 0.000184 |
| **Tlk1** | -1.58819 | 0.000276 |
| **Pid1** | -1.58853 | 0.000497 |
| **Usp32** | -1.59 | 0.001645 |
| **Pyroxd1** | -1.59013 | 0.000384 |
| **Uba6** | -1.59056 | 0.006381 |
| **Cdkl5** | -1.59103 | 0.001689 |
| **Stat1** | -1.59107 | 0.000983 |
| **Rc3h1** | -1.59197 | 0.000309 |
| **Kpna4** | -1.59277 | 0.000502 |
| **Mbnl2** | -1.59339 | 0.002718 |
| **Herc6** | -1.5938 | 0.001026 |
| **Dicer1** | -1.59391 | 0.001347 |
| **Gpsm2** | -1.59424 | 0.00038 |
| **Uso1** | -1.59439 | 0.000298 |
| **Cd2ap** | -1.59494 | 0.000736 |
| **Fam69a** | -1.59519 | 0.00054 |
| **C9** | -1.59632 | 0.003423 |
| **Dennd4a** | -1.5967 | 0.002001 |
| **Hc** | -1.59685 | 0.002829 |
| **Azi2** | -1.59771 | 0.000264 |
| **Cpne3** | -1.59876 | 0.003534 |
| **Ttc37** | -1.5988 | 0.000849 |
| **AW209491** | -1.59934 | 0.000347 |
| **Ppp1r3c** | -1.60009 | 0.00165 |
| **Ubr1** | -1.60011 | 0.000368 |
| **A130022J15Rik** | -1.60081 | 0.002113 |
| **Arhgef3** | -1.60091 | 0.000463 |
| **Smarcad1** | -1.6011 | 0.001241 |
| **Helb** | -1.60119 | 0.000472 |
| **Gcfc1** | -1.60146 | 0.000194 |
| **Rapgef5** | -1.60157 | 0.000985 |
| **Slk** | -1.60235 | 0.001322 |
| **Setdb2** | -1.60263 | 0.005906 |
| **Mdh1** | -1.60269 | 0.000705 |
| **Tmem144** | -1.60286 | 0.000238 |
| **Zmynd11** | -1.6029 | 0.000184 |
| **Pik3c2a** | -1.60313 | 0.002033 |
| **Cpsf3** | -1.60327 | 0.000283 |
| **4732418C07Rik** | -1.60385 | 0.000335 |
| **Palmd** | -1.60388 | 0.001231 |
| **Cldn2** | -1.60423 | 0.00032 |
| **Senp5** | -1.60442 | 0.000335 |
| **Rfc4** | -1.60446 | 0.001405 |
| **Dsc2** | -1.60579 | 0.003982 |
| **Hectd1** | -1.6066 | 0.000656 |
| **Xrcc4** | -1.60681 | 0.002289 |
| **Rspry1** | -1.60754 | 0.000251 |
| **Fmr1** | -1.60806 | 0.000554 |
| **Plin2** | -1.60815 | 0.005466 |
| **Ipo5** | -1.60902 | 0.000633 |
| **Usp47** | -1.60914 | 0.00286 |
| **AI182371** | -1.60917 | 0.000423 |
| **Mpp6** | -1.60928 | 0.000317 |
| **Exoc3** | -1.60998 | 0.000945 |
| **Fgfr1op2** | -1.61003 | 0.000298 |
| **Rel** | -1.61003 | 0.000478 |
| **Plcxd2** | -1.61045 | 0.024478 |
| **Adam17** | -1.61074 | 0.000218 |
| **Lipc** | -1.61078 | 0.000298 |
| **Usp40** | -1.61155 | 0.002573 |
| **Rsbn1** | -1.61215 | 0.000494 |
| **Spag9** | -1.61238 | 0.002018 |
| **Cldn1** | -1.61271 | 0.000295 |
| **Prpf4b** | -1.61277 | 0.00048 |
| **Atp6v1a** | -1.61299 | 0.00028 |
| **Ranbp6** | -1.61411 | 0.000476 |
| **Naa25** | -1.61413 | 0.000251 |
| **Zfp280d** | -1.61414 | 0.001665 |
| **Insig2** | -1.61433 | 0.004414 |
| **Vim** | -1.61441 | 0.008054 |
| **Itih2** | -1.6148 | 0.000724 |
| **Ptcd3** | -1.61481 | 0.005974 |
| **Fgd4** | -1.61496 | 0.000656 |
| **Krit1** | -1.61506 | 0.000219 |
| **Il13ra1** | -1.61516 | 0.012134 |
| **Gtdc1** | -1.61613 | 0.006429 |
| **Anapc4** | -1.61638 | 0.000814 |
| **Clint1** | -1.61653 | 8.61E-05 |
| **Tars** | -1.61716 | 0.000153 |
| **Atp6v1c1** | -1.61767 | 0.000463 |
| **Lrp6** | -1.61767 | 0.000298 |
| **Ppp6r3** | -1.61788 | 0.00054 |
| **Pgap1** | -1.61799 | 0.001251 |
| **Calcrl** | -1.61809 | 0.00066 |
| **Atp6v1e1** | -1.61849 | 0.000562 |
| **Grpel2** | -1.6189 | 0.001509 |
| **Pot1a** | -1.61944 | 0.002586 |
| **Rhot1** | -1.62026 | 0.000842 |
| **Zfp68** | -1.62042 | 0.001046 |
| **Slc43a3** | -1.62047 | 0.000153 |
| **Col4a3bp** | -1.6215 | 0.000477 |
| **Btaf1** | -1.6215 | 0.000444 |
| **Eif2a** | -1.62196 | 0.002042 |
| **Sbno1** | -1.62202 | 0.000267 |
| **Enpp4** | -1.6221 | 0.000544 |
| **Elp2** | -1.62277 | 0.000587 |
| **Mmgt1** | -1.62289 | 0.000533 |
| **Olfm3** | -1.6233 | 0.005178 |
| **Fyttd1** | -1.62382 | 0.001107 |
| **1200011I18Rik** | -1.62505 | 0.000171 |
| **Cog5** | -1.62634 | 0.000875 |
| **Erbb2ip** | -1.6265 | 0.00071 |
| **Psmd5** | -1.62674 | 0.000523 |
| **Nol11** | -1.62686 | 0.0005 |
| **Klkb1** | -1.62754 | 0.001796 |
| **Tek** | -1.62762 | 0.000377 |
| **Esyt2** | -1.62858 | 0.000791 |
| **Erap1** | -1.62892 | 0.00239 |
| **Klf7** | -1.6295 | 0.000317 |
| **Fh1** | -1.62972 | 0.000672 |
| **Ttc39b** | -1.63007 | 0.000623 |
| **Dcaf13** | -1.63047 | 0.001761 |
| **Ascc3** | -1.63087 | 0.001489 |
| **Alcam** | -1.6316 | 0.001029 |
| **Fam179b** | -1.63163 | 0.001165 |
| **Fnbp1l** | -1.63176 | 0.000586 |
| **Nf1** | -1.63182 | 0.00143 |
| **Fbxl5** | -1.63202 | 0.004363 |
| **Lancl2** | -1.63223 | 0.000453 |
| **Tbck** | -1.6326 | 0.000244 |
| **Mapk9** | -1.63292 | 0.000251 |
| **Snx4** | -1.63364 | 0.000339 |
| **Fam82a1** | -1.63449 | 0.000276 |
| **Tom1l1** | -1.6352 | 0.000244 |
| **Rnf13** | -1.63548 | 0.001604 |
| **Ednrb** | -1.63631 | 0.000531 |
| **Pdik1l** | -1.63678 | 0.000177 |
| **G2e3** | -1.63683 | 0.00045 |
| **Slu7** | -1.63749 | 0.00268 |
| **2810007J24Rik** | -1.63791 | 0.010013 |
| **Appl2** | -1.63802 | 0.000251 |
| **Cyp2c70** | -1.63896 | 0.016294 |
| **Gabpa** | -1.6394 | 0.001239 |
| **Mlkl** | -1.6397 | 0.014431 |
| **Il1rap** | -1.63985 | 0.000781 |
| **Twf1** | -1.64103 | 0.000416 |
| **Tcf12** | -1.64111 | 0.000463 |
| **Xrn2** | -1.64178 | 0.003537 |
| **AI314180** | -1.64228 | 0.000623 |
| **Tcfec** | -1.64273 | 0.001231 |
| **Arhgap42** | -1.64336 | 0.000499 |
| **Ddx46** | -1.64396 | 0.000317 |
| **1810030O07Rik** | -1.64411 | 0.00079 |
| **Usp9x** | -1.64424 | 0.001247 |
| **Nmt2** | -1.64456 | 0.001552 |
| **Xpo7** | -1.64461 | 0.000283 |
| **Snx5** | -1.64518 | 0.00114 |
| **Entpd4** | -1.64528 | 7.72E-05 |
| **Mrrf** | -1.6459 | 8.61E-05 |
| **Rbbp9** | -1.64681 | 0.000372 |
| **Ate1** | -1.64684 | 0.000303 |
| **Bmpr2** | -1.64714 | 0.000298 |
| **C1galt1** | -1.64991 | 0.001049 |
| **Stx12** | -1.6501 | 0.002133 |
| **Trp53inp1** | -1.65021 | 0.00182 |
| **Trip4** | -1.65043 | 0.000297 |
| **Rb1cc1** | -1.65095 | 0.012817 |
| **Calm1** | -1.6512 | 0.000244 |
| **Riok3** | -1.65154 | 0.000785 |
| **Uggt1** | -1.65163 | 0.000267 |
| **Lats1** | -1.65199 | 9.64E-05 |
| **Zfp943** | -1.65475 | 0.001939 |
| **4931406C07Rik** | -1.65519 | 0.000527 |
| **Nfatc3** | -1.65577 | 0.000177 |
| **Gpr116** | -1.65578 | 0.000958 |
| **Papolg** | -1.65589 | 0.001397 |
| **Mettl4** | -1.65613 | 0.008077 |
| **Epm2aip1** | -1.65623 | 0.001253 |
| **Ogt** | -1.65676 | 0.002109 |
| **Plaa** | -1.65744 | 0.000613 |
| **Usp37** | -1.65773 | 0.000672 |
| **Srr** | -1.65775 | 0.000264 |
| **BC003331** | -1.65813 | 0.004326 |
| **Gbp4** | -1.6584 | 0.002845 |
| **Psmd14** | -1.65858 | 0.001731 |
| **3010026O09Rik** | -1.65911 | 0.000317 |
| **Matr3** | -1.65931 | 0.002219 |
| **Hspa5** | -1.66037 | 0.000177 |
| **Dld** | -1.66081 | 0.001988 |
| **Ints4** | -1.66125 | 0.000254 |
| **Rsl1** | -1.66176 | 0.003126 |
| **Copb2** | -1.66186 | 0.000444 |
| **Golph3l** | -1.66256 | 0.000687 |
| **Lmbrd2** | -1.66269 | 0.000587 |
| **Zfr** | -1.66293 | 0.000214 |
| **Tpp2** | -1.66359 | 0.00118 |
| **Slc23a1** | -1.66426 | 0.000933 |
| **Dmxl2** | -1.6643 | 0.000341 |
| **Ubr3** | -1.66522 | 0.000977 |
| **Papss1** | -1.66561 | 0.000185 |
| **Cops3** | -1.66583 | 0.001182 |
| **Cfi** | -1.66592 | 0.000687 |
| **Defb1** | -1.66625 | 0.021966 |
| **8430410K20Rik** | -1.66728 | 0.000177 |
| **Serpina3k** | -1.66776 | 0.000366 |
| **Wdr48** | -1.66821 | 0.000295 |
| **Ralgapb** | -1.66825 | 0.000175 |
| **Strn** | -1.66923 | 0.000244 |
| **Fcho2** | -1.67013 | 0.002143 |
| **Malt1** | -1.67025 | 0.000186 |
| **Ssb** | -1.6707 | 0.004308 |
| **Avpr1a** | -1.67167 | 0.038081 |
| **Rb1** | -1.67216 | 0.000741 |
| **Lrrc40** | -1.67242 | 0.000672 |
| **Nbeal1** | -1.67246 | 0.002161 |
| **Ppp2r1b** | -1.67256 | 0.000335 |
| **Myo6** | -1.67283 | 0.000464 |
| **Tnpo1** | -1.673 | 0.002304 |
| **Sec62** | -1.67353 | 0.000192 |
| **Uba2** | -1.67411 | 0.000214 |
| **Aox3** | -1.67447 | 0.002211 |
| **Lpar6** | -1.67574 | 0.005474 |
| **Ints6** | -1.67599 | 0.000251 |
| **Tubgcp4** | -1.67676 | 0.000171 |
| **6330407A03Rik** | -1.67726 | 0.000454 |
| **Zc3h11a** | -1.67779 | 0.000605 |
| **Eif1a** | -1.6784 | 0.001063 |
| **Aqp4** | -1.67856 | 0.000368 |
| **Msl3** | -1.67881 | 0.000351 |
| **Osbpl1a** | -1.67914 | 0.000569 |
| **Sh3bgrl2** | -1.67946 | 0.000933 |
| **Itch** | -1.67958 | 0.000562 |
| **Mid2** | -1.67986 | 0.000268 |
| **Rictor** | -1.67995 | 0.001347 |
| **Wapal** | -1.68019 | 0.001468 |
| **Zfp84** | -1.68031 | 0.000213 |
| **Cops5** | -1.68073 | 0.000687 |
| **Gca** | -1.68076 | 0.002779 |
| **Ggps1** | -1.68127 | 0.000297 |
| **Irf6** | -1.6826 | 0.001209 |
| **Abcg2** | -1.68303 | 0.000755 |
| **Stag2** | -1.68331 | 0.002799 |
| **Orc5** | -1.68395 | 0.002495 |
| **Pls3** | -1.68494 | 0.002012 |
| **1110008L16Rik** | -1.68543 | 0.000402 |
| **Arrdc3** | -1.68561 | 0.049798 |
| **Ipo11** | -1.68563 | 0.002673 |
| **Zzz3** | -1.68567 | 0.001284 |
| **Mef2a** | -1.68578 | 0.000455 |
| **Csrp3** | -1.68766 | 0.002768 |
| **Mbtd1** | -1.68773 | 0.002815 |
| **Otud6b** | -1.68939 | 0.000724 |
| **Dis3** | -1.6895 | 0.000396 |
| **Atp11b** | -1.69122 | 0.000949 |
| **Pgm3** | -1.69165 | 0.005354 |
| **Trpm7** | -1.69184 | 0.005184 |
| **Sntb1** | -1.69228 | 0.000743 |
| **Abca8b** | -1.69369 | 0.003584 |
| **Vps37a** | -1.69395 | 0.000372 |
| **EG214403** | -1.69416 | 0.006636 |
| **Slc17a4** | -1.69454 | 0.004052 |
| **Pdilt** | -1.69499 | 0.002573 |
| **Dpy19l4** | -1.6957 | 0.000687 |
| **Spon2** | -1.69573 | 0.015197 |
| **Mtmr6** | -1.69592 | 0.000465 |
| **Dmxl1** | -1.69602 | 0.001331 |
| **Rab11fip2** | -1.69623 | 0.003239 |
| **Lgals8** | -1.69631 | 0.000172 |
| **N4bp2l2** | -1.69722 | 0.000434 |
| **Exoc6b** | -1.69781 | 0.000478 |
| **Avl9** | -1.6979 | 0.000664 |
| **Rassf6** | -1.69818 | 0.000359 |
| **Phkb** | -1.69855 | 0.00059 |
| **Aldh8a1** | -1.69863 | 0.000138 |
| **Trim33** | -1.69948 | 0.000405 |
| **A230046K03Rik** | -1.70038 | 0.001524 |
| **Colec12** | -1.70072 | 0.000516 |
| **Secisbp2l** | -1.70101 | 0.000417 |
| **Pdia4** | -1.70121 | 0.000199 |
| **Ipo8** | -1.70177 | 0.000178 |
| **Zc3h12c** | -1.70285 | 0.000257 |
| **Mgam** | -1.70306 | 0.000738 |
| **Epc2** | -1.70344 | 0.000644 |
| **Arsk** | -1.70363 | 0.000919 |
| **Mapk6** | -1.70375 | 0.000276 |
| **Eml4** | -1.70397 | 0.000178 |
| **Mudeng** | -1.70442 | 0.001878 |
| **Tnrc6a** | -1.70465 | 0.000309 |
| **Atpbd4** | -1.70492 | 0.000225 |
| **7-Mar** | -1.70633 | 0.001767 |
| **Etf1** | -1.7073 | 0.000446 |
| **Cpeb4** | -1.70778 | 0.001086 |
| **Tpmt** | -1.70843 | 0.000645 |
| **Mia2** | -1.70847 | 0.001639 |
| **Slc10a5** | -1.70915 | 0.001092 |
| **Brcc3** | -1.70978 | 0.000835 |
| **Mpp5** | -1.71001 | 0.001274 |
| **7-Sep** | -1.71033 | 0.002203 |
| **Zbtb33** | -1.71047 | 0.000317 |
| **Uspl1** | -1.71096 | 0.000472 |
| **Xrcc5** | -1.71119 | 0.000755 |
| **Trim23** | -1.71134 | 0.005007 |
| **Ccdc25** | -1.71151 | 0.001389 |
| **Usp7** | -1.7116 | 0.000268 |
| **Slc30a4** | -1.71211 | 0.000391 |
| **Cdc27** | -1.71257 | 0.000725 |
| **Tnfsf10** | -1.7126 | 0.000662 |
| **Map4k3** | -1.71398 | 0.000463 |
| **Fam135a** | -1.71488 | 0.00049 |
| **Znrd1as** | -1.71545 | 0.002236 |
| **Pbrm1** | -1.71576 | 0.001092 |
| **Pde4b** | -1.71646 | 0.001354 |
| **Mtf2** | -1.71675 | 0.000419 |
| **Jhdm1d** | -1.71732 | 0.00079 |
| **Pdxdc1** | -1.71737 | 0.000276 |
| **Pde9a** | -1.71761 | 8.61E-05 |
| **Aqp8** | -1.71905 | 0.031218 |
| **Trim2** | -1.71914 | 0.000706 |
| **1110018G07Rik** | -1.71936 | 0.000213 |
| **Rnf141** | -1.7229 | 0.000298 |
| **Stx17** | -1.72379 | 0.001129 |
| **Cdadc1** | -1.72458 | 0.000298 |
| **Zfc3h1** | -1.72481 | 0.000292 |
| **Ubxn4** | -1.72492 | 0.000171 |
| **Impact** | -1.7252 | 0.001182 |
| **Arhgap18** | -1.7254 | 0.00071 |
| **Tbl1xr1** | -1.72541 | 0.00084 |
| **Scyl2** | -1.72578 | 0.000308 |
| **Farsb** | -1.7265 | 0.001331 |
| **Zfp72** | -1.7276 | 0.00072 |
| **Usp16** | -1.73026 | 0.001469 |
| **Lrpprc** | -1.73045 | 0.000677 |
| **Bbs10** | -1.7311 | 0.000615 |
| **1810074P20Rik** | -1.73149 | 0.00393 |
| **Eltd1** | -1.73168 | 0.000317 |
| **Stk4** | -1.73185 | 0.000153 |
| **Acad11** | -1.73262 | 0.000303 |
| **Wnk1** | -1.73273 | 0.000193 |
| **Zfp809** | -1.73274 | 0.001801 |
| **Ankfy1** | -1.73312 | 0.000192 |
| **Cyp2c67** | -1.73344 | 0.005727 |
| **Larp4** | -1.73346 | 0.00673 |
| **Ptpn12** | -1.73548 | 0.000194 |
| **Sepsecs** | -1.73566 | 0.000838 |
| **St7l** | -1.73595 | 0.000623 |
| **Sfrs18** | -1.73601 | 0.000652 |
| **Atp2c1** | -1.73629 | 0.000324 |
| **Vps39** | -1.73649 | 8.61E-05 |
| **Fnip1** | -1.73692 | 0.001414 |
| **Tbc1d8b** | -1.73727 | 0.001288 |
| **2-Sep** | -1.73734 | 0.001509 |
| **Opa1** | -1.73798 | 0.00216 |
| **Eif4g2** | -1.73933 | 0.000298 |
| **Usp45** | -1.74022 | 0.000496 |
| **Arntl** | -1.74108 | 0.001636 |
| **Eif2s1** | -1.74141 | 0.000974 |
| **Strbp** | -1.74145 | 0.000928 |
| **Rdx** | -1.74173 | 0.001092 |
| **Sel1l** | -1.74267 | 0.000178 |
| **Zcchc6** | -1.74584 | 0.000458 |
| **Serpinb8** | -1.74612 | 0.003015 |
| **Pion** | -1.74682 | 0.001302 |
| **Srek1ip1** | -1.74689 | 0.002374 |
| **Frmd4b** | -1.74797 | 0.006051 |
| **Mdm2** | -1.74879 | 0.000649 |
| **Nsun3** | -1.74961 | 0.000416 |
| **Clk4** | -1.74962 | 0.000352 |
| **Anubl1** | -1.75186 | 0.014799 |
| **Dlg1** | -1.75251 | 0.000192 |
| **Clk1** | -1.75283 | 0.001884 |
| **Gbp8** | -1.75285 | 0.003022 |
| **Cnot6** | -1.7533 | 0.000632 |
| **Otud4** | -1.75364 | 0.000106 |
| **Dtx3l** | -1.75463 | 0.000172 |
| **Abca6** | -1.75465 | 0.001681 |
| **Blzf1** | -1.75533 | 0.000303 |
| **Gramd1c** | -1.7573 | 0.00031 |
| **Rasa1** | -1.75772 | 0.000607 |
| **Pcf11** | -1.75816 | 0.00038 |
| **Birc6** | -1.75852 | 0.000755 |
| **Eif5** | -1.75865 | 0.000177 |
| **Hook3** | -1.75894 | 0.001677 |
| **Lsg1** | -1.75983 | 0.000203 |
| **Gzf1** | -1.76093 | 0.000537 |
| **Phf20l1** | -1.76215 | 0.000418 |
| **Copg2** | -1.76271 | 0.000267 |
| **Dnajc10** | -1.76406 | 0.000533 |
| **Sp100** | -1.76407 | 0.000276 |
| **Usp33** | -1.76448 | 0.000554 |
| **Sacm1l** | -1.76493 | 0.000453 |
| **Cfd** | -1.76498 | 0.006457 |
| **Dpp8** | -1.76528 | 0.000218 |
| **Mbnl1** | -1.7654 | 0.000494 |
| **Lman1** | -1.76593 | 0.000171 |
| **B3galt1** | -1.76632 | 0.028104 |
| **Hsdl2** | -1.76648 | 0.000526 |
| **Dcn** | -1.76652 | 0.000519 |
| **Fam114a1** | -1.7667 | 0.000189 |
| **Bche** | -1.76777 | 0.012833 |
| **F13b** | -1.76895 | 0.000527 |
| **Kif5b** | -1.76932 | 0.000751 |
| **Zc3h14** | -1.76953 | 0.000177 |
| **Lysmd3** | -1.77037 | 0.000251 |
| **Ppwd1** | -1.77058 | 0.002001 |
| **Osbpl8** | -1.77257 | 0.000299 |
| **Exoc2** | -1.77337 | 0.000192 |
| **Nostrin** | -1.77389 | 0.003182 |
| **Zfp26** | -1.77535 | 0.001833 |
| **Ppil4** | -1.77583 | 0.000298 |
| **Mios** | -1.77678 | 0.000417 |
| **Tcp1** | -1.77702 | 0.000583 |
| **Mtif2** | -1.77782 | 0.000563 |
| **Trappc8** | -1.77845 | 0.000851 |
| **Cdc37l1** | -1.77924 | 0.001841 |
| **Osgepl1** | -1.7796 | 0.000817 |
| **Pparg** | -1.77977 | 0.013714 |
| **Nt5e** | -1.78115 | 0.008211 |
| **Car1** | -1.78265 | 0.004076 |
| **Depdc7** | -1.78269 | 0.000552 |
| **Atf2** | -1.78374 | 0.000539 |
| **Mtmr10** | -1.78396 | 0.000317 |
| **Dctn4** | -1.78419 | 0.000283 |
| **Atrn** | -1.78444 | 0.000171 |
| **Deptor** | -1.78454 | 0.000351 |
| **Nudcd1** | -1.78469 | 0.000309 |
| **Exosc9** | -1.78537 | 0.000519 |
| **Nqo2** | -1.78582 | 0.000996 |
| **Map2k4** | -1.78593 | 0.00052 |
| **Hipk3** | -1.78608 | 0.000251 |
| **Iws1** | -1.78615 | 0.000533 |
| **Cald1** | -1.7864 | 8.61E-05 |
| **Vwa5a** | -1.78641 | 0.001132 |
| **Plrg1** | -1.78655 | 0.000292 |
| **Bclaf1** | -1.78664 | 0.000308 |
| **Optn** | -1.78667 | 0.000281 |
| **Tbc1d15** | -1.78699 | 0.00068 |
| **Mynn** | -1.78714 | 0.000298 |
| **Tax1bp1** | -1.7881 | 0.002966 |
| **Elovl3** | -1.79062 | 0.000672 |
| **4921513D23Rik** | -1.7913 | 0.000289 |
| **LOC100503489** | -1.79161 | 0.000412 |
| **Dcun1d4** | -1.79409 | 8.61E-05 |
| **Luc7l3** | -1.79508 | 0.000823 |
| **Tnpo3** | -1.79553 | 0.000177 |
| **Zfp654** | -1.79615 | 0.000724 |
| **Cct4** | -1.7965 | 0.000298 |
| **Ublcp1** | -1.79687 | 0.004538 |
| **Casp8** | -1.797 | 0.000533 |
| **Ggnbp2** | -1.79743 | 0.00117 |
| **Fxr1** | -1.79776 | 0.001092 |
| **Zfp317** | -1.79967 | 0.000562 |
| **Cnot1** | -1.80037 | 0.000709 |
| **Tgoln1** | -1.80091 | 0.000352 |
| **Atp8b1** | -1.80182 | 0.000297 |
| **Cul5** | -1.80186 | 0.001251 |
| **Prepl** | -1.80219 | 0.000218 |
| **Taf2** | -1.8024 | 0.000459 |
| **Mut** | -1.80333 | 0.001182 |
| **1700112E06Rik** | -1.80333 | 8.61E-05 |
| **Sfxn1** | -1.80463 | 0.0003 |
| **Cdc40** | -1.80506 | 0.000368 |
| **Naa35** | -1.80526 | 0.000456 |
| **Ap1g1** | -1.80531 | 0.000159 |
| **Ankrd28** | -1.80614 | 0.000583 |
| **Gm9781** | -1.8063 | 0.001063 |
| **Prhoxnb** | -1.8078 | 0.022321 |
| **Mkln1** | -1.80871 | 0.000586 |
| **Gtpbp4** | -1.809 | 0.000562 |
| **Vnn1** | -1.8091 | 0.001175 |
| **Mier1** | -1.81046 | 0.000292 |
| **Rmi1** | -1.81169 | 0.000317 |
| **Serpinb9** | -1.81295 | 0.000351 |
| **Zyg11b** | -1.81396 | 0.000815 |
| **Api5** | -1.81412 | 0.000298 |
| **Sec63** | -1.81468 | 0.000225 |
| **Mttp** | -1.81572 | 0.000214 |
| **Nedd4** | -1.8175 | 0.000283 |
| **Mib1** | -1.81803 | 0.000238 |
| **Cxcl9** | -1.81831 | 0.011441 |
| **D15Ertd621e** | -1.81912 | 0.000298 |
| **Pds5a** | -1.81926 | 0.00068 |
| **Hif1a** | -1.81928 | 0.000937 |
| **Mctp2** | -1.82206 | 0.000368 |
| **Bzw1** | -1.82227 | 0.00114 |
| **Usp34** | -1.8227 | 0.000666 |
| **Impa1** | -1.82273 | 0.000238 |
| **Manea** | -1.82572 | 0.00105 |
| **Herc4** | -1.82607 | 0.000583 |
| **Eri2** | -1.82609 | 0.001831 |
| **Met** | -1.82681 | 0.000478 |
| **Immt** | -1.82786 | 0.000338 |
| **Irgm1** | -1.82797 | 0.000527 |
| **4933411K20Rik** | -1.82812 | 0.000949 |
| **Cul3** | -1.82859 | 0.000186 |
| **Xpo1** | -1.82947 | 0.000419 |
| **Usp25** | -1.83044 | 0.000244 |
| **A430107O13Rik** | -1.83056 | 0.000333 |
| **Vsig4** | -1.83088 | 0.000498 |
| **Bmpr1a** | -1.8311 | 0.000563 |
| **Prkaa2** | -1.83207 | 0.000647 |
| **Smu1** | -1.83466 | 0.000494 |
| **Cdc16** | -1.83474 | 0.000292 |
| **Nsa2** | -1.83644 | 0.000185 |
| **Arhgap29** | -1.83647 | 0.001253 |
| **Elovl2** | -1.83793 | 0.000309 |
| **Mir568** | -1.83799 | 0.001147 |
| **Prpf39** | -1.83804 | 0.000947 |
| **Supt16h** | -1.83807 | 0.000347 |
| **Birc3** | -1.83899 | 0.00066 |
| **Abcb7** | -1.83928 | 0.000986 |
| **Nrd1** | -1.83957 | 0.000276 |
| **Yme1l1** | -1.83979 | 0.001259 |
| **Zfp719** | -1.84027 | 0.000295 |
| **Zfp266** | -1.84134 | 0.000496 |
| **Ibtk** | -1.84205 | 0.000539 |
| **Ddx3x** | -1.84226 | 0.000181 |
| **Gmps** | -1.84322 | 0.000343 |
| **Nmd3** | -1.8437 | 0.000533 |
| **Csde1** | -1.84438 | 0.000317 |
| **Afm** | -1.84535 | 0.000717 |
| **Sf3b1** | -1.8456 | 0.000537 |
| **Gramd3** | -1.84594 | 0.000533 |
| **Emcn** | -1.84796 | 0.0017 |
| **Cp** | -1.85081 | 0.000579 |
| **Dbt** | -1.85102 | 0.000583 |
| **Lims1** | -1.85391 | 0.000526 |
| **Mnda** | -1.8542 | 0.00493 |
| **Appl1** | -1.85493 | 0.001251 |
| **Wdr43** | -1.85528 | 0.000214 |
| **Capn7** | -1.85758 | 0.000463 |
| **Scrn3** | -1.85767 | 0.000351 |
| **Gm4951** | -1.85823 | 0.000518 |
| **Aldoc** | -1.85849 | 0.045699 |
| **Pkn2** | -1.86007 | 0.000298 |
| **Ddx3y** | -1.86039 | 0.00099 |
| **Pcmtd1** | -1.86059 | 0.000355 |
| **Kat2b** | -1.86088 | 0.000213 |
| **Tbce** | -1.86132 | 0.000298 |
| **Malat1** | -1.863 | 0.003971 |
| **Slc25a30** | -1.86322 | 0.029878 |
| **Dstn** | -1.8635 | 0.000308 |
| **Cpd** | -1.86668 | 0.000377 |
| **Rars** | -1.86764 | 0.00045 |
| **Nceh1** | -1.86859 | 0.000559 |
| **Zfp626** | -1.86923 | 0.000586 |
| **Skiv2l2** | -1.86961 | 0.000671 |
| **Snx13** | -1.87163 | 0.000309 |
| **4930518I15Rik** | -1.87727 | 0.000644 |
| **Akr1c14** | -1.88219 | 0.000968 |
| **Rora** | -1.88405 | 0.000497 |
| **Acsl3** | -1.88589 | 0.032161 |
| **Jak1** | -1.88868 | 0.000397 |
| **Dhx36** | -1.88872 | 0.001353 |
| **Rasgrp3** | -1.88983 | 0.000251 |
| **Rapgef4** | -1.89426 | 0.000328 |
| **E430025E21Rik** | -1.89509 | 0.000537 |
| **C8a** | -1.8958 | 0.00103 |
| **Scfd1** | -1.89618 | 0.000587 |
| **Osbpl9** | -1.89702 | 0.000298 |
| **Acsl5** | -1.89777 | 0.001682 |
| **Map3k7** | -1.89796 | 0.000172 |
| **Nudt12** | -1.89936 | 0.000417 |
| **Atp6v0d2** | -1.90055 | 0.039016 |
| **Trim30a** | -1.90099 | 0.000783 |
| **Gcap14** | -1.90179 | 0.000335 |
| **Zbtb41** | -1.90213 | 0.000269 |
| **Cmah** | -1.90316 | 0.000641 |
| **Prmt10** | -1.905 | 0.000178 |
| **Magt1** | -1.90533 | 0.000453 |
| **Fam126b** | -1.9058 | 0.00167 |
| **Trim30d** | -1.9066 | 0.001002 |
| **Ctdspl2** | -1.90935 | 0.000587 |
| **Snx6** | -1.90992 | 0.000486 |
| **A1cf** | -1.91026 | 0.000527 |
| **Rbm39** | -1.91111 | 0.00031 |
| **Aff4** | -1.91381 | 0.000244 |
| **Dkc1** | -1.91525 | 0.000317 |
| **Zmym5** | -1.91852 | 0.000995 |
| **Cdc14b** | -1.92175 | 0.000372 |
| **Nr3c1** | -1.92303 | 0.000218 |
| **Spcs3** | -1.92416 | 0.000214 |
| **Acbd5** | -1.92419 | 0.000372 |
| **Ifit1** | -1.92516 | 0.003528 |
| **Rbm12b** | -1.92676 | 0.019739 |
| **Fastkd2** | -1.92699 | 0.000298 |
| **Zfp187** | -1.92705 | 0.00069 |
| **Prkd3** | -1.92707 | 0.000234 |
| **Cyp7b1** | -1.92784 | 0.022528 |
| **AI987944** | -1.92822 | 0.004298 |
| **Smek2** | -1.93153 | 0.000583 |
| **Ube4a** | -1.93233 | 0.000264 |
| **Rarb** | -1.93546 | 0.002297 |
| **Serpina7** | -1.93706 | 0.023541 |
| **Abcb11** | -1.93764 | 9.38E-05 |
| **Dek** | -1.93766 | 0.000368 |
| **Cltc** | -1.94171 | 0.000743 |
| **Pja2** | -1.94315 | 0.002028 |
| **Ddx52** | -1.94374 | 0.000455 |
| **Zmym2** | -1.94402 | 0.000292 |
| **Cul4b** | -1.94457 | 0.00077 |
| **Slc10a2** | -1.94596 | 0.001978 |
| **Myo1b** | -1.94683 | 0.000225 |
| **C4bp** | -1.95003 | 0.000203 |
| **Stam2** | -1.95061 | 0.000138 |
| **Acot12** | -1.95115 | 0.000177 |
| **Ddx58** | -1.95144 | 0.000283 |
| **Cyp4a12a** | -1.95359 | 0.01583 |
| **Gpbp1** | -1.95601 | 0.000371 |
| **Srp72** | -1.9575 | 0.000185 |
| **Ddx1** | -1.95793 | 0.000298 |
| **Abce1** | -1.96095 | 0.000268 |
| **Appbp2** | -1.96165 | 0.000292 |
| **2610301B20Rik** | -1.96166 | 8.61E-05 |
| **C8b** | -1.96194 | 0.016973 |
| **Necab1** | -1.96563 | 0.000251 |
| **Sc4mol** | -1.96731 | 0.038103 |
| **Arfgef1** | -1.96843 | 0.000727 |
| **Pex3** | -1.96995 | 0.000317 |
| **Med13** | -1.97197 | 0.000178 |
| **Xiap** | -1.97249 | 0.000214 |
| **Dnm1l** | -1.97322 | 0.000516 |
| **Chrna4** | -1.97332 | 0.031034 |
| **Psme4** | -1.97436 | 0.000295 |
| **Gas2** | -1.97866 | 0.002205 |
| **F9** | -1.97959 | 0.001022 |
| **Klb** | -1.98202 | 0.000332 |
| **Tnks2** | -1.98317 | 8.61E-05 |
| **Slmap** | -1.98446 | 0.000296 |
| **Il1a** | -1.98672 | 0.000287 |
| **Ttc35** | -1.98701 | 0.000655 |
| **Pum2** | -1.9878 | 0.000619 |
| **Ppfibp1** | -1.98891 | 0.000214 |
| **Dmtf1** | -1.99103 | 0.000309 |
| **Hbp1** | -1.99203 | 0.000448 |
| **Rsl1d1** | -1.99328 | 0.000539 |
| **Nox4** | -1.99537 | 0.000658 |
| **Hspa13** | -1.99538 | 0.000493 |
| **Eps15** | -2.00158 | 0.000269 |
| **Etfdh** | -2.0047 | 0.000268 |
| **Cdkal1** | -2.0056 | 0.000298 |
| **Dpp4** | -2.00663 | 0.00045 |
| **Lrat** | -2.01842 | 0.000512 |
| **Efr3a** | -2.02115 | 0.000292 |
| **Tmed5** | -2.02316 | 0.00054 |
| **Chuk** | -2.02538 | 0.000298 |
| **Wwp1** | -2.02878 | 0.000377 |
| **Enpp2** | -2.02921 | 0.000276 |
| **Snx2** | -2.02951 | 0.000244 |
| **Fam175b** | -2.03322 | 0.000156 |
| **Mr1** | -2.03657 | 0.000663 |
| **Ccbl2** | -2.03847 | 0.000251 |
| **Mki67ip** | -2.03861 | 0.00054 |
| **Papola** | -2.0417 | 8.61E-05 |
| **Pnpla8** | -2.04304 | 0.000828 |
| **Ugt2b34** | -2.04454 | 0.000283 |
| **Ccdc50** | -2.04487 | 0.000295 |
| **Lifr** | -2.04703 | 0.026746 |
| **Lnpep** | -2.04986 | 0.000841 |
| **Nckap1** | -2.05202 | 0.00038 |
| **Pnrc2** | -2.0522 | 0.000295 |
| **Zranb1** | -2.05272 | 0.000283 |
| **Exoc6** | -2.05577 | 0.000716 |
| **Usp15** | -2.0598 | 0.000283 |
| **Trim12a** | -2.06491 | 0.001253 |
| **Tab2** | -2.06539 | 8.61E-05 |
| **Ifi44** | -2.06673 | 0.00384 |
| **Sh3bgrl** | -2.07256 | 0.000715 |
| **Pik3ca** | -2.0754 | 0.000268 |
| **Utp14b** | -2.08076 | 0.009436 |
| **Pter** | -2.08278 | 0.000244 |
| **Cpb2** | -2.08425 | 0.00072 |
| **Fam198a** | -2.08855 | 0.000298 |
| **Caprin1** | -2.08926 | 7.72E-05 |
| **Zwint** | -2.08934 | 0.000416 |
| **Ide** | -2.08939 | 0.001092 |
| **Aftph** | -2.09194 | 0.000152 |
| **Psmd1** | -2.09724 | 0.000251 |
| **Enpp3** | -2.10411 | 0.000218 |
| **Cyp2j6** | -2.10514 | 0.000234 |
| **Vps35** | -2.10715 | 0.000687 |
| **Gpam** | -2.11054 | 0.014604 |
| **Tug1** | -2.11687 | 0.000291 |
| **Ipo7** | -2.11784 | 0.001008 |
| **Acss2** | -2.11885 | 0.033799 |
| **Copb1** | -2.12095 | 0.00072 |
| **Wdr67** | -2.12142 | 0.004621 |
| **Cops4** | -2.12154 | 0.000324 |
| **Nae1** | -2.12227 | 0.000623 |
| **Ireb2** | -2.12938 | 0.000296 |
| **Ints12** | -2.13218 | 0.000281 |
| **Cul1** | -2.13359 | 0.000298 |
| **Hsp90b1** | -2.135 | 0.00046 |
| **Rnf125** | -2.13665 | 0.001848 |
| **Stard4** | -2.13892 | 0.000935 |
| **Cab39l** | -2.14363 | 0.000231 |
| **Ankrd49** | -2.1493 | 0.00077 |
| **Ifit3** | -2.15825 | 0.00042 |
| **Gm10002** | -2.16965 | 0.000112 |
| **Tank** | -2.17025 | 0.000251 |
| **Clcc1** | -2.17077 | 0.000145 |
| **Ceacam1** | -2.1714 | 8.61E-05 |
| **Ergic2** | -2.17172 | 0.000251 |
| **Acsl4** | -2.18539 | 0.001381 |
| **C6** | -2.19623 | 0.000553 |
| **Cse1l** | -2.21211 | 0.000654 |
| **Ppip5k2** | -2.21243 | 0.000178 |
| **Zfp146** | -2.21735 | 0.000186 |
| **Rc3h2** | -2.21795 | 0.000225 |
| **Bid** | -2.23361 | 6.3E-06 |
| **Fam45a** | -2.23512 | 0.000317 |
| **Crot** | -2.23796 | 0.000213 |
| **Orc3** | -2.24274 | 0.000377 |
| **Ppp2r3c** | -2.25359 | 0.000478 |
| **Cul2** | -2.27472 | 0.000317 |
| **5830473C10Rik** | -2.2753 | 0.000878 |
| **Mier3** | -2.2879 | 0.000267 |
| **Apobec1** | -2.3055 | 0.000192 |
| **Klhl24** | -2.30627 | 0.000523 |
| **Kpna3** | -2.31216 | 0.000448 |
| **Cyp4a12b** | -2.32288 | 0.034215 |
| **Cops2** | -2.3247 | 0.000738 |
| **Acsm3** | -2.32698 | 0.000636 |
| **F11** | -2.32802 | 0.000544 |
| **Dnajc3** | -2.33394 | 0.000112 |
| **C430048L16Rik** | -2.3357 | 0.000317 |
| **Psmc6** | -2.38805 | 0.000755 |
| **Iigp1** | -2.40056 | 0.000298 |
| **Clpx** | -2.41325 | 8.11E-05 |
| **Crbn** | -2.42365 | 0.000436 |
| **Vps41** | -2.43208 | 0.000295 |
| **Olfr1034** | -2.43591 | 0.000603 |
| **Gdap2** | -2.43762 | 0.000257 |
| **Cdh1** | -2.44825 | 0.001444 |
| **Camk1d** | -2.48195 | 0.000213 |
| **Serpina12** | -2.53222 | 0.000244 |
| **Gm11437** | -2.54602 | 0.000501 |
| **Gpcpd1** | -2.56488 | 0.001801 |
| **Pstpip2** | -2.57173 | 0.002815 |
| **Pnpt1** | -2.61943 | 0.00046 |
| **Fermt2** | -2.66559 | 8.61E-05 |
| **Mospd2** | -2.6695 | 0.000358 |
| **Nsdhl** | -2.68011 | 0.006941 |
| **Fam55b** | -2.73223 | 3.55E-05 |
| **Spp1** | -2.75633 | 0.001213 |
| **Gm9892** | -2.78604 | 0.001307 |
| **Gm10319** | -2.78959 | 0.000244 |
| **Clock** | -2.86173 | 0.000177 |
| **Gbp11** | -2.86473 | 0.036021 |
| **Mir122a** | -2.89954 | 0.000734 |
| **Rdh11** | -2.93278 | 0.011805 |
| **Tmem184c** | -2.94369 | 8.61E-05 |
| **Hmgcr** | -3.06575 | 0.01095 |
| **Mug-ps1** | -3.08477 | 0.000251 |
| **BC023105** | -3.46723 | 0.000671 |
| **Cyp51** | -3.69599 | 0.003356 |
| **Sqle** | -5.97064 | 0.005025 |
| **Mug2** | -9.11896 | 4.29E-10 |
