# Supplementary material for: 8-Oxoguanine DNA Glycosylase (OGG1) Deficiency Increases Susceptibility to Obesity and Metabolic Dysfunction
Source: PLoS One. 2012 Dec 17;7(12):e51697. doi: 10.1371/journal.pone.0051697 (PMC3524114; doi:10.1371/journal.pone.0051697)
Supplement: Table S5 — KEGG Pathway analysis of DEPs in chow-fed Ogg1−/− livers. DEPs that were altered by at least 1.4 fold in chow-fed Ogg1−/− livers, relative to WT livers, were annotated using the Kyoto Encyclopedia of Genes and Genomes (KEGG) in GeneSifter. All pathways with an associated z-score >2.0 or <−2.0 are presented. n = 6 per group. n = 6 in each group. (DOC) [file pone.0051697.s006.doc]

| **KEGG Pathway** | **List of DEPS** | **Up** | **Down** | **Gene Set** | **z-score (Up)** | **z-score (Down)** |
| --- | --- | --- | --- | --- | --- | --- |
| **Circadian rhythm - mammal** | 2 | 1 | 1 | 21 | 8.54 | 6.93 |
| **Adipocytokine signaling pathway** | 1 | 0 | 1 | 66 | -0.21 | 3.75 |
| **Alzheimer's disease** | 1 | 0 | 1 | 161 | -0.32 | 2.18 |
| **Amyotrophic lateral sclerosis (ALS)** | 1 | 0 | 1 | 55 | -0.19 | 4.15 |
| **Apoptosis** | 1 | 0 | 1 | 85 | -0.23 | 3.25 |
| **Base excision repair** | 1 | 0 | 1 | 34 | -0.15 | 5.38 |
| **Cell cycle** | 1 | 1 | 0 | 123 | 3.32 | -0.35 |
| **Glycerophospholipid metabolism** | 1 | 0 | 1 | 76 | -0.22 | 3.46 |
| **Lysosome** | 1 | 1 | 0 | 119 | 3.39 | -0.34 |
| **Natural killer cell mediated cytotoxicity** | 1 | 0 | 1 | 134 | -0.3 | 2.46 |
| **p53 signaling pathway** | 1 | 0 | 1 | 67 | -0.21 | 3.72 |
| **Protein processing in endoplasmic reticulum** | 1 | 1 | 0 | 163 | 2.82 | -0.4 |
| **Purine metabolism** | 1 | 0 | 1 | 160 | -0.32 | 2.19 |
| **Selenoamino acid metabolism** | 1 | 0 | 1 | 23 | -0.12 | 6.61 |
| **Sulfur metabolism** | 1 | 0 | 1 | 10 | -0.08 | 10.14 |
| **Viral myocarditis** | 1 | 0 | 1 | 80 | -0.23 | 3.36 |

**Supporting Table S5**: **KEGG Pathway analysis of DEPs in chow-fed *Ogg1^-/-^* livers**
