# Supplementary material for: 8-Oxoguanine DNA Glycosylase (OGG1) Deficiency Increases Susceptibility to Obesity and Metabolic Dysfunction
Source: PLoS One. 2012 Dec 17;7(12):e51697. doi: 10.1371/journal.pone.0051697 (PMC3524114; doi:10.1371/journal.pone.0051697)
Supplement: Table S6 — KEGG Pathway analysis of DEPs in HFD-fed Ogg1−/− livers. DEPs that were altered by at least 1.4 fold in HFD-fed Ogg1−/− livers, relative to WT livers, were annotated using the Kyoto Encyclopedia of Genes and Genomes (KEGG) in GeneSifter. All pathways with an associated z-score >2.0 or <−2.0 are presented. n = 6 per group. (DOC) [file pone.0051697.s007.doc]

**Supporting Table S6**: **KEGG Pathway analysis of DEPs in HFD-fed *Ogg1^-/-^* livers**

| **KEGG Pathway** | **List of DEPS** | **Up** | **Down** | **Gene Set** | **z-score (Up)** | **z-score (Down)** |
| --- | --- | --- | --- | --- | --- | --- |
| **Metabolic pathways** | 123 | 39 | 84 | 1112 | 2.5 | -1.08 |
| **Pathways in cancer** | 46 | 8 | 38 | 318 | 0.07 | 2.37 |
| **RNA transport** | 39 | 8 | 31 | 157 | 2.16 | 5.21 |
| **Ubiquitin mediated proteolysis** | 36 | 7 | 29 | 134 | 2.09 | 5.61 |
| **Endocytosis** | 32 | 3 | 29 | 212 | -1 | 2.84 |
| **Protein processing in endoplasmic reticulum** | 32 | 6 | 26 | 163 | 1.02 | 3.54 |
| **Spliceosome** | 27 | 12 | 15 | 122 | 5.32 | 1.58 |
| **Hepatitis C** | 26 | 3 | 23 | 133 | -0.15 | 3.76 |
| **Insulin signaling pathway** | 24 | 6 | 18 | 134 | 1.53 | 2.14 |
| **Osteoclast differentiation** | 22 | 5 | 17 | 114 | 1.34 | 2.55 |
| **RNA degradation** | 22 | 4 | 18 | 69 | 1.8 | 5.34 |
| **Toll-like receptor signaling pathway** | 22 | 4 | 18 | 99 | 1.03 | 3.55 |
| **Complement and coagulation cascades** | 21 | 3 | 18 | 73 | 0.92 | 5.05 |
| **mRNA surveillance pathway** | 21 | 4 | 17 | 81 | 1.45 | 4.13 |
| **Chagas disease (American trypanosomiasis)** | 19 | 4 | 15 | 101 | 0.99 | 2.37 |
| **Prostate cancer** | 19 | 4 | 15 | 88 | 1.28 | 2.96 |
| **T cell receptor signaling pathway** | 19 | 3 | 16 | 108 | 0.22 | 2.44 |
| **TGF-beta signaling pathway** | 19 | 4 | 15 | 83 | 1.4 | 3.21 |
| **Small cell lung cancer** | 18 | 4 | 14 | 84 | 1.38 | 2.76 |
| **Renal cell carcinoma** | 16 | 3 | 13 | 71 | 0.97 | 3.04 |
| **RIG-I-like receptor signaling pathway** | 16 | 1 | 15 | 68 | -0.53 | 4.1 |
| **Adherens junction** | 15 | 1 | 14 | 73 | -0.6 | 3.35 |
| **B cell receptor signaling pathway** | 15 | 2 | 13 | 75 | 0.12 | 2.82 |
| **mTOR signaling pathway** | 15 | 3 | 12 | 52 | 1.55 | 3.84 |
| **NOD-like receptor signaling pathway** | 15 | 1 | 14 | 61 | -0.41 | 4.13 |
| **Pancreatic cancer** | 15 | 3 | 12 | 70 | 1 | 2.66 |
| **Systemic lupus erythematosus** | 15 | 9 | 6 | 109 | 3.95 | -1.09 |
| **Chronic myeloid leukemia** | 14 | 3 | 11 | 72 | 0.94 | 2.13 |
| **Melanoma** | 14 | 3 | 11 | 70 | 1 | 2.23 |
| **Acute myeloid leukemia** | 13 | 4 | 9 | 56 | 2.28 | 2.09 |
| **Non-small cell lung cancer** | 13 | 3 | 10 | 53 | 1.51 | 2.77 |
| **Aminoacyl-tRNA biosynthesis** | 11 | 2 | 9 | 42 | 0.97 | 3.07 |
| **Bacterial invasion of epithelial cells** | 11 | 0 | 11 | 68 | -1.32 | 2.34 |
| **Endometrial cancer** | 11 | 2 | 9 | 52 | 0.65 | 2.34 |
