# Supplementary material for: 8-Oxoguanine DNA Glycosylase (OGG1) Deficiency Increases Susceptibility to Obesity and Metabolic Dysfunction
Source: PLoS One. 2012 Dec 17;7(12):e51697. doi: 10.1371/journal.pone.0051697 (PMC3524114; doi:10.1371/journal.pone.0051697)
Supplement: Methods S1 — Supporting Methods. (DOC) [file pone.0051697.s008.doc]

**Supporting Methods**

*Electron microscopy*

Fresh liver was minced on ice to 1 mm^3^ pieces in buffer containing 1.5% glutaraldehyde, 1.5% paraformaldehyde in 0.1M sodium cacodylate buffer, pH 7.4 with 0.05M sucrose and 0.25% CaCl2. After 2 hours of fixation at 4 deg C, tissues were transferred to buffer containing 0.1M sodium cacodylate and submitted to the electron microscopy core at OHSU for sectioning and visualization.

*DNA microarray*

Microarray experiments were conducted in the Affymetrix Microarray Core of the OHSU Gene Microarray Shared Resource. Gene expression was measured using the Mouse Gene ST GeneChip Array (Affymetrix, Inc., Santa Clara, Calif, [www.affymetrix.com](http://www.affymetrix.com/)). This is a single, high-density oligonucleotide array that contains perfect match (PM) probe sets representing transcripts from approximately 28,853 mouse genes. The procedures were as follows. 125 ng of total RNA was labeled, as described in the Ambion WT Expression kit Technical Manual: http://www.ambion.com/catalog/CatNum.php?4411974. The assay converts total RNA to sense-strand cDNA in three steps: (1) First strand synthesis: the total RNA was converted to cDNA using random primers with a T7 RNA polymerase binding site that selectively reverse transcribes non-ribosomal RNA (2) Second strand cDNA was generated using DNA Polymerase and was then converted to antisense cRNA in an *in vitro*-transciption reaction using T7 RNA polymerase (3) the antisense cRNA was then converted to sense strand cDNA with a dUTP incorporation. Fragmentation and labeling was performed using the Affymetrix GeneChip WT Terminal Labeling kit. Uniform cDNA fragmentation was achieved by utilizing the dUTP and a combination of uracil DNA glycosylase and apurinic/apyrimidinic endonuclease 1. The fragmented DNA was then labeled using terminal deoxynucleotidyl transferase in the presence of a proprietary biotinylated compound, GeneChip DNA Labeling Reagent Prior to hybridization to a GeneChip genome array, target quality was assessed by examining the yield of both the cRNA and single-stranded cDNA conversion using the Nanodrop 8000. Samples that failed quality control were discarded or relabeled. The fragmented and labeled sense-strand DNA was combined with biotinylated hybridization control oligomer and biotinylated control cRNAs for BioB, BioC, BioD and CreX (Affymetrix) in hybridization buffer. 1.8 μg of target was hybridized with the GeneChip Mouse Gene ST Array (Affymetrix) overnight, followed by washing, staining with streptavidin-phycoerythrin (Affymetrix), signal amplification with biotinylated anti-streptavidin antibody (Affymetrix), and a final staining step on the Fluidics Station 450 (Affymetrix). The distribution of fluorescent material on the processed array was determined using the GeneChip 3000 laser scanner (Affymetrix) with the 7G upgrade. Image inspection was performed manually immediately following each scan. The array image scan was processed with the Affymetrix GeneChip Command Console (AGCC) software. The GeneChip expression arrays contain control probe sets for both spiked and endogenous RNA transcripts (e.g., BioB, BioC, BioD, CreX and species-specific actin and GAPDH). Following image processing, array data was uploaded to the Affymetrix Expression Console for further processing and quality assessment. Three primary values were derived in Expression Console to assess overall assay performance: Pos_vs_neg_auc (a score of the detection of positive controls against the false detection of negative controls), Probe Set Mean, and the Probe Set RLE Mean (signal of each probe set was compared to the median signal value of this probe set in the study; mean of differences was calculated). In addition, the background mean and signal for hybridization control spikes (BioC, BioD and CreX) were monitored for consistency across the data set. Data distribution patterns were inspected using boxplots (probe level and gene level) and scatter plots. Assays demonstrating poor, marginal or unusual performance were flagged. Data visualization and exploratory analysis were conducted using GeneSifter web-based software, release 59.0 (GeoSpiza Inc., Seattle, WA). Affymetrix ".cel" files were uploaded to the GeneSifter web site with GC robust multiarray average (RMA) normalization. Correction for multiple testing was performed using the method of Benjamini and Hochberg (Benjamini and Hochberg, 1995) to derive a false discovery rate estimate from the raw p-values, and adjusted p-values less than or equal to 0.05 were considered statistically significant. Pairwise analysis of WT Chow vs. *Ogg1^-/-^* Chow and WT HFD vs. *Ogg1^-/-^* HFD was performed by t-test between the groups, followed by a Benjamini and Hochberg adjustment used to correct for false discovery rates. Tests for enrichment of common function among sets of differentially-expressed probe sets (DEPs) that were altered by at least 1.4-fold were carried out using data from the Gene Ontology (GO) annotations and the Kyoto Encyclopedia of Genes and Genomes (KEGG) in GeneSifter Kyoto Encyclopedia of Genes and Genomes (KEGG) Gene Ontology (GO) reports were utilized to evaluate potential relationships between differentially expressed genes. KEGG and GO reports were evaluated by z-scores, with z-Scores greater than or equal to +2.0 or less than or equal to –2.0 taken as suggestive of biological significance, indicating that genes associated with a pathway appear either more (z score > 2.0) or less (z-score < -2.0) frequently in the experimental group (*Ogg1^-/-^*), compared to the control group (WT). z-Scores were calculated by GeneSifter with the formula z = (r – n R/N)/√{n(R/N)(1 – R/N)[1 – (n – 1/N – 1)]}, where R is the total number of genes meeting selection criteria, N is the total number of genes measured, r is the number of genes meeting selection criteria with the specified pathway, n is the total number of genes measured within a specific pathway. CEL files and probe set signals have been deposited in NCBI's Gene Expression Omnibus and are accessible through GEO Series accession number GSE35497. Complete lists of significant gene expression changes from Gene Sifter analyses are presented in Supporting tables S1 and S2.

In addition to data analysis by GeneSifter, the Affymetrix data was simultaneously submitted to Ingenuity Systems for analysis via Ingenuity iReport for Gene Expression Analysis. Ingenuity Pathways conducts gene expression analysis of microarray data using a Limma technique followed by filtering for statistical significance using Benjamini and Hochberg’s False Discovery Rate (FDR), with an FDR-adjusted p-value cutoff of 0.05. Further details on Ingenuity iReport Statistical Methods are available at <http://www.ingenuity.com/getireport/pdf/ingenuity_robust_unattended_microarray_analysis.pdf>. Complete lists of significant gene expression changes from iReport analyses are presented in Supporting tables S3 and S4.
